# Supplementary material for: Single-cell RNA sequencing reveals placental response under environmental stress
Source: Nat Commun. 2024 Aug 2;15:6549. doi: 10.1038/s41467-024-50914-9 (PMC11297347; doi:10.1038/s41467-024-50914-9)
Supplement: Supplementary file 1 — Supplementary Information [file 41467_2024_50914_MOESM1_ESM.pdf]

Supplementary Information

Single-cell RNA sequencing reveals placental response under environmental stress

Eric Van Buren[1], David Azzara[2], Javier Rangel-Moreno[3], Maria de la Luz Garcia-Hernandez[3], Shawn P. Murphy[4], Ethan D. Cohen[5], Ethan Lewis[2], Xihong Lin[1,6], Hae-Ryung Park[2,*]

[1]Department of Biostatistics, Harvard School of Public Health, Boston, MA, USA

[2]Department of Environmental Medicine, School of Medicine and Dentistry, University of Rochester, Rochester, NY, USA

[3]Division of Allergy, Immunology and Rheumatology, Department of Medicine, University of Rochester, Rochester, NY, USA

[4]Department of Obstetrics and Gynecology, School of Medicine and Dentistry, University of Rochester, Rochester, NY, USA

[5]Department of Pediatrics, School of Medicine and Dentistry, University of Rochester, Rochester, NY, USA

[6]Department of Statistics, Harvard University, Cambridge, MA, USA

***Correspondence addressed to:**

Hae-Ryung Park
Department of Environmental Medicine
School of Medicine and Dentistry
University of Rochester
601 Elmwood Ave
Rochester, NY 14642
Email: hae-ryung_park@urmc.rochester.edu

Supplementary Figures

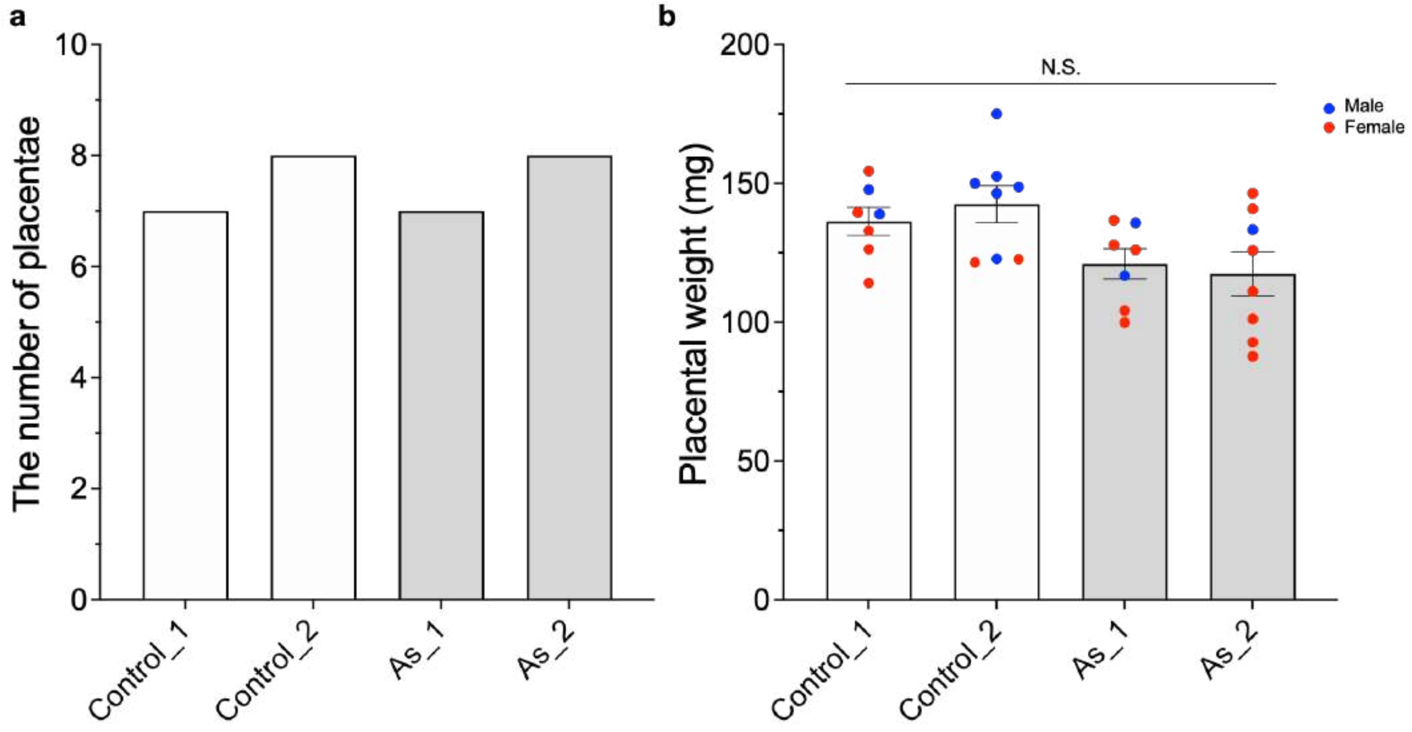

Supplementary Fig. 1. The number of (a) placentae and (b) placental weights used in scRNA-seq analysis.

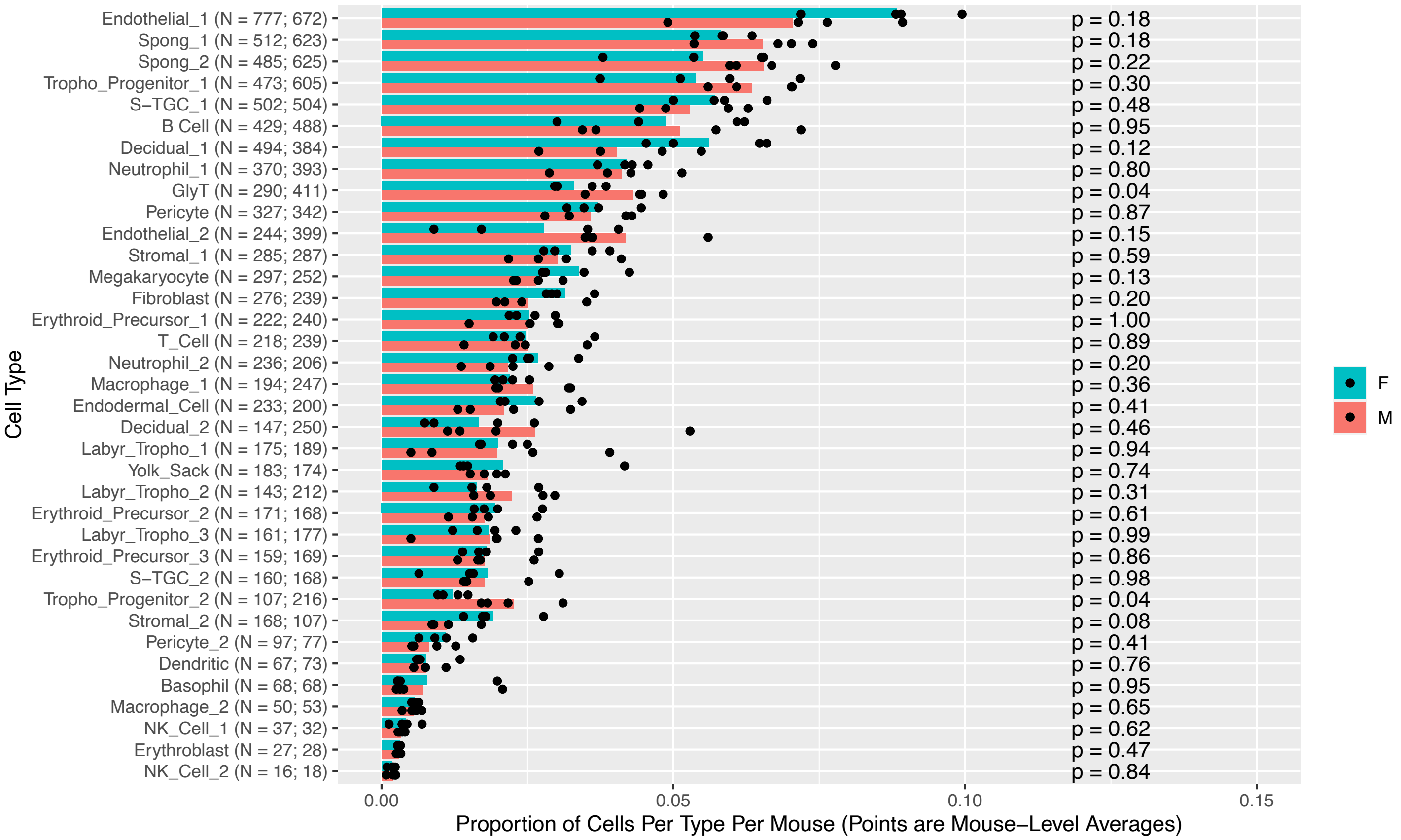

Supplementary Fig. 2. Proportion of male (M) and female (F) cells in each cluster, along with p-values comparing male to female proportions within a cell type.

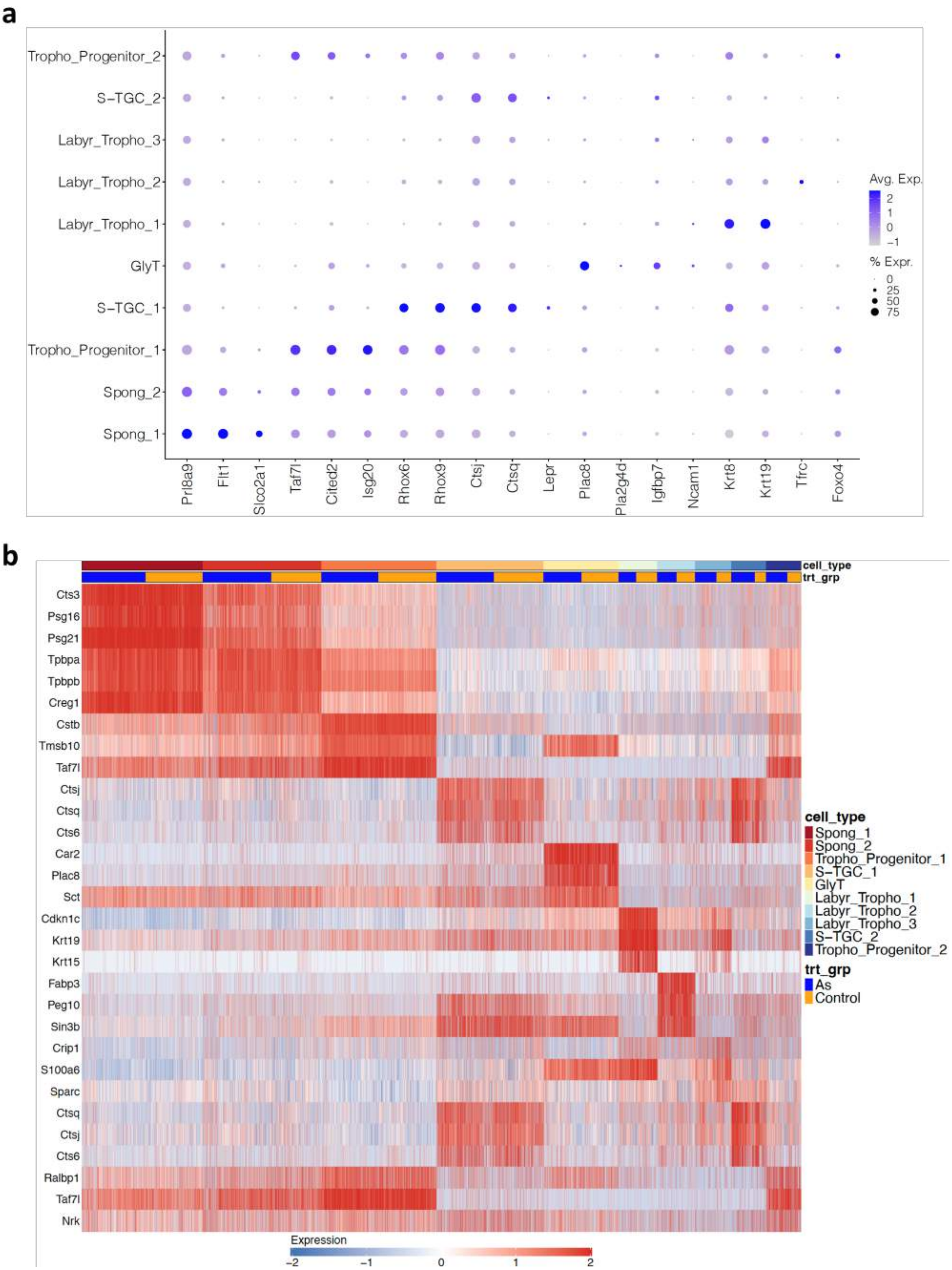
a
Tropho_Progenitor_2
S-TGC_2
Labyr_Tropho_3
Labyr_Tropho_2
Labyr_Tropho_1
GlyT
S-TGC_1
Tropho_Progenitor_1
Spong_2
Spong_1
Prl8a9
Flt1
Slco2a1
Taf7l
Cited2
Isg20
Rhox6
Rhox9
Ctsj
Ctsq
Lepr
Plac8
Pla2g4d
Igfbp7
Ncam1
Krt8
Krt19
Tfrc
Foxo4
Avg. Exp.
2
1
0
−1
% Expr.
0
25
50
75
b
cell_type
trt_grp
Cts3
Psg16
Psg21
Tpbpa
Tpbpb
Creg1
Cstb
Tmsb10
Taf7l
Ctsj
Ctsq
Cts6
Car2
Plac8
Sct
Cdkn1c
Krt19
Krt15
Fabp3
Peg10
Sin3b
Crip1
S100a6
Sparc
Ctsq
Ctsj
Cts6
Ralbp1
Taf7l
Nrk
cell_type
Spong_1
Spong_2
Tropho_Progenitor_1
S-TGC_1
GlyT
Labyr_Tropho_1
Labyr_Tropho_2
Labyr_Tropho_3
S-TGC_2
Tropho_Progenitor_2
trt_grp
As
Control
Expression
−2
−1
0
1
2

Supplementary Fig. 3. Identification of each cell type by marker genes in mouse placenta (trophoblasts) (a) Dot plot showing average expression and percent of cells in each cluster expressing marker genes. The color key from light blue to dark blue suggests low to high gene expression levels while the percentage of cells positive for a given marker is positively correlated with the node size. (b) Heatmap with marker genes on the y-axis and cell-types and treatment groups on the x-axis.

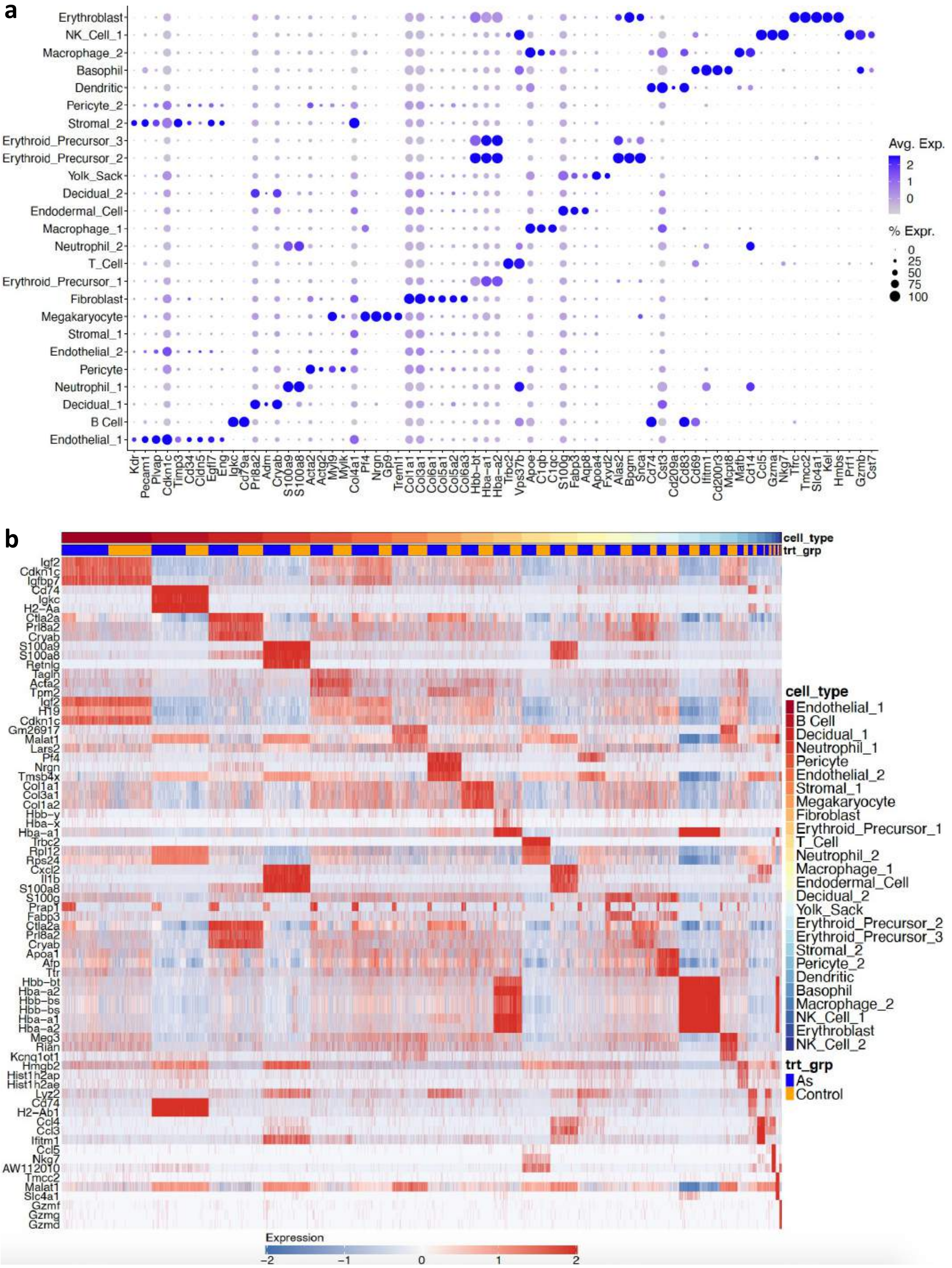
a
Erythroblast
NK_Cell_1
Macrophage_2
Basophil
Dendritic
Pericyte_2
Stromal_2
Erythroid_Precursor_3
Erythroid_Precursor_2
Yolk_Sack
Decidual_2
Endodermal_Cell
Macrophage_1
Neutrophil_2
T_Cell
Erythroid_Precursor_1
Fibroblast
Megakaryocyte
Stromal_1
Endothelial_2
Pericyte
Neutrophil_1
Decidual_1
B Cell
Endothelial_1
Kdr Pecam1 Plvap Cdkn1c Timp3 Cd34 Cldn5 Egfl7 Eng Igkc Cd79a Prl8a2 Adm Cryab S100a9 S100a8 Acta2 Actg2 Myl9 Mylk Col4a1 Pf4 Nrgn Gp9 Treml1 Col1a1 Col3a1 Col6a1 Col5a1 Col5a2 Col6a3 Hbb-bt Hba-a1 Hba-a2 Trbc2 Vps37b Apoe C1qb C1qc S100g Fabp3 Aqp8 Apoa4 Fxyd2 Alas2 Bpgm Snca Cd74 Cst3 Cd209a Cd83 Cd69 Ifitm1 Cd200r3 Mcpt8 Mafb Cd14 Ccl5 Gzma Nkg7 Tfrc Tmcc2 Slc4a1 Kel Hmbs Prf1 Gzmb Cst7
Avg. Exp.
2
1
0
% Expr.
0
25
50
75
100
b
cell_type
trt_grp
Igf2 Cdkn1c Igfbp7 Cd74 Igkc H2-Aa Ctla2a Prl8a2 Cryab S100a9 S100a8 Retnlg Tagln Acta2 Tpm2 Igf2 H19 Cdkn1c Gm26917 Malat1 Lars2 Pf4 Nrgn Tmsb4x Col1a1 Col3a1 Col1a2 Hbb-y Hba-x Hba-a1 Trbc2 Rpl12 Rps24 Cxcl2 Il1b S100a8 S100g Prap1 Fabp3 Ctla2a Prl8a2 Cryab Apoa1 Afp Ttr Hbb-bt Hba-a2 Hbb-bs Hbb-bs Hba-a1 Hba-a2 Meg3 Rian Kcnq1ot1 Hmgb2 Hist1h2ap Hist1h2ae Lyz2 Cd74 H2-Ab1 Ccl4 Ccl3 Ifitm1 Ccl5 Nkg7 AW112010 Tmcc2 Malat1 Slc4a1 Gzmf Gzmg Gzmd
cell_type
Endothelial_1
B Cell
Decidual_1
Neutrophil_1
Pericyte
Endothelial_2
Stromal_1
Megakaryocyte
Fibroblast
Erythroid_Precursor_1
T_Cell
Neutrophil_2
Macrophage_1
Endodermal_Cell
Decidual_2
Yolk_Sack
Erythroid_Precursor_2
Erythroid_Precursor_3
Stromal_2
Pericyte_2
Dendritic
Basophil
Macrophage_2
NK_Cell_1
Erythroblast
NK_Cell_2
trt_grp
As
Control
Expression
−2
−1
0
1
2

Supplementary Fig. 4. Identification of each cell type by marker genes in mouse placenta (non-trophoblasts) (a) Dot plot showing average expression and percent of cells in each cluster expressing marker genes. The color key from light blue to dark blue suggests low to high gene expression levels while the percentage of cells positive for a given marker is positively correlated with the node size. (b) Heatmap with marker genes on the y-axis and cell-types and treatment groups on the x-axis.

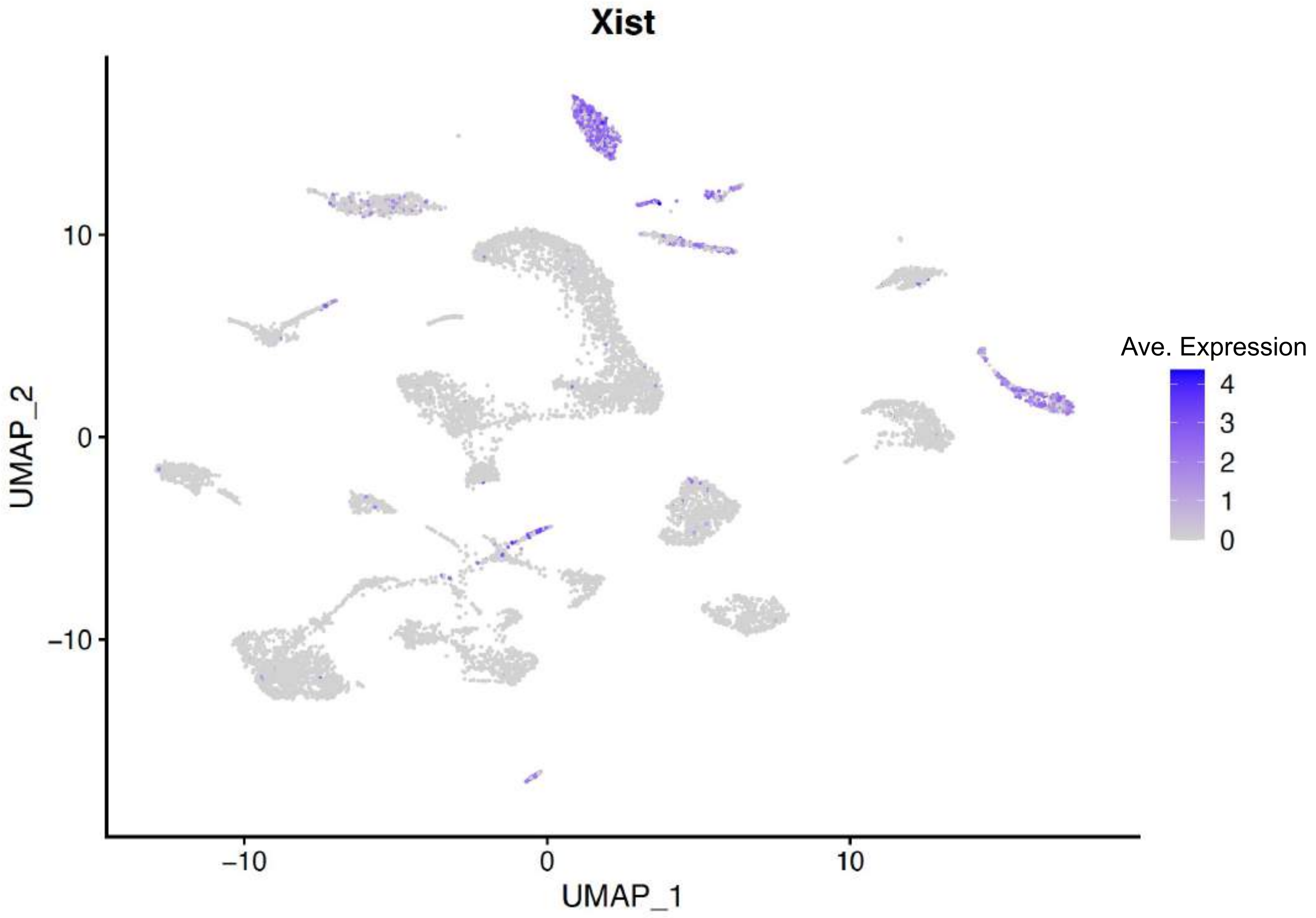

Supplementary Fig. 5. Expression of *Xist* in male placentae. Male placental samples express *Xist* only in maternal cells.

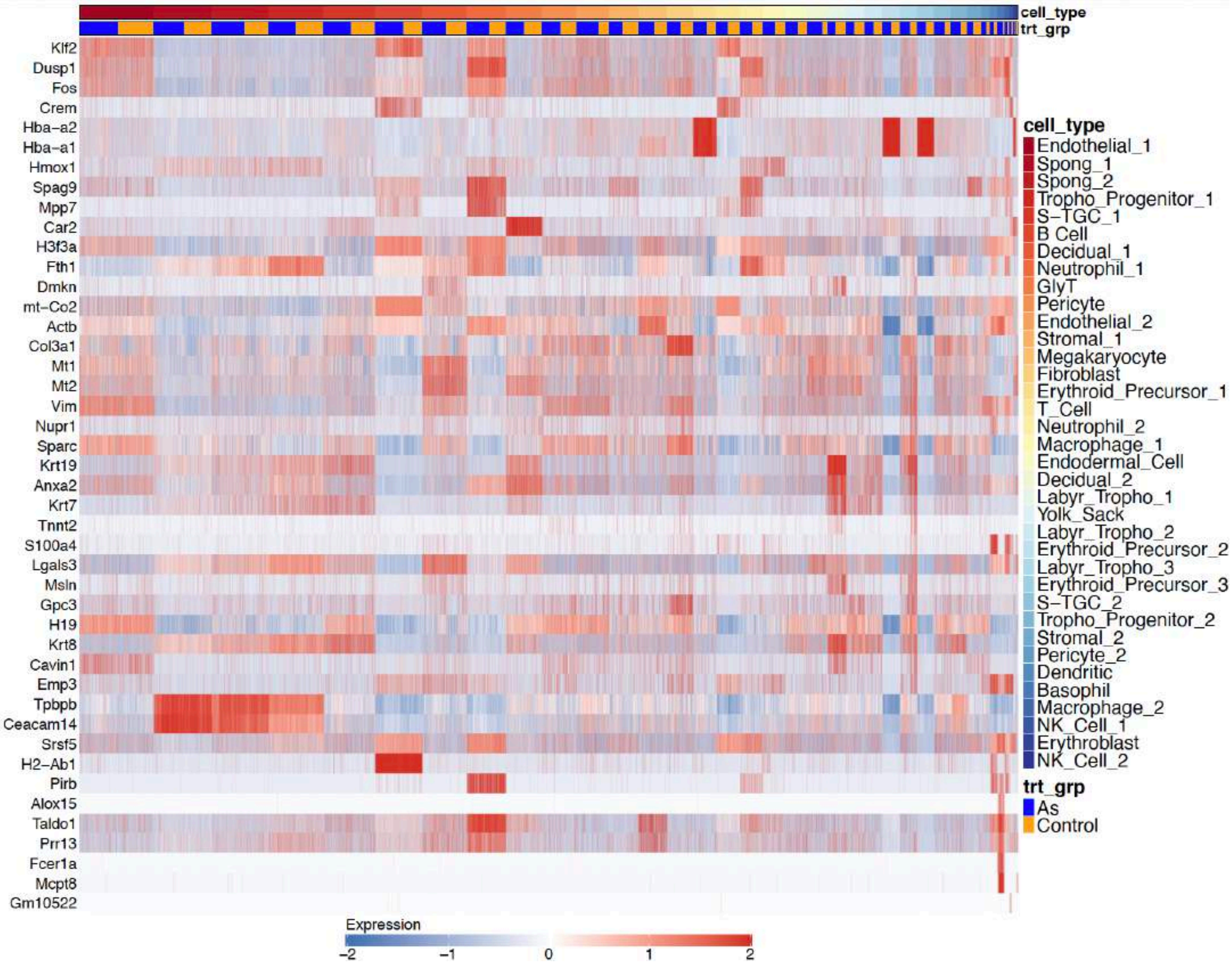

Supplementary Fig. 6. Heatmap showing the expression of unique DE genes, defined as genes whose DE *p*-values only attained significance in one cluster.

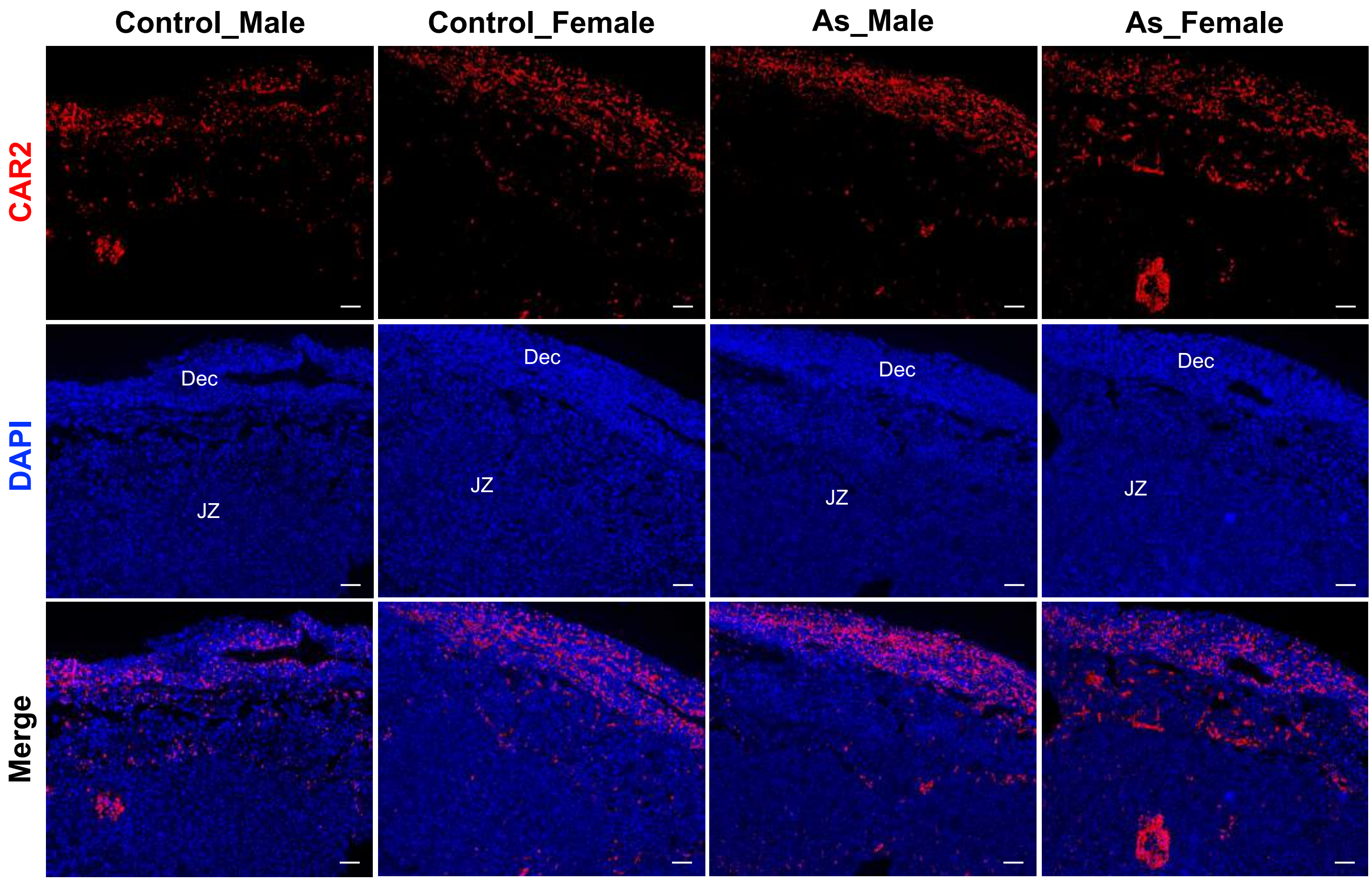

Supplementary Fig. 7. Immunofluorescence staining of CAR2 in mouse placenta. Scale bars represent 100 μm. As: Arsenic; Dec: Decidua; 2. JZ: Junctional Zone.

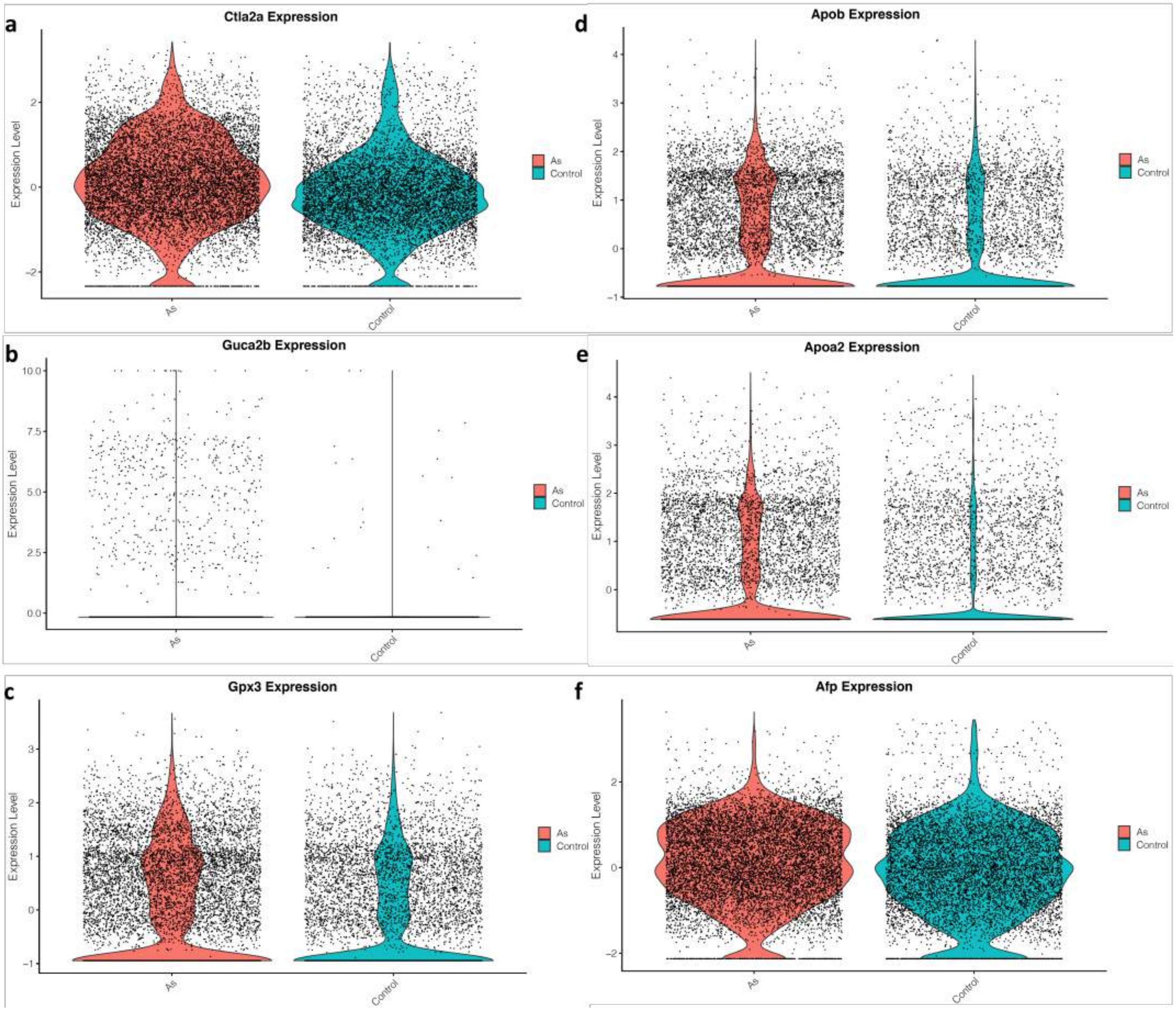

Supplementary Fig. 8. Violin plots showing the expression of select DE genes by treatment. (a) *Ctla2a*, (b) *Guca2b*, (c) *Gpx3*, (d) *Apob*, (e) *Apoa2*,and (f) *Afp*. As: Arsenic.

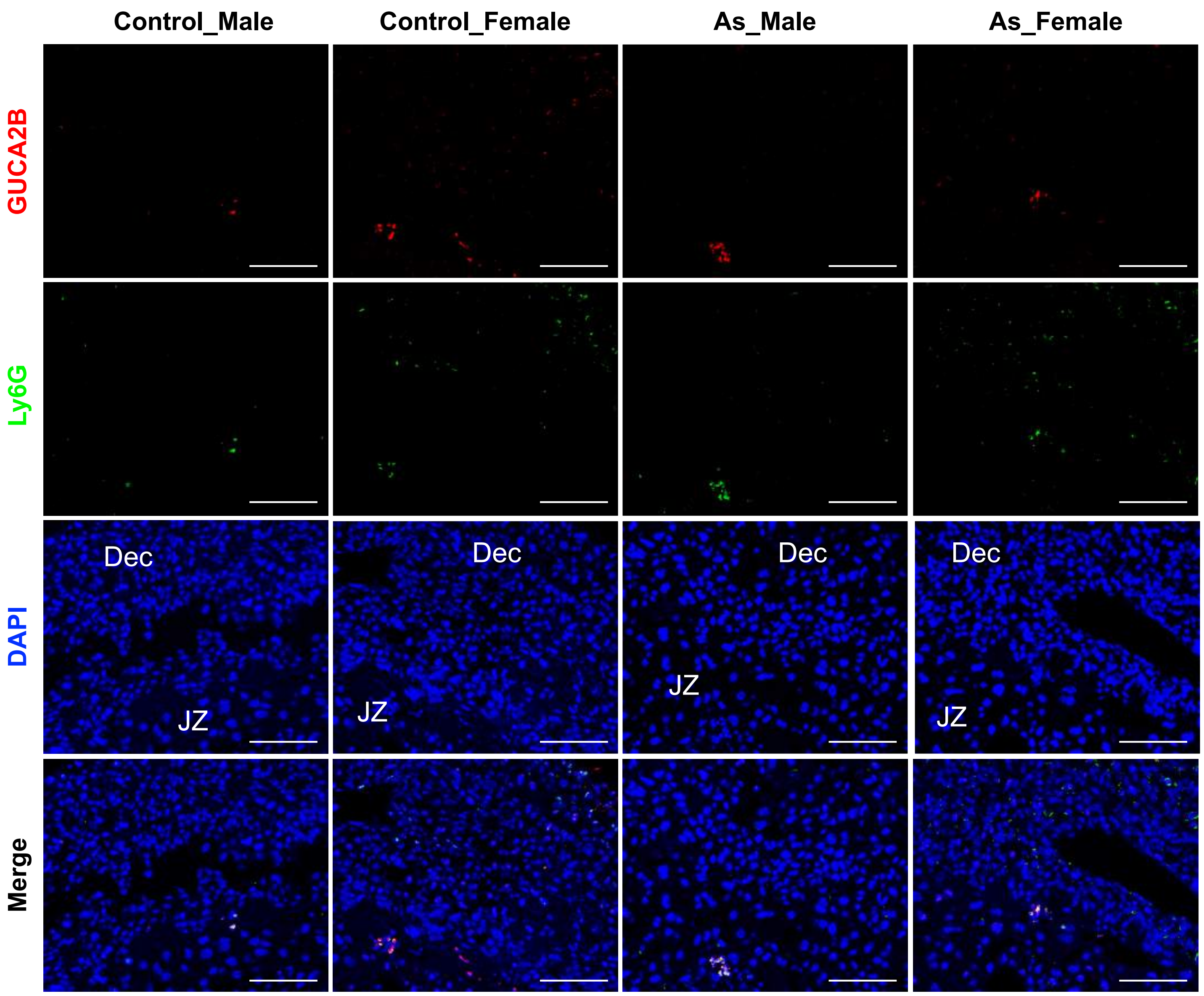

Supplementary Fig. 9. Immunofluorescence staining of GUCA2B in mouse placenta. Scale bars represent 100 μm. As: Arsenic; Dec: Decidua; 2. JZ: Junctional Zone.

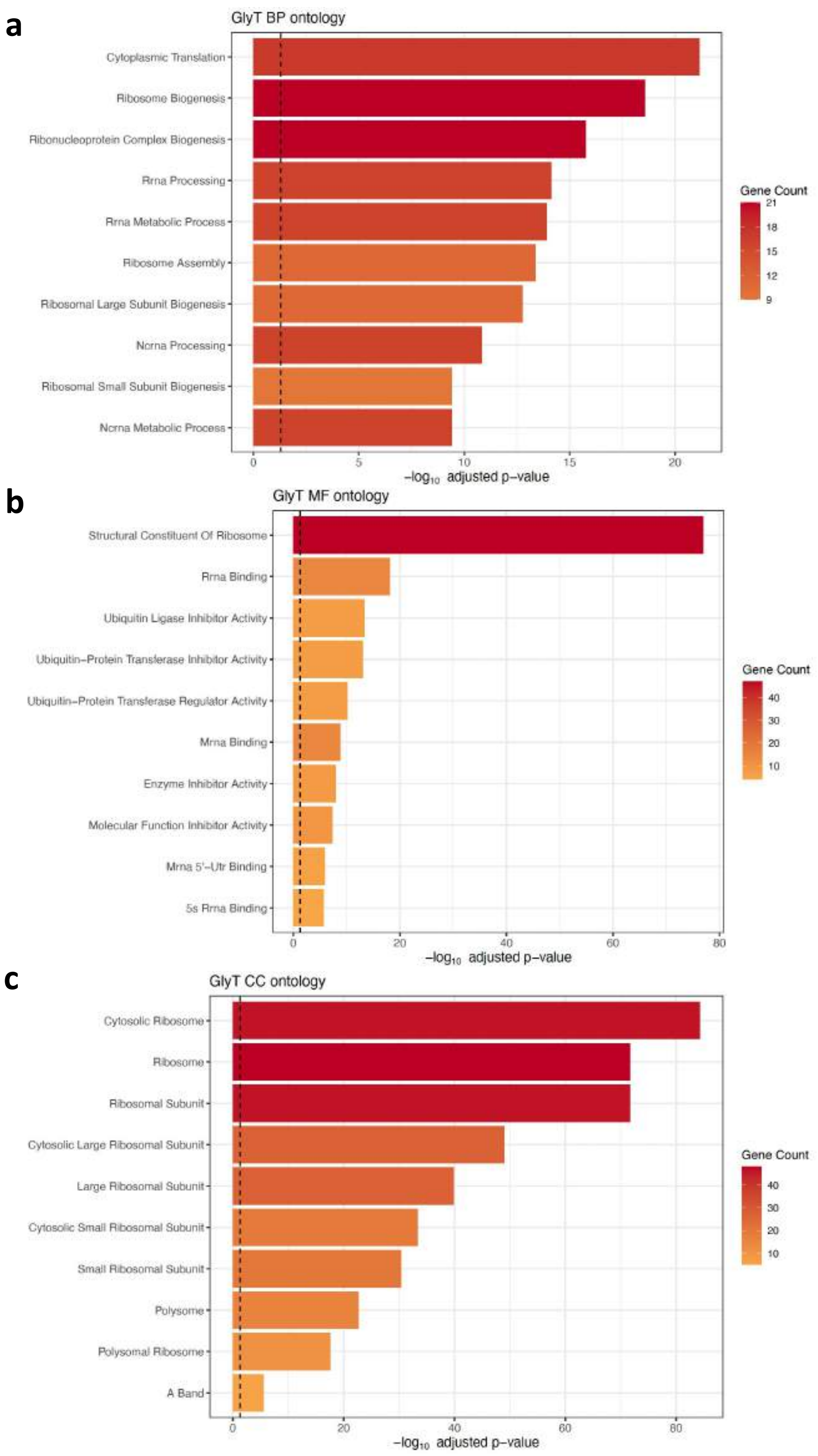

Supplementary Fig. 10. GO term analyses for GlyT. (a) Biological Processes (BP), (b) Molecular Function (MF), (c) Cellular Component (CC). GO term analyses are based on the hypergeometric test, with two-sided p-values adjusting for multiple comparisons via the Benjamini-Hochberg method

to control the False Discovery Rate. Full GO analysis results for all cell types are available in Supplementary Figures 12 and 13.

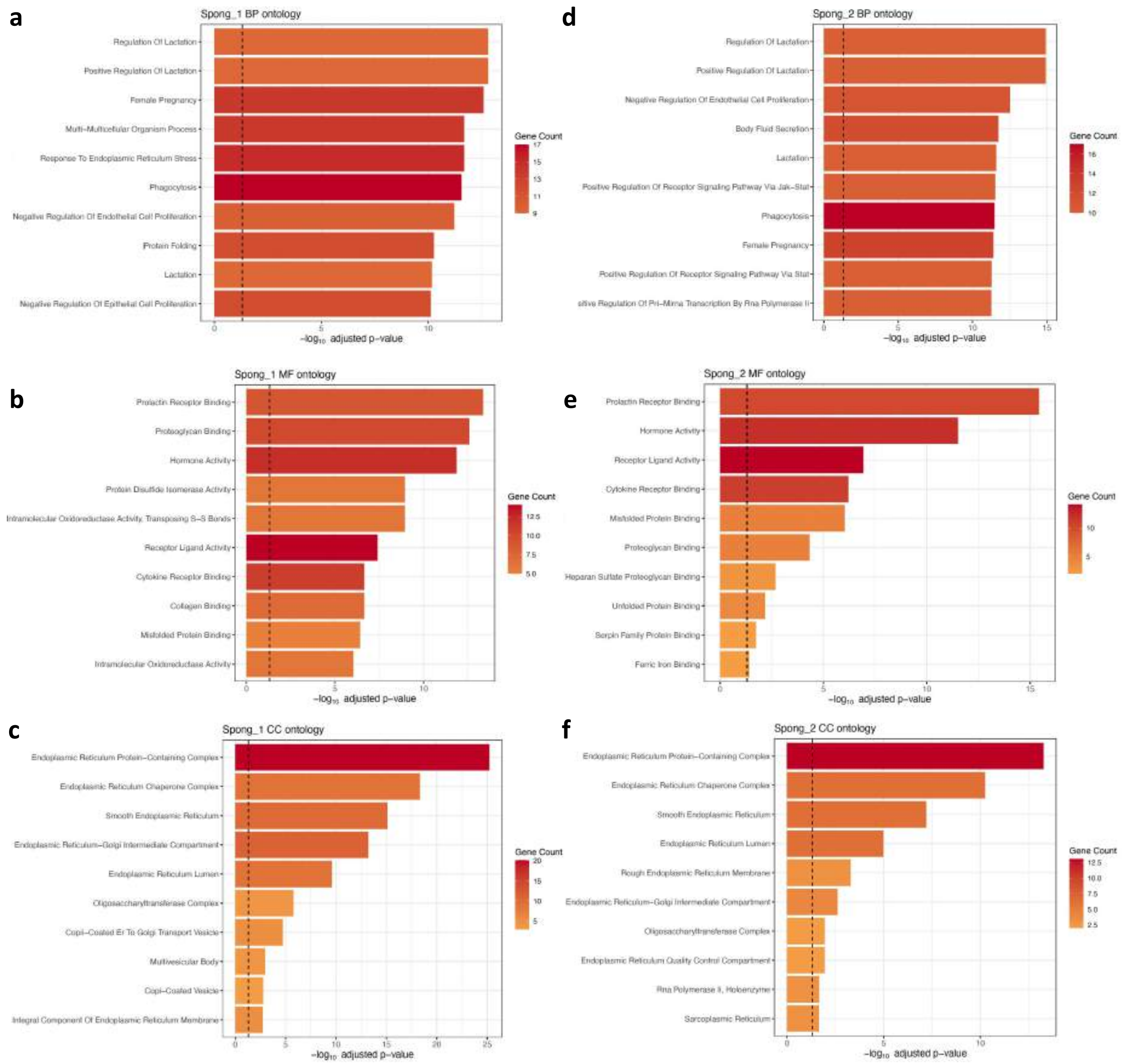

Supplementary Fig. 11. GO term analyses for Spongiotrophoblasts. (a-c) Spong_1, BP, MF, and CC ontology (d-f) Spong_2, BP, MF and CC ontology. GO term analyses are based on the hypergeometric test, with two-sided p-values adjusting for multiple comparisons via the Benjamini-Hochberg method to control the False Discovery Rate. Full GO analysis results for all cell types are available in Supplementary Figures 12 and 13.

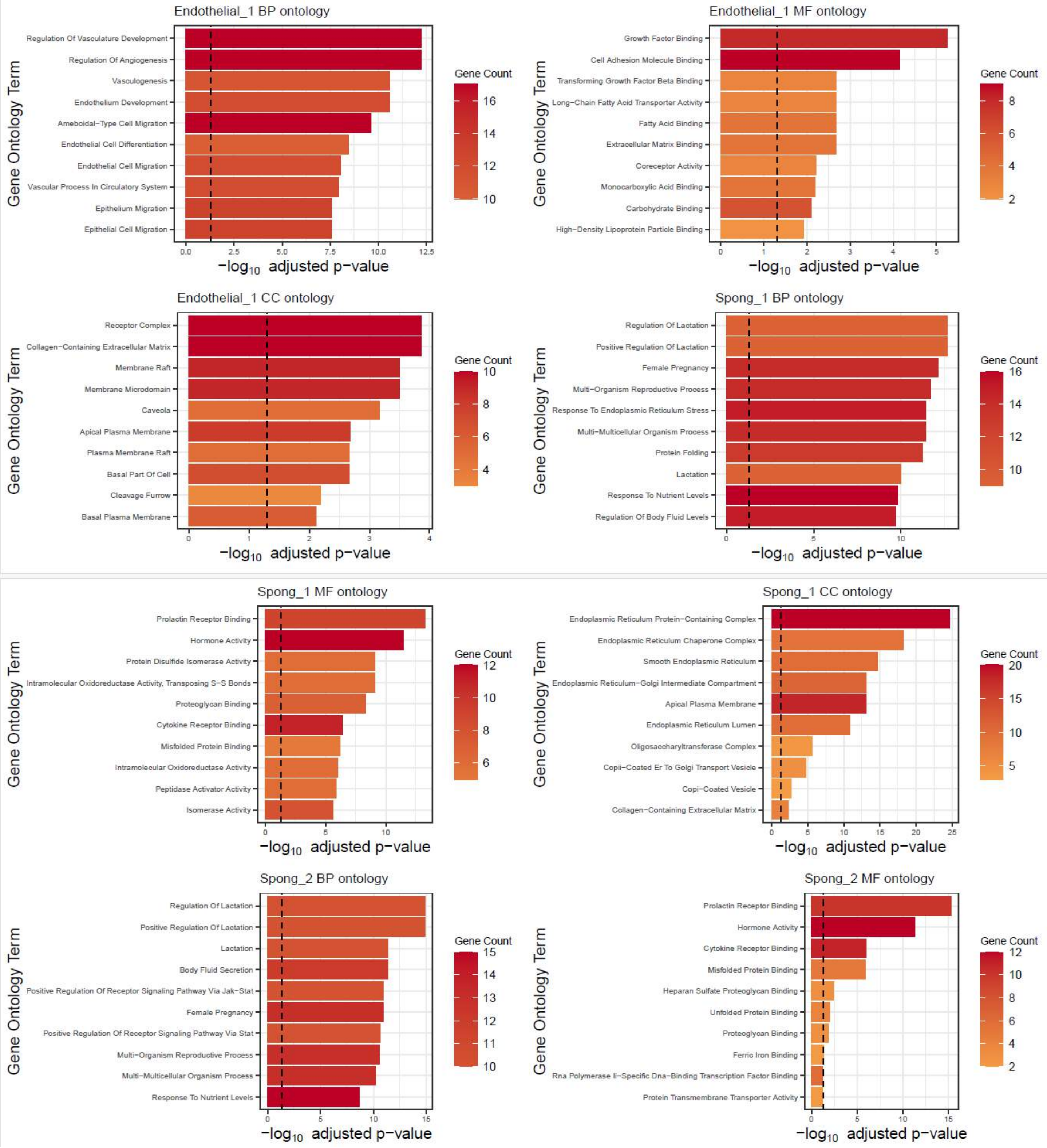

Endothelial_1 BP ontology
Regulation Of Vasculature Development
Regulation Of Angiogenesis
Vasculogenesis
Endothelium Development
Ameboidal-Type Cell Migration
Endothelial Cell Differentiation
Endothelial Cell Migration
Vascular Process In Circulatory System
Epithelium Migration
Epithelial Cell Migration
Gene Ontology Term
−log10 adjusted p−value
Gene Count
Endothelial_1 MF ontology
Growth Factor Binding
Cell Adhesion Molecule Binding
Transforming Growth Factor Beta Binding
Long-Chain Fatty Acid Transporter Activity
Fatty Acid Binding
Extracellular Matrix Binding
Coreceptor Activity
Monocarboxylic Acid Binding
Carbohydrate Binding
High-Density Lipoprotein Particle Binding
Endothelial_1 CC ontology
Receptor Complex
Collagen-Containing Extracellular Matrix
Membrane Raft
Membrane Microdomain
Caveola
Apical Plasma Membrane
Plasma Membrane Raft
Basal Part Of Cell
Cleavage Furrow
Basal Plasma Membrane
Spong_1 BP ontology
Regulation Of Lactation
Positive Regulation Of Lactation
Female Pregnancy
Multi-Organism Reproductive Process
Response To Endoplasmic Reticulum Stress
Multi-Multicellular Organism Process
Protein Folding
Lactation
Response To Nutrient Levels
Regulation Of Body Fluid Levels
Spong_1 MF ontology
Prolactin Receptor Binding
Hormone Activity
Protein Disulfide Isomerase Activity
Intramolecular Oxidoreductase Activity, Transposing S-S Bonds
Proteoglycan Binding
Cytokine Receptor Binding
Misfolded Protein Binding
Intramolecular Oxidoreductase Activity
Peptidase Activator Activity
Isomerase Activity
Spong_1 CC ontology
Endoplasmic Reticulum Protein-Containing Complex
Endoplasmic Reticulum Chaperone Complex
Smooth Endoplasmic Reticulum
Endoplasmic Reticulum-Golgi Intermediate Compartment
Apical Plasma Membrane
Endoplasmic Reticulum Lumen
Oligosaccharyltransferase Complex
Copii-Coated Er To Golgi Transport Vesicle
Copi-Coated Vesicle
Collagen-Containing Extracellular Matrix
Spong_2 BP ontology
Regulation Of Lactation
Positive Regulation Of Lactation
Lactation
Body Fluid Secretion
Positive Regulation Of Receptor Signaling Pathway Via Jak-Stat
Female Pregnancy
Positive Regulation Of Receptor Signaling Pathway Via Stat
Multi-Organism Reproductive Process
Multi-Multicellular Organism Process
Response To Nutrient Levels
Spong_2 MF ontology
Prolactin Receptor Binding
Hormone Activity
Cytokine Receptor Binding
Misfolded Protein Binding
Heparan Sulfate Proteoglycan Binding
Unfolded Protein Binding
Proteoglycan Binding
Ferric Iron Binding
Rna Polymerase Ii-Specific Dna-Binding Transcription Factor Binding
Protein Transmembrane Transporter Activity

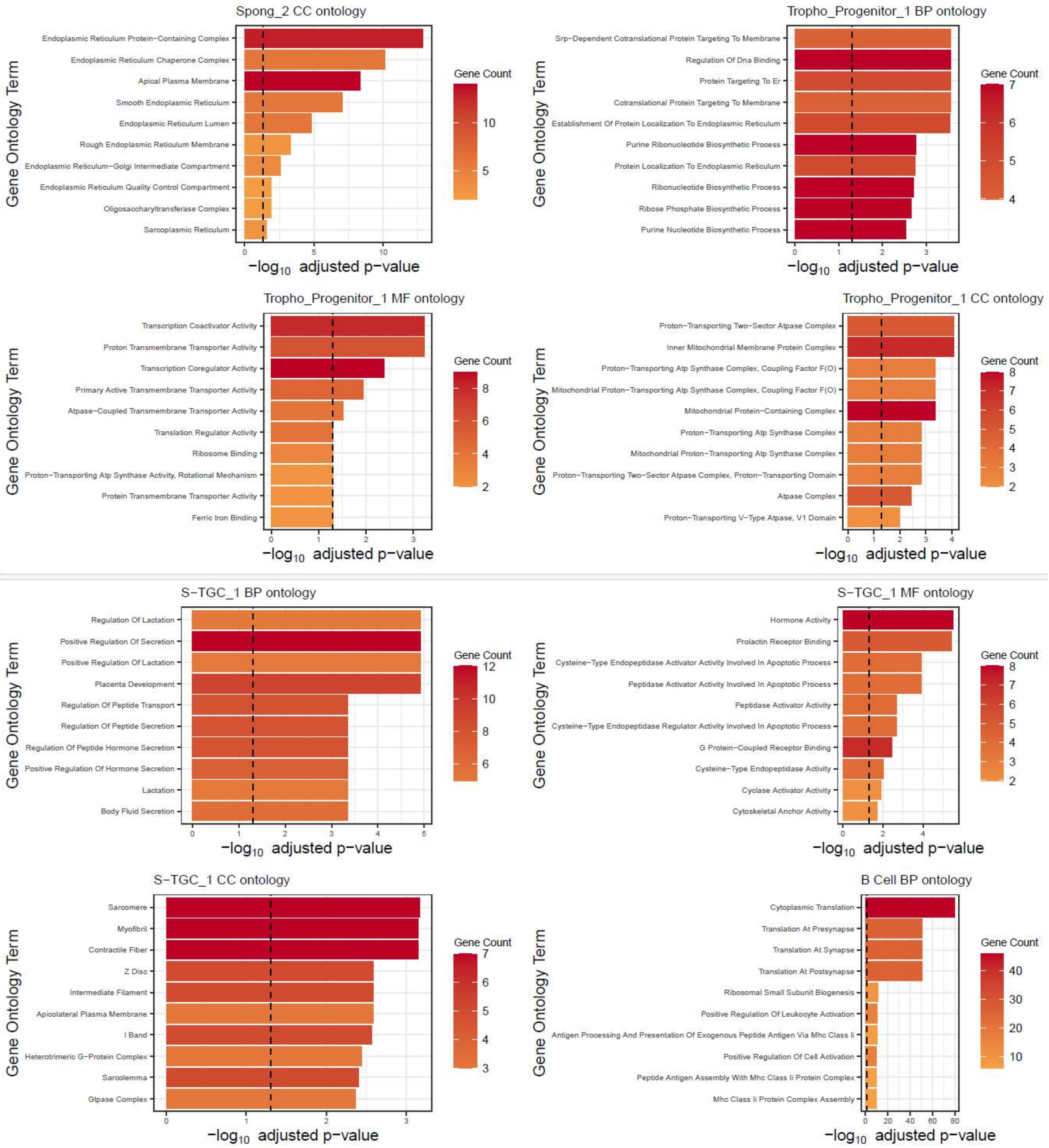
Spong_2 CC ontology
Endoplasmic Reticulum Protein−Containing Complex
Endoplasmic Reticulum Chaperone Complex
Apical Plasma Membrane
Smooth Endoplasmic Reticulum
Endoplasmic Reticulum Lumen
Rough Endoplasmic Reticulum Membrane
Endoplasmic Reticulum−Golgi Intermediate Compartment
Endoplasmic Reticulum Quality Control Compartment
Oligosaccharyltransferase Complex
Sarcoplasmic Reticulum
Gene Ontology Term
−log10 adjusted p−value
Gene Count
Tropho_Progenitor_1 BP ontology
Srp−Dependent Cotranslational Protein Targeting To Membrane
Regulation Of Dna Binding
Protein Targeting To Er
Cotranslational Protein Targeting To Membrane
Establishment Of Protein Localization To Endoplasmic Reticulum
Purine Ribonucleotide Biosynthetic Process
Protein Localization To Endoplasmic Reticulum
Ribonucleotide Biosynthetic Process
Ribose Phosphate Biosynthetic Process
Purine Nucleotide Biosynthetic Process
Tropho_Progenitor_1 MF ontology
Transcription Coactivator Activity
Proton Transmembrane Transporter Activity
Transcription Coregulator Activity
Primary Active Transmembrane Transporter Activity
Atpase−Coupled Transmembrane Transporter Activity
Translation Regulator Activity
Ribosome Binding
Proton−Transporting Atp Synthase Activity, Rotational Mechanism
Protein Transmembrane Transporter Activity
Ferric Iron Binding
Tropho_Progenitor_1 CC ontology
Proton−Transporting Two−Sector Atpase Complex
Inner Mitochondrial Membrane Protein Complex
Proton−Transporting Atp Synthase Complex, Coupling Factor F(O)
Mitochondrial Proton−Transporting Atp Synthase Complex, Coupling Factor F(O)
Mitochondrial Protein−Containing Complex
Proton−Transporting Atp Synthase Complex
Mitochondrial Proton−Transporting Atp Synthase Complex
Proton−Transporting Two−Sector Atpase Complex, Proton−Transporting Domain
Atpase Complex
Proton−Transporting V−Type Atpase, V1 Domain
S−TGC_1 BP ontology
Regulation Of Lactation
Positive Regulation Of Secretion
Positive Regulation Of Lactation
Placenta Development
Regulation Of Peptide Transport
Regulation Of Peptide Secretion
Regulation Of Peptide Hormone Secretion
Positive Regulation Of Hormone Secretion
Lactation
Body Fluid Secretion
S−TGC_1 MF ontology
Hormone Activity
Prolactin Receptor Binding
Cysteine−Type Endopeptidase Activator Activity Involved In Apoptotic Process
Peptidase Activator Activity Involved In Apoptotic Process
Peptidase Activator Activity
Cysteine−Type Endopeptidase Regulator Activity Involved In Apoptotic Process
G Protein−Coupled Receptor Binding
Cysteine−Type Endopeptidase Activity
Cyclase Activator Activity
Cytoskeletal Anchor Activity
S−TGC_1 CC ontology
Sarcomere
Myofibril
Contractile Fiber
Z Disc
Intermediate Filament
Apicolateral Plasma Membrane
I Band
Heterotrimeric G−Protein Complex
Sarcolemma
Gtpase Complex
B Cell BP ontology
Cytoplasmic Translation
Translation At Presynapse
Translation At Synapse
Translation At Postsynapse
Ribosomal Small Subunit Biogenesis
Positive Regulation Of Leukocyte Activation
Antigen Processing And Presentation Of Exogenous Peptide Antigen Via Mhc Class Ii
Positive Regulation Of Cell Activation
Peptide Antigen Assembly With Mhc Class Ii Protein Complex
Mhc Class Ii Protein Complex Assembly

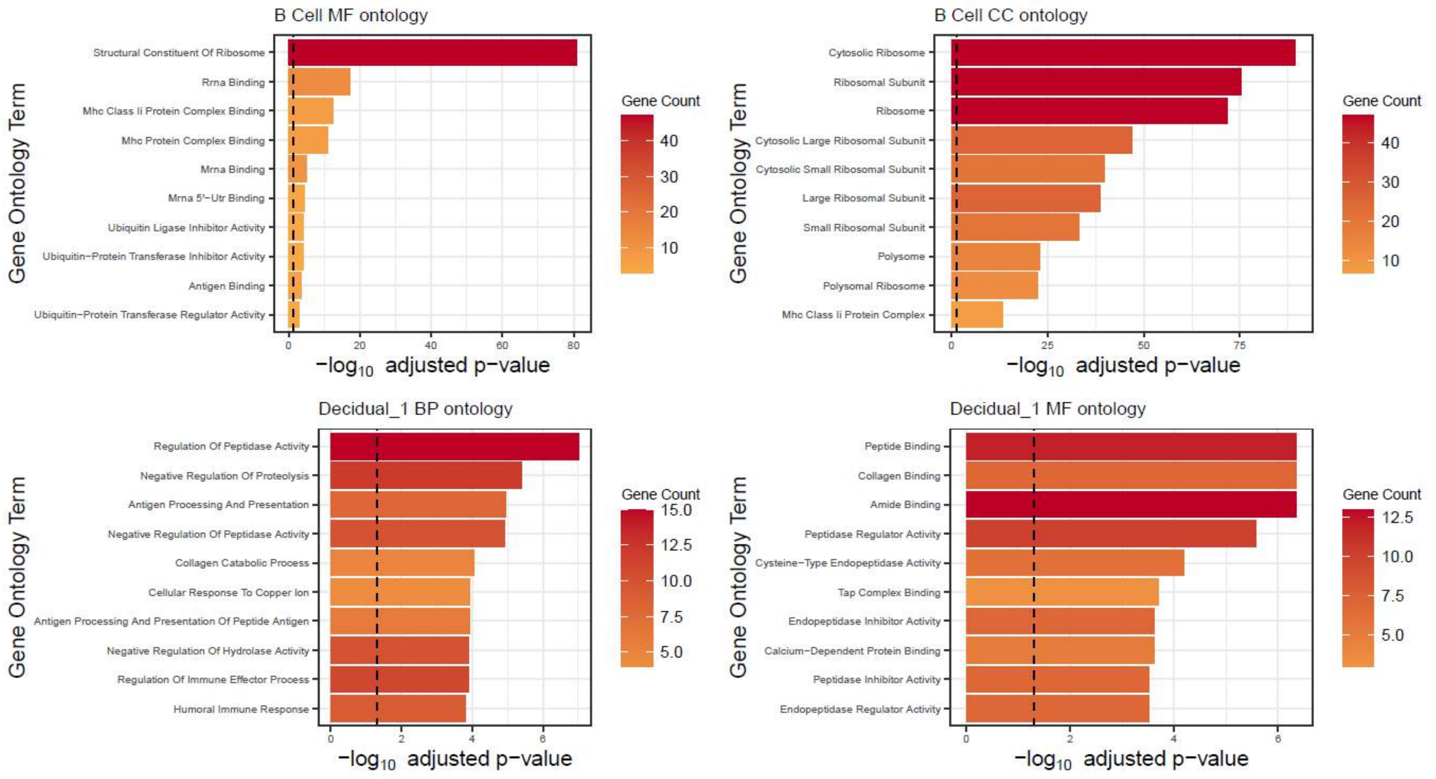
B Cell MF ontology
Structural Constituent Of Ribosome
Rrna Binding
Mhc Class Ii Protein Complex Binding
Mhc Protein Complex Binding
Mrna Binding
Mrna 5'−Utr Binding
Ubiquitin Ligase Inhibitor Activity
Ubiquitin−Protein Transferase Inhibitor Activity
Antigen Binding
Ubiquitin−Protein Transferase Regulator Activity
Gene Ontology Term
−log10 adjusted p−value
Gene Count
B Cell CC ontology
Cytosolic Ribosome
Ribosomal Subunit
Ribosome
Cytosolic Large Ribosomal Subunit
Cytosolic Small Ribosomal Subunit
Large Ribosomal Subunit
Small Ribosomal Subunit
Polysome
Polysomal Ribosome
Mhc Class Ii Protein Complex
Decidual_1 BP ontology
Regulation Of Peptidase Activity
Negative Regulation Of Proteolysis
Antigen Processing And Presentation
Negative Regulation Of Peptidase Activity
Collagen Catabolic Process
Cellular Response To Copper Ion
Antigen Processing And Presentation Of Peptide Antigen
Negative Regulation Of Hydrolase Activity
Regulation Of Immune Effector Process
Humoral Immune Response
Decidual_1 MF ontology
Peptide Binding
Collagen Binding
Amide Binding
Peptidase Regulator Activity
Cysteine−Type Endopeptidase Activity
Tap Complex Binding
Endopeptidase Inhibitor Activity
Calcium−Dependent Protein Binding
Peptidase Inhibitor Activity
Endopeptidase Regulator Activity

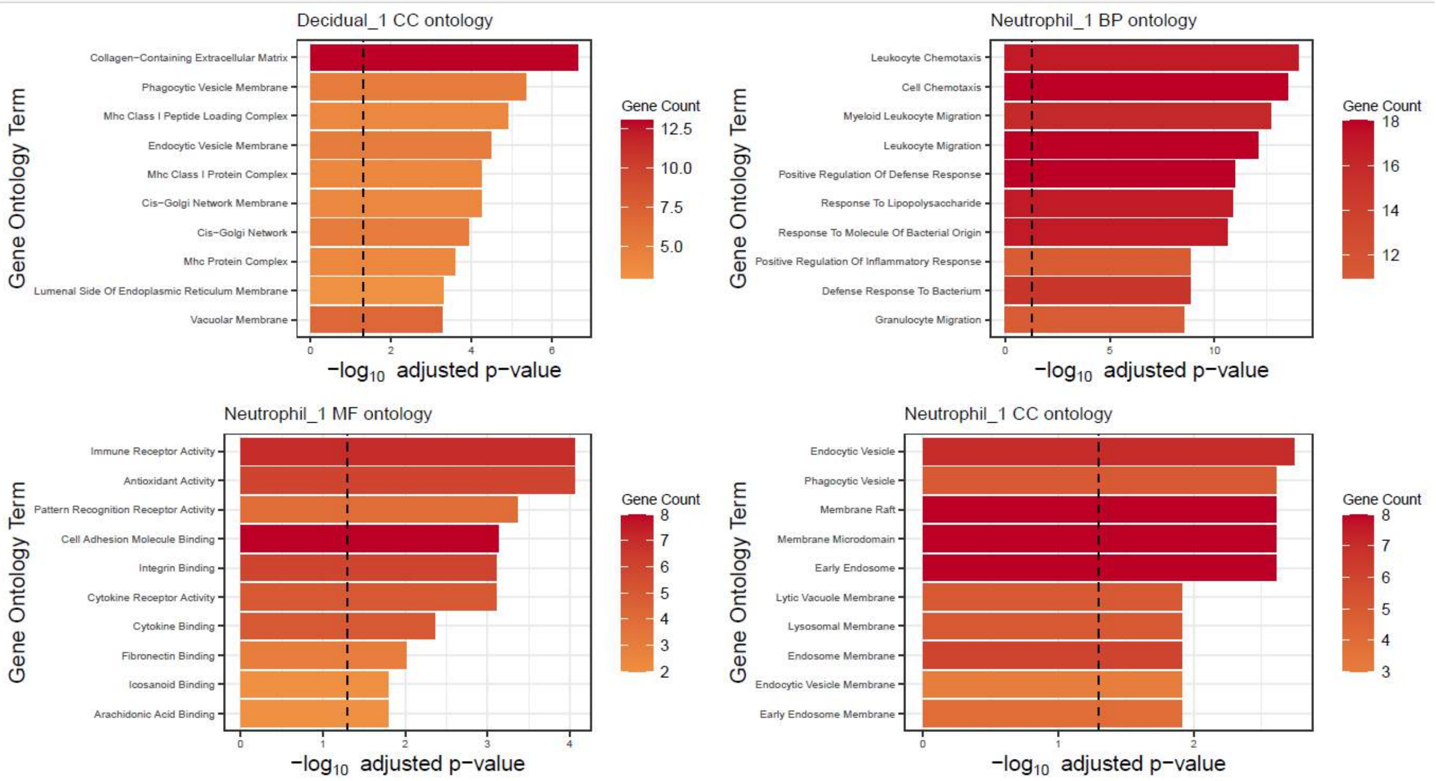
Decidual_1 CC ontology
Collagen−Containing Extracellular Matrix
Phagocytic Vesicle Membrane
Mhc Class I Peptide Loading Complex
Endocytic Vesicle Membrane
Mhc Class I Protein Complex
Cis−Golgi Network Membrane
Cis−Golgi Network
Mhc Protein Complex
Lumenal Side Of Endoplasmic Reticulum Membrane
Vacuolar Membrane
Gene Ontology Term
−log10 adjusted p−value
Gene Count
Neutrophil_1 BP ontology
Leukocyte Chemotaxis
Cell Chemotaxis
Myeloid Leukocyte Migration
Leukocyte Migration
Positive Regulation Of Defense Response
Response To Lipopolysaccharide
Response To Molecule Of Bacterial Origin
Positive Regulation Of Inflammatory Response
Defense Response To Bacterium
Granulocyte Migration
Neutrophil_1 MF ontology
Immune Receptor Activity
Antioxidant Activity
Pattern Recognition Receptor Activity
Cell Adhesion Molecule Binding
Integrin Binding
Cytokine Receptor Activity
Cytokine Binding
Fibronectin Binding
Icosanoid Binding
Arachidonic Acid Binding
Neutrophil_1 CC ontology
Endocytic Vesicle
Phagocytic Vesicle
Membrane Raft
Membrane Microdomain
Early Endosome
Lytic Vacuole Membrane
Lysosomal Membrane
Endosome Membrane
Endocytic Vesicle Membrane
Early Endosome Membrane

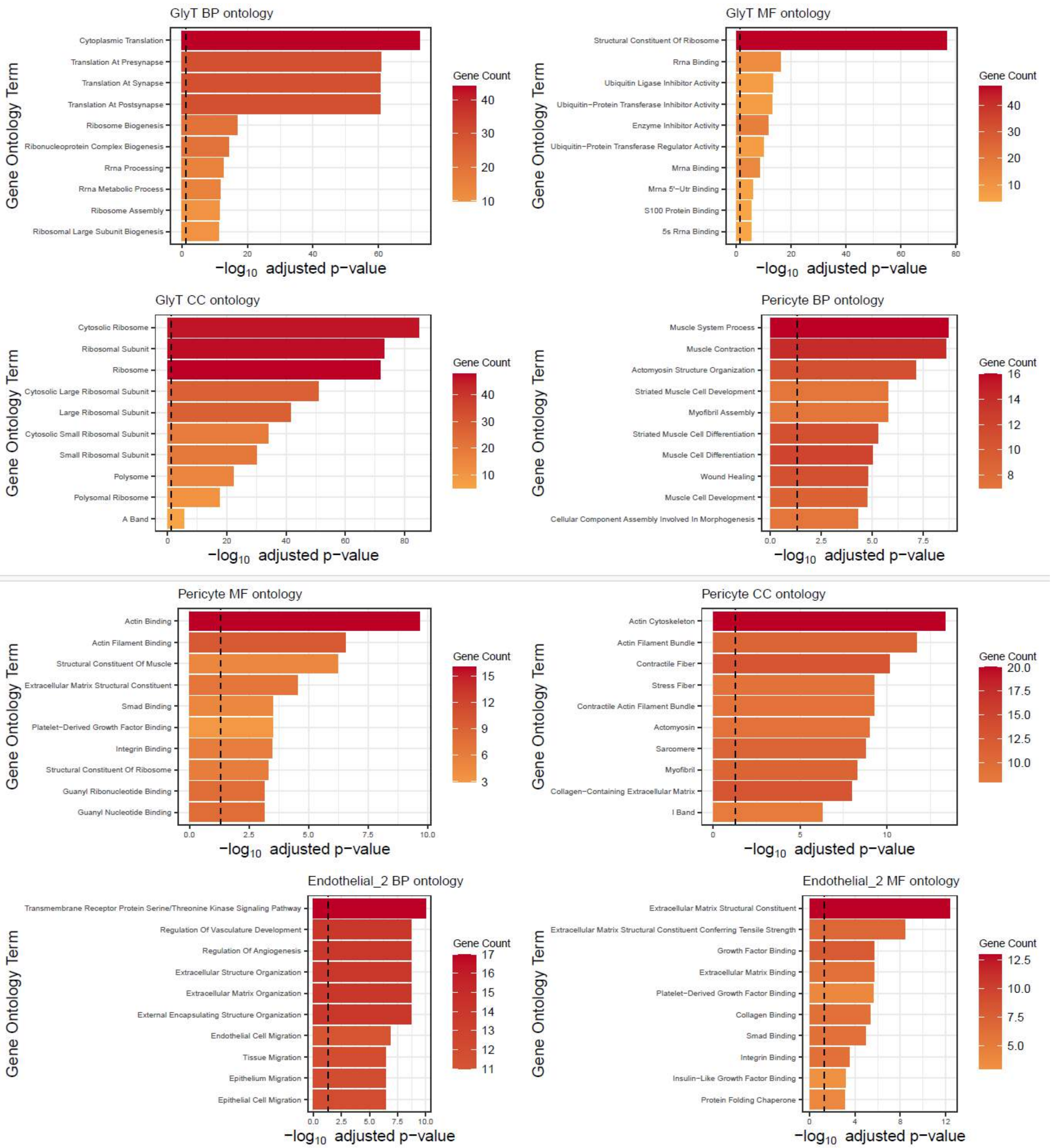
GlyT BP ontology
Cytoplasmic Translation
Translation At Presynapse
Translation At Synapse
Translation At Postsynapse
Ribosome Biogenesis
Ribonucleoprotein Complex Biogenesis
Rrna Processing
Rrna Metabolic Process
Ribosome Assembly
Ribosomal Large Subunit Biogenesis
GlyT MF ontology
Structural Constituent Of Ribosome
Rrna Binding
Ubiquitin Ligase Inhibitor Activity
Ubiquitin−Protein Transferase Inhibitor Activity
Enzyme Inhibitor Activity
Ubiquitin−Protein Transferase Regulator Activity
Mrna Binding
Mrna 5'−Utr Binding
S100 Protein Binding
5s Rrna Binding
GlyT CC ontology
Cytosolic Ribosome
Ribosomal Subunit
Ribosome
Cytosolic Large Ribosomal Subunit
Large Ribosomal Subunit
Cytosolic Small Ribosomal Subunit
Small Ribosomal Subunit
Polysome
Polysomal Ribosome
A Band
Pericyte BP ontology
Muscle System Process
Muscle Contraction
Actomyosin Structure Organization
Striated Muscle Cell Development
Myofibril Assembly
Striated Muscle Cell Differentiation
Muscle Cell Differentiation
Wound Healing
Muscle Cell Development
Cellular Component Assembly Involved In Morphogenesis
Pericyte MF ontology
Actin Binding
Actin Filament Binding
Structural Constituent Of Muscle
Extracellular Matrix Structural Constituent
Smad Binding
Platelet−Derived Growth Factor Binding
Integrin Binding
Structural Constituent Of Ribosome
Guanyl Ribonucleotide Binding
Guanyl Nucleotide Binding
Pericyte CC ontology
Actin Cytoskeleton
Actin Filament Bundle
Contractile Fiber
Stress Fiber
Contractile Actin Filament Bundle
Actomyosin
Sarcomere
Myofibril
Collagen−Containing Extracellular Matrix
I Band
Endothelial_2 BP ontology
Transmembrane Receptor Protein Serine/Threonine Kinase Signaling Pathway
Regulation Of Vasculature Development
Regulation Of Angiogenesis
Extracellular Structure Organization
Extracellular Matrix Organization
External Encapsulating Structure Organization
Endothelial Cell Migration
Tissue Migration
Epithelium Migration
Epithelial Cell Migration
Endothelial_2 MF ontology
Extracellular Matrix Structural Constituent
Extracellular Matrix Structural Constituent Conferring Tensile Strength
Growth Factor Binding
Extracellular Matrix Binding
Platelet−Derived Growth Factor Binding
Collagen Binding
Smad Binding
Integrin Binding
Insulin−Like Growth Factor Binding
Protein Folding Chaperone
Gene Ontology Term
Gene Count
−log10 adjusted p−value

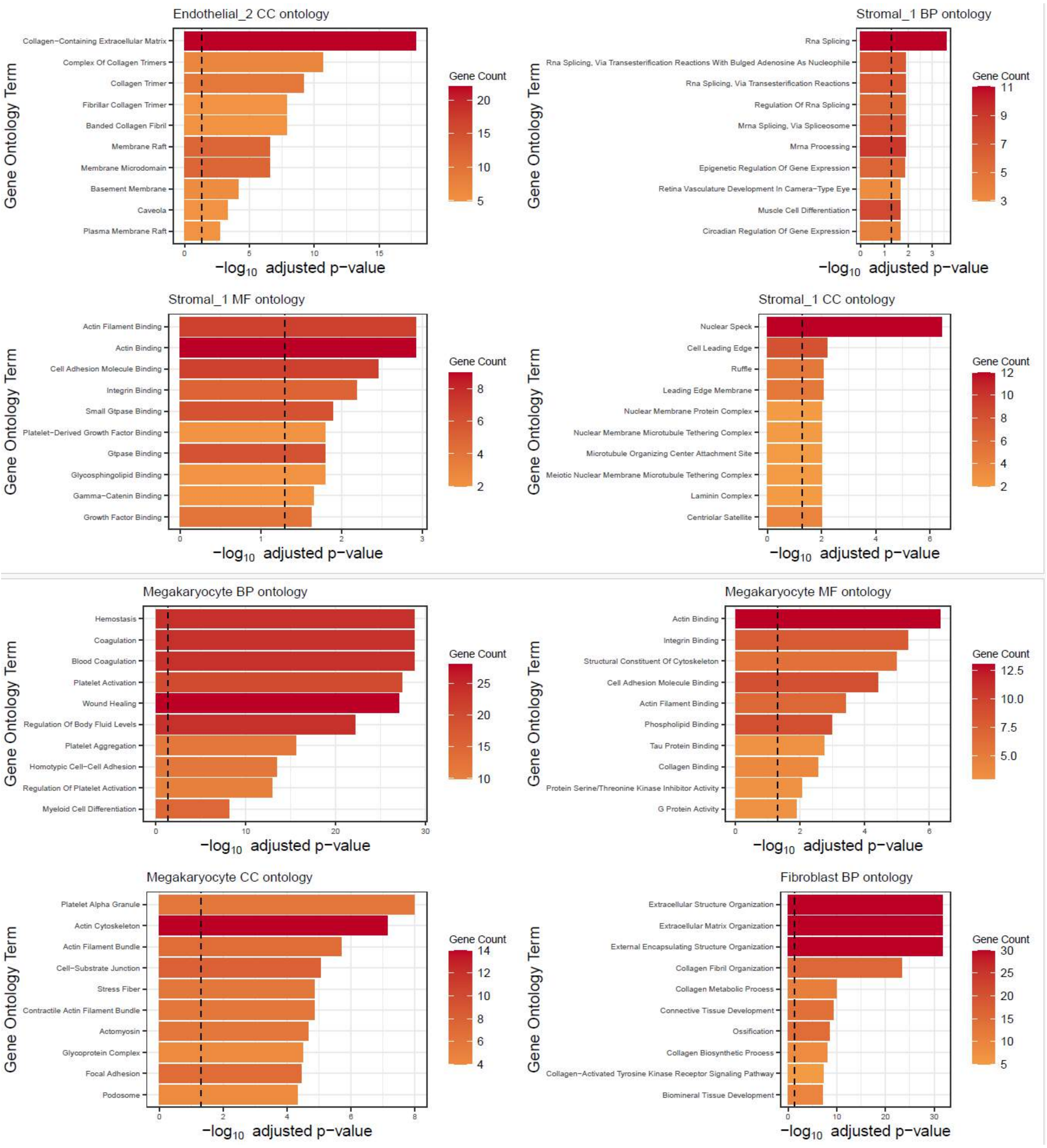

Endothelial_2 CC ontology
Collagen−Containing Extracellular Matrix
Complex Of Collagen Trimers
Collagen Trimer
Fibrillar Collagen Trimer
Banded Collagen Fibril
Membrane Raft
Membrane Microdomain
Basement Membrane
Caveola
Plasma Membrane Raft
Gene Ontology Term
−log10 adjusted p−value
Gene Count
Stromal_1 BP ontology
Rna Splicing
Rna Splicing, Via Transesterification Reactions With Bulged Adenosine As Nucleophile
Rna Splicing, Via Transesterification Reactions
Regulation Of Rna Splicing
Mrna Splicing, Via Spliceosome
Mrna Processing
Epigenetic Regulation Of Gene Expression
Retina Vasculature Development In Camera−Type Eye
Muscle Cell Differentiation
Circadian Regulation Of Gene Expression
Stromal_1 MF ontology
Actin Filament Binding
Actin Binding
Cell Adhesion Molecule Binding
Integrin Binding
Small Gtpase Binding
Platelet−Derived Growth Factor Binding
Gtpase Binding
Glycosphingolipid Binding
Gamma−Catenin Binding
Growth Factor Binding
Stromal_1 CC ontology
Nuclear Speck
Cell Leading Edge
Ruffle
Leading Edge Membrane
Nuclear Membrane Protein Complex
Nuclear Membrane Microtubule Tethering Complex
Microtubule Organizing Center Attachment Site
Meiotic Nuclear Membrane Microtubule Tethering Complex
Laminin Complex
Centriolar Satellite
Megakaryocyte BP ontology
Hemostasis
Coagulation
Blood Coagulation
Platelet Activation
Wound Healing
Regulation Of Body Fluid Levels
Platelet Aggregation
Homotypic Cell−Cell Adhesion
Regulation Of Platelet Activation
Myeloid Cell Differentiation
Megakaryocyte MF ontology
Actin Binding
Integrin Binding
Structural Constituent Of Cytoskeleton
Cell Adhesion Molecule Binding
Actin Filament Binding
Phospholipid Binding
Tau Protein Binding
Collagen Binding
Protein Serine/Threonine Kinase Inhibitor Activity
G Protein Activity
Megakaryocyte CC ontology
Platelet Alpha Granule
Actin Cytoskeleton
Actin Filament Bundle
Cell−Substrate Junction
Stress Fiber
Contractile Actin Filament Bundle
Actomyosin
Glycoprotein Complex
Focal Adhesion
Podosome
Fibroblast BP ontology
Extracellular Structure Organization
Extracellular Matrix Organization
External Encapsulating Structure Organization
Collagen Fibril Organization
Collagen Metabolic Process
Connective Tissue Development
Ossification
Collagen Biosynthetic Process
Collagen−Activated Tyrosine Kinase Receptor Signaling Pathway
Biomineral Tissue Development

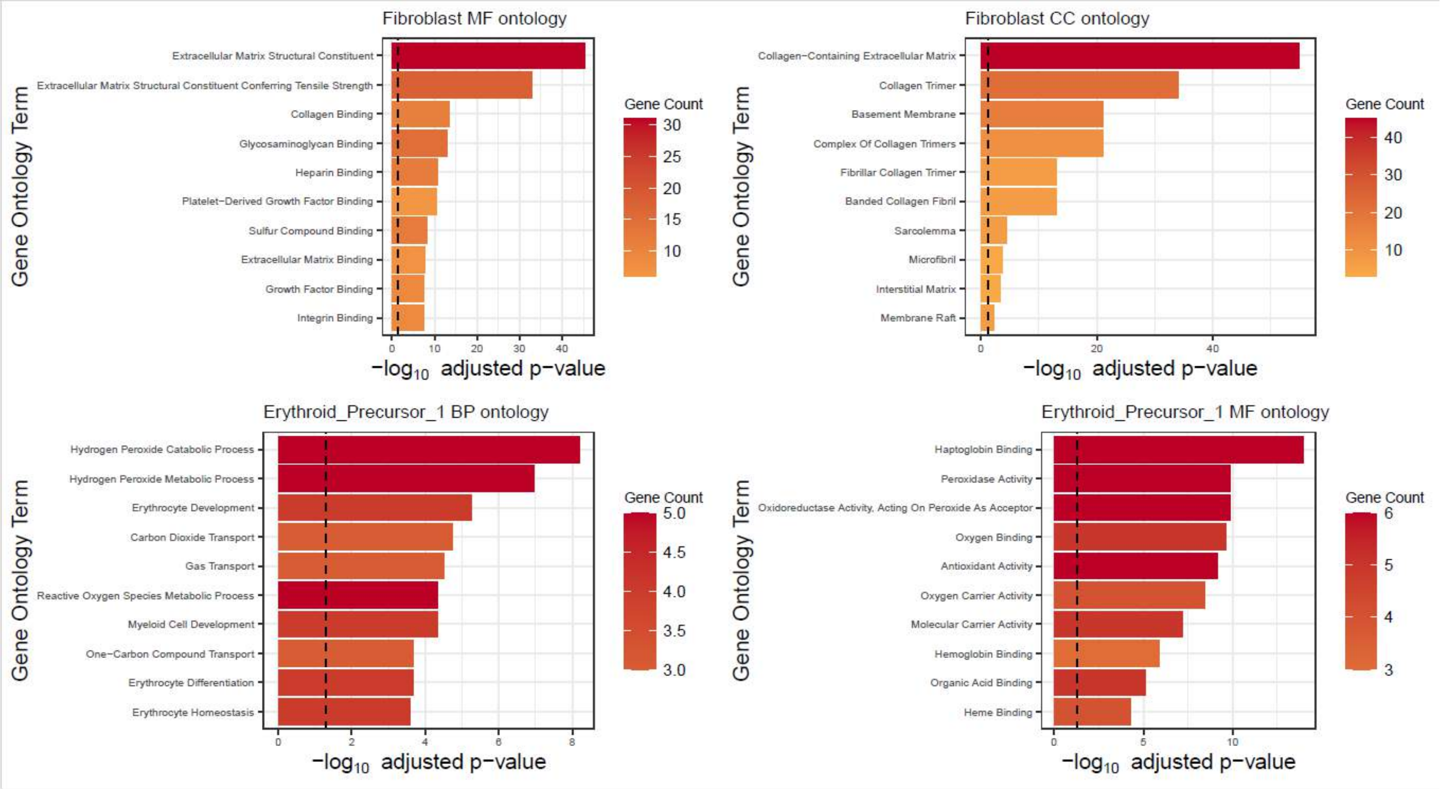

Fibroblast MF ontology
Extracellular Matrix Structural Constituent
Extracellular Matrix Structural Constituent Conferring Tensile Strength
Collagen Binding
Glycosaminoglycan Binding
Heparin Binding
Platelet−Derived Growth Factor Binding
Sulfur Compound Binding
Extracellular Matrix Binding
Growth Factor Binding
Integrin Binding
Gene Ontology Term
−log10 adjusted p−value
Gene Count
Fibroblast CC ontology
Collagen−Containing Extracellular Matrix
Collagen Trimer
Basement Membrane
Complex Of Collagen Trimers
Fibrillar Collagen Trimer
Banded Collagen Fibril
Sarcolemma
Microfibril
Interstitial Matrix
Membrane Raft
Erythroid_Precursor_1 BP ontology
Hydrogen Peroxide Catabolic Process
Hydrogen Peroxide Metabolic Process
Erythrocyte Development
Carbon Dioxide Transport
Gas Transport
Reactive Oxygen Species Metabolic Process
Myeloid Cell Development
One−Carbon Compound Transport
Erythrocyte Differentiation
Erythrocyte Homeostasis
Erythroid_Precursor_1 MF ontology
Haptoglobin Binding
Peroxidase Activity
Oxidoreductase Activity, Acting On Peroxide As Acceptor
Oxygen Binding
Antioxidant Activity
Oxygen Carrier Activity
Molecular Carrier Activity
Hemoglobin Binding
Organic Acid Binding
Heme Binding

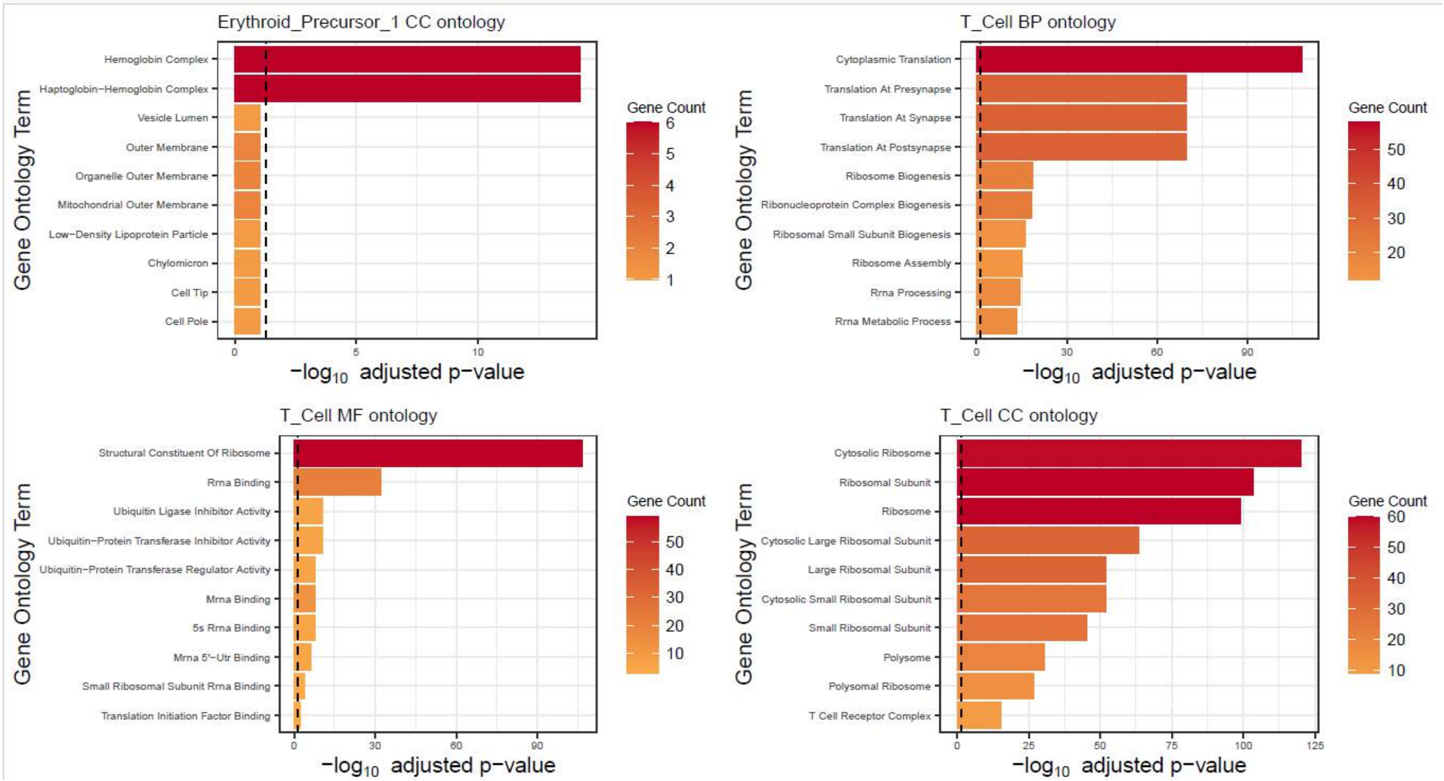

Erythroid_Precursor_1 CC ontology
Hemoglobin Complex
Haptoglobin−Hemoglobin Complex
Vesicle Lumen
Outer Membrane
Organelle Outer Membrane
Mitochondrial Outer Membrane
Low−Density Lipoprotein Particle
Chylomicron
Cell Tip
Cell Pole
Gene Ontology Term
−log10 adjusted p−value
Gene Count
T_Cell BP ontology
Cytoplasmic Translation
Translation At Presynapse
Translation At Synapse
Translation At Postsynapse
Ribosome Biogenesis
Ribonucleoprotein Complex Biogenesis
Ribosomal Small Subunit Biogenesis
Ribosome Assembly
Rrna Processing
Rrna Metabolic Process
T_Cell MF ontology
Structural Constituent Of Ribosome
Rrna Binding
Ubiquitin Ligase Inhibitor Activity
Ubiquitin−Protein Transferase Inhibitor Activity
Ubiquitin−Protein Transferase Regulator Activity
Mrna Binding
5s Rrna Binding
Mrna 5'−Utr Binding
Small Ribosomal Subunit Rrna Binding
Translation Initiation Factor Binding
T_Cell CC ontology
Cytosolic Ribosome
Ribosomal Subunit
Ribosome
Cytosolic Large Ribosomal Subunit
Large Ribosomal Subunit
Cytosolic Small Ribosomal Subunit
Small Ribosomal Subunit
Polysome
Polysomal Ribosome
T Cell Receptor Complex

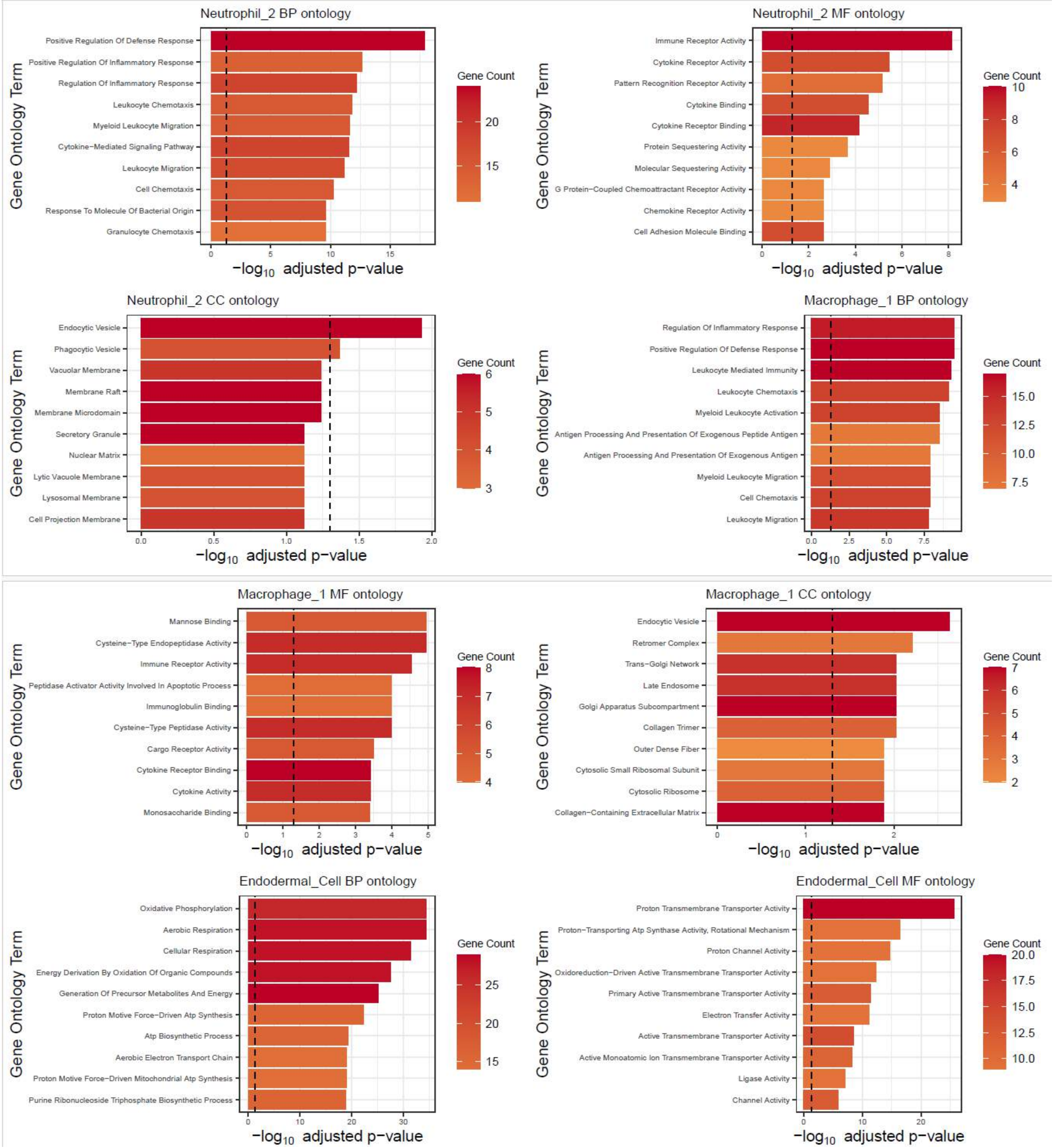
Neutrophil_2 BP ontology
Positive Regulation Of Defense Response
Positive Regulation Of Inflammatory Response
Regulation Of Inflammatory Response
Leukocyte Chemotaxis
Myeloid Leukocyte Migration
Cytokine−Mediated Signaling Pathway
Leukocyte Migration
Cell Chemotaxis
Response To Molecule Of Bacterial Origin
Granulocyte Chemotaxis
Gene Ontology Term
−log10 adjusted p−value
Gene Count
Neutrophil_2 MF ontology
Immune Receptor Activity
Cytokine Receptor Activity
Pattern Recognition Receptor Activity
Cytokine Binding
Cytokine Receptor Binding
Protein Sequestering Activity
Molecular Sequestering Activity
G Protein−Coupled Chemoattractant Receptor Activity
Chemokine Receptor Activity
Cell Adhesion Molecule Binding
Neutrophil_2 CC ontology
Endocytic Vesicle
Phagocytic Vesicle
Vacuolar Membrane
Membrane Raft
Membrane Microdomain
Secretory Granule
Nuclear Matrix
Lytic Vacuole Membrane
Lysosomal Membrane
Cell Projection Membrane
Macrophage_1 BP ontology
Regulation Of Inflammatory Response
Positive Regulation Of Defense Response
Leukocyte Mediated Immunity
Leukocyte Chemotaxis
Myeloid Leukocyte Activation
Antigen Processing And Presentation Of Exogenous Peptide Antigen
Antigen Processing And Presentation Of Exogenous Antigen
Myeloid Leukocyte Migration
Cell Chemotaxis
Leukocyte Migration
Macrophage_1 MF ontology
Mannose Binding
Cysteine−Type Endopeptidase Activity
Immune Receptor Activity
Peptidase Activator Activity Involved In Apoptotic Process
Immunoglobulin Binding
Cysteine−Type Peptidase Activity
Cargo Receptor Activity
Cytokine Receptor Binding
Cytokine Activity
Monosaccharide Binding
Macrophage_1 CC ontology
Endocytic Vesicle
Retromer Complex
Trans−Golgi Network
Late Endosome
Golgi Apparatus Subcompartment
Collagen Trimer
Outer Dense Fiber
Cytosolic Small Ribosomal Subunit
Cytosolic Ribosome
Collagen−Containing Extracellular Matrix
Endodermal_Cell BP ontology
Oxidative Phosphorylation
Aerobic Respiration
Cellular Respiration
Energy Derivation By Oxidation Of Organic Compounds
Generation Of Precursor Metabolites And Energy
Proton Motive Force−Driven Atp Synthesis
Atp Biosynthetic Process
Aerobic Electron Transport Chain
Proton Motive Force−Driven Mitochondrial Atp Synthesis
Purine Ribonucleoside Triphosphate Biosynthetic Process
Endodermal_Cell MF ontology
Proton Transmembrane Transporter Activity
Proton−Transporting Atp Synthase Activity, Rotational Mechanism
Proton Channel Activity
Oxidoreduction−Driven Active Transmembrane Transporter Activity
Primary Active Transmembrane Transporter Activity
Electron Transfer Activity
Active Transmembrane Transporter Activity
Active Monoatomic Ion Transmembrane Transporter Activity
Ligase Activity
Channel Activity

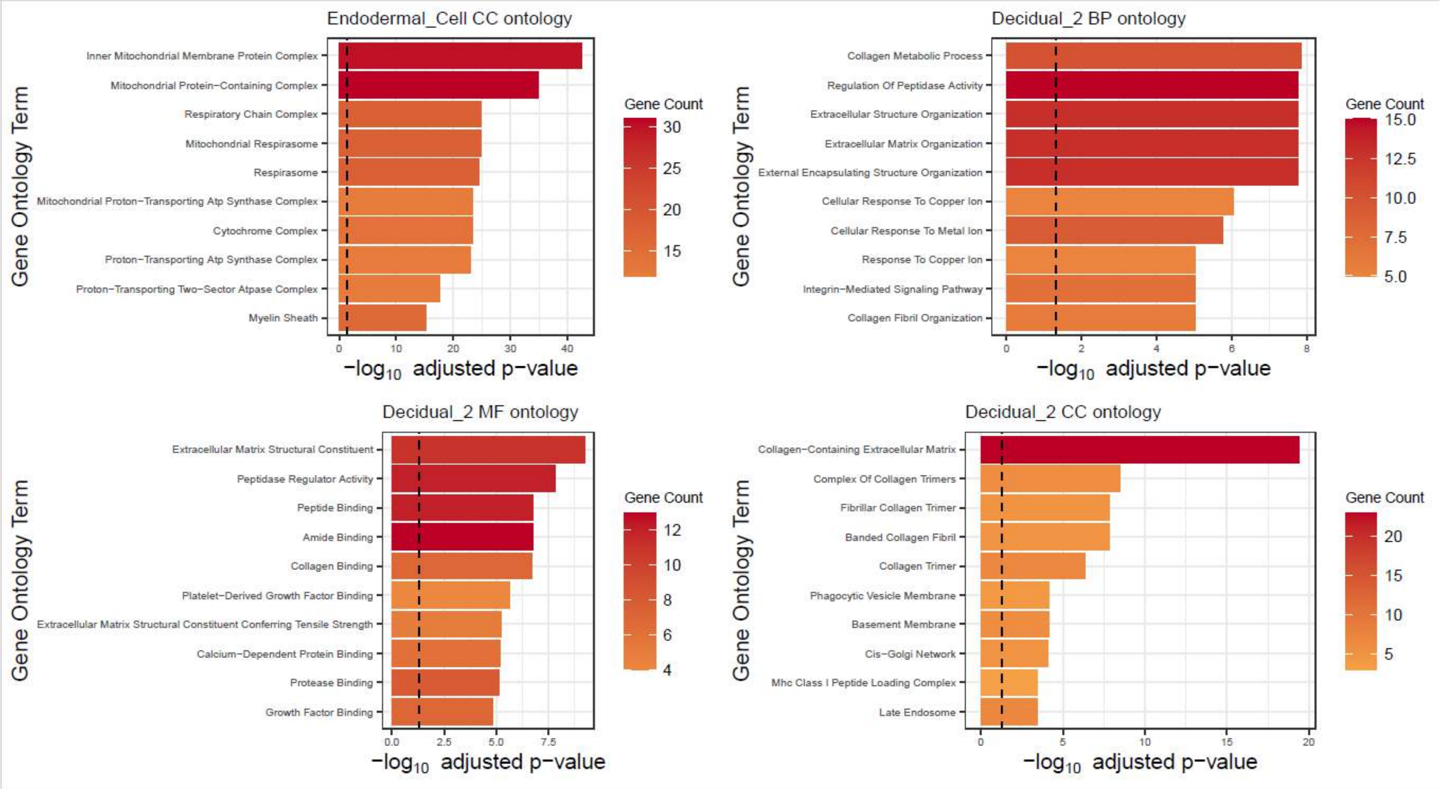
Endodermal_Cell CC ontology
Gene Ontology Term
Inner Mitochondrial Membrane Protein Complex
Mitochondrial Protein−Containing Complex
Respiratory Chain Complex
Mitochondrial Respirasome
Respirasome
Mitochondrial Proton−Transporting Atp Synthase Complex
Cytochrome Complex
Proton−Transporting Atp Synthase Complex
Proton−Transporting Two−Sector Atpase Complex
Myelin Sheath
0 10 20 30 40
−log10 adjusted p−value
Gene Count
30 25 20 15
Decidual_2 BP ontology
Gene Ontology Term
Collagen Metabolic Process
Regulation Of Peptidase Activity
Extracellular Structure Organization
Extracellular Matrix Organization
External Encapsulating Structure Organization
Cellular Response To Copper Ion
Cellular Response To Metal Ion
Response To Copper Ion
Integrin−Mediated Signaling Pathway
Collagen Fibril Organization
0 2 4 6 8
−log10 adjusted p−value
Gene Count
15.0 12.5 10.0 7.5 5.0
Decidual_2 MF ontology
Gene Ontology Term
Extracellular Matrix Structural Constituent
Peptidase Regulator Activity
Peptide Binding
Amide Binding
Collagen Binding
Platelet−Derived Growth Factor Binding
Extracellular Matrix Structural Constituent Conferring Tensile Strength
Calcium−Dependent Protein Binding
Protease Binding
Growth Factor Binding
0.0 2.5 5.0 7.5
−log10 adjusted p−value
Gene Count
12 10 8 6 4
Decidual_2 CC ontology
Gene Ontology Term
Collagen−Containing Extracellular Matrix
Complex Of Collagen Trimers
Fibrillar Collagen Trimer
Banded Collagen Fibril
Collagen Trimer
Phagocytic Vesicle Membrane
Basement Membrane
Cis−Golgi Network
Mhc Class I Peptide Loading Complex
Late Endosome
0 5 10 15 20
−log10 adjusted p−value
Gene Count
20 15 10 5

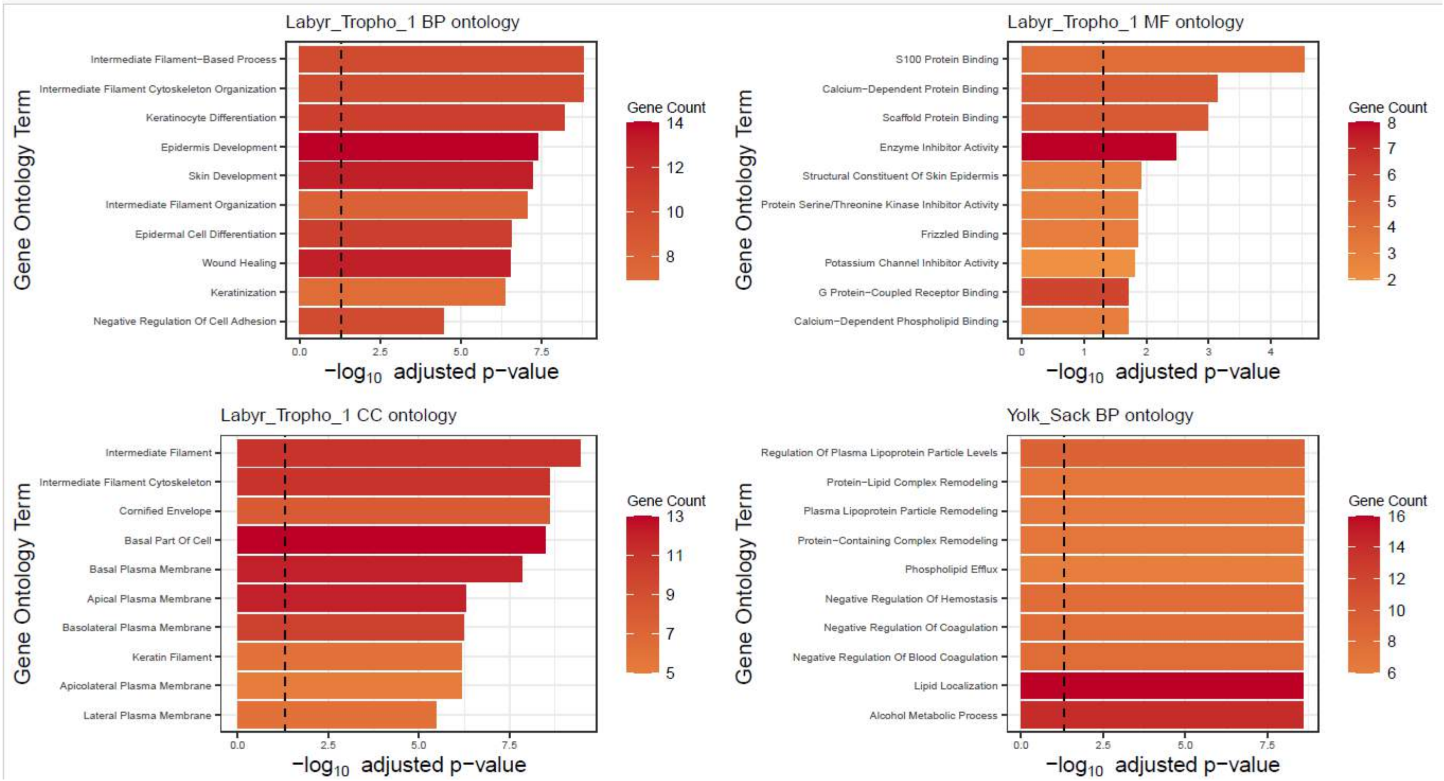
Labyr_Tropho_1 BP ontology
Gene Ontology Term
Intermediate Filament−Based Process
Intermediate Filament Cytoskeleton Organization
Keratinocyte Differentiation
Epidermis Development
Skin Development
Intermediate Filament Organization
Epidermal Cell Differentiation
Wound Healing
Keratinization
Negative Regulation Of Cell Adhesion
0.0 2.5 5.0 7.5
−log10 adjusted p−value
Gene Count
14 12 10 8
Labyr_Tropho_1 MF ontology
Gene Ontology Term
S100 Protein Binding
Calcium−Dependent Protein Binding
Scaffold Protein Binding
Enzyme Inhibitor Activity
Structural Constituent Of Skin Epidermis
Protein Serine/Threonine Kinase Inhibitor Activity
Frizzled Binding
Potassium Channel Inhibitor Activity
G Protein−Coupled Receptor Binding
Calcium−Dependent Phospholipid Binding
0 1 2 3 4
−log10 adjusted p−value
Gene Count
8 7 6 5 4 3 2
Labyr_Tropho_1 CC ontology
Gene Ontology Term
Intermediate Filament
Intermediate Filament Cytoskeleton
Cornified Envelope
Basal Part Of Cell
Basal Plasma Membrane
Apical Plasma Membrane
Basolateral Plasma Membrane
Keratin Filament
Apicolateral Plasma Membrane
Lateral Plasma Membrane
0.0 2.5 5.0 7.5
−log10 adjusted p−value
Gene Count
13 11 9 7 5
Yolk_Sack BP ontology
Gene Ontology Term
Regulation Of Plasma Lipoprotein Particle Levels
Protein−Lipid Complex Remodeling
Plasma Lipoprotein Particle Remodeling
Protein−Containing Complex Remodeling
Phospholipid Efflux
Negative Regulation Of Hemostasis
Negative Regulation Of Coagulation
Negative Regulation Of Blood Coagulation
Lipid Localization
Alcohol Metabolic Process
0.0 2.5 5.0 7.5
−log10 adjusted p−value
Gene Count
16 14 12 10 8 6

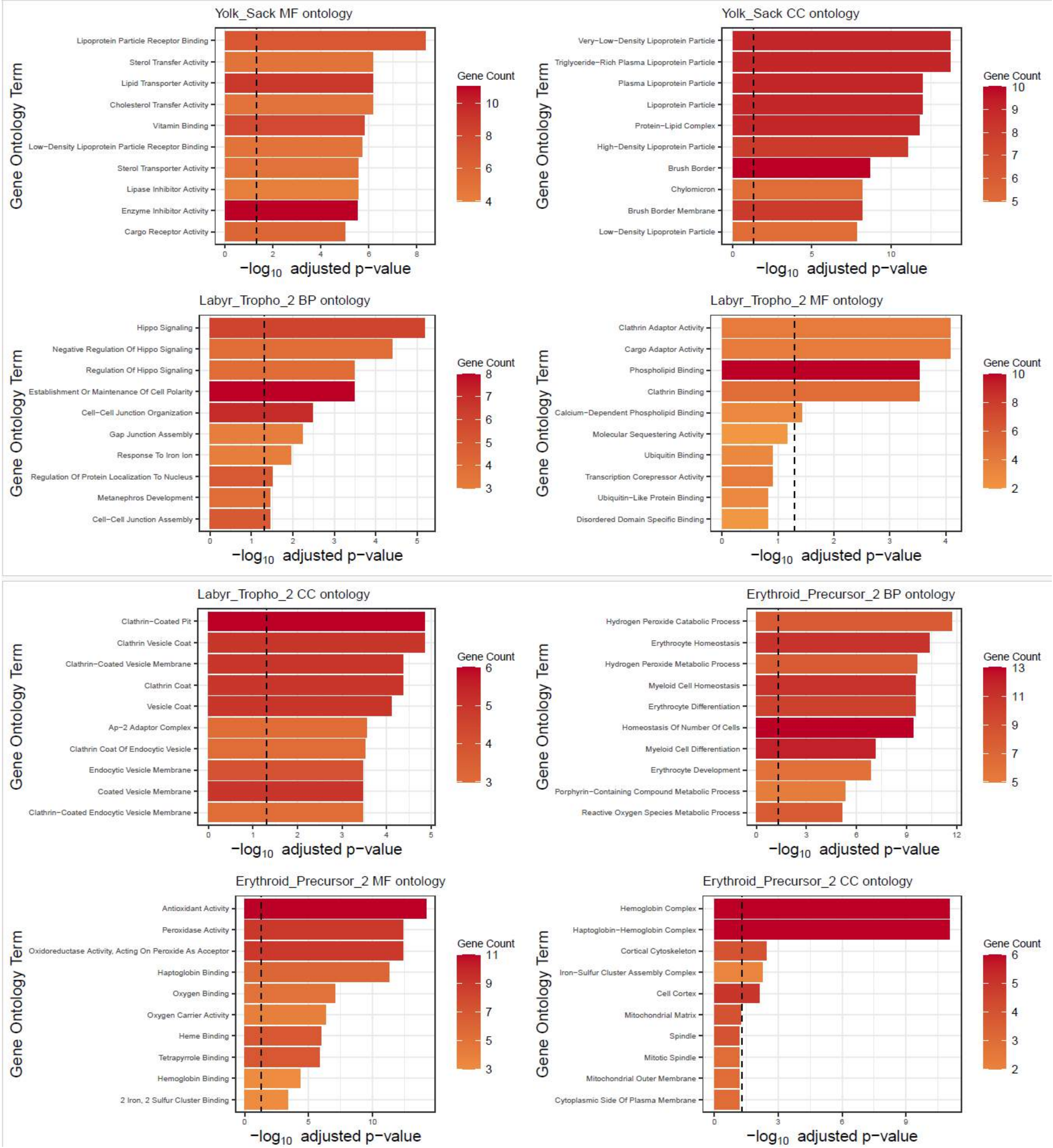

Yolk_Sack MF ontology
Lipoprotein Particle Receptor Binding
Sterol Transfer Activity
Lipid Transporter Activity
Cholesterol Transfer Activity
Vitamin Binding
Low−Density Lipoprotein Particle Receptor Binding
Sterol Transporter Activity
Lipase Inhibitor Activity
Enzyme Inhibitor Activity
Cargo Receptor Activity
Gene Ontology Term
−log10 adjusted p−value
Gene Count
Yolk_Sack CC ontology
Very−Low−Density Lipoprotein Particle
Triglyceride−Rich Plasma Lipoprotein Particle
Plasma Lipoprotein Particle
Lipoprotein Particle
Protein−Lipid Complex
High−Density Lipoprotein Particle
Brush Border
Chylomicron
Brush Border Membrane
Low−Density Lipoprotein Particle
Labyr_Tropho_2 BP ontology
Hippo Signaling
Negative Regulation Of Hippo Signaling
Regulation Of Hippo Signaling
Establishment Or Maintenance Of Cell Polarity
Cell−Cell Junction Organization
Gap Junction Assembly
Response To Iron Ion
Regulation Of Protein Localization To Nucleus
Metanephros Development
Cell−Cell Junction Assembly
Labyr_Tropho_2 MF ontology
Clathrin Adaptor Activity
Cargo Adaptor Activity
Phospholipid Binding
Clathrin Binding
Calcium−Dependent Phospholipid Binding
Molecular Sequestering Activity
Ubiquitin Binding
Transcription Corepressor Activity
Ubiquitin−Like Protein Binding
Disordered Domain Specific Binding
Labyr_Tropho_2 CC ontology
Clathrin−Coated Pit
Clathrin Vesicle Coat
Clathrin−Coated Vesicle Membrane
Clathrin Coat
Vesicle Coat
Ap−2 Adaptor Complex
Clathrin Coat Of Endocytic Vesicle
Endocytic Vesicle Membrane
Coated Vesicle Membrane
Clathrin−Coated Endocytic Vesicle Membrane
Erythroid_Precursor_2 BP ontology
Hydrogen Peroxide Catabolic Process
Erythrocyte Homeostasis
Hydrogen Peroxide Metabolic Process
Myeloid Cell Homeostasis
Erythrocyte Differentiation
Homeostasis Of Number Of Cells
Myeloid Cell Differentiation
Erythrocyte Development
Porphyrin−Containing Compound Metabolic Process
Reactive Oxygen Species Metabolic Process
Erythroid_Precursor_2 MF ontology
Antioxidant Activity
Peroxidase Activity
Oxidoreductase Activity, Acting On Peroxide As Acceptor
Haptoglobin Binding
Oxygen Binding
Oxygen Carrier Activity
Heme Binding
Tetrapyrrole Binding
Hemoglobin Binding
2 Iron, 2 Sulfur Cluster Binding
Erythroid_Precursor_2 CC ontology
Hemoglobin Complex
Haptoglobin−Hemoglobin Complex
Cortical Cytoskeleton
Iron−Sulfur Cluster Assembly Complex
Cell Cortex
Mitochondrial Matrix
Spindle
Mitotic Spindle
Mitochondrial Outer Membrane
Cytoplasmic Side Of Plasma Membrane

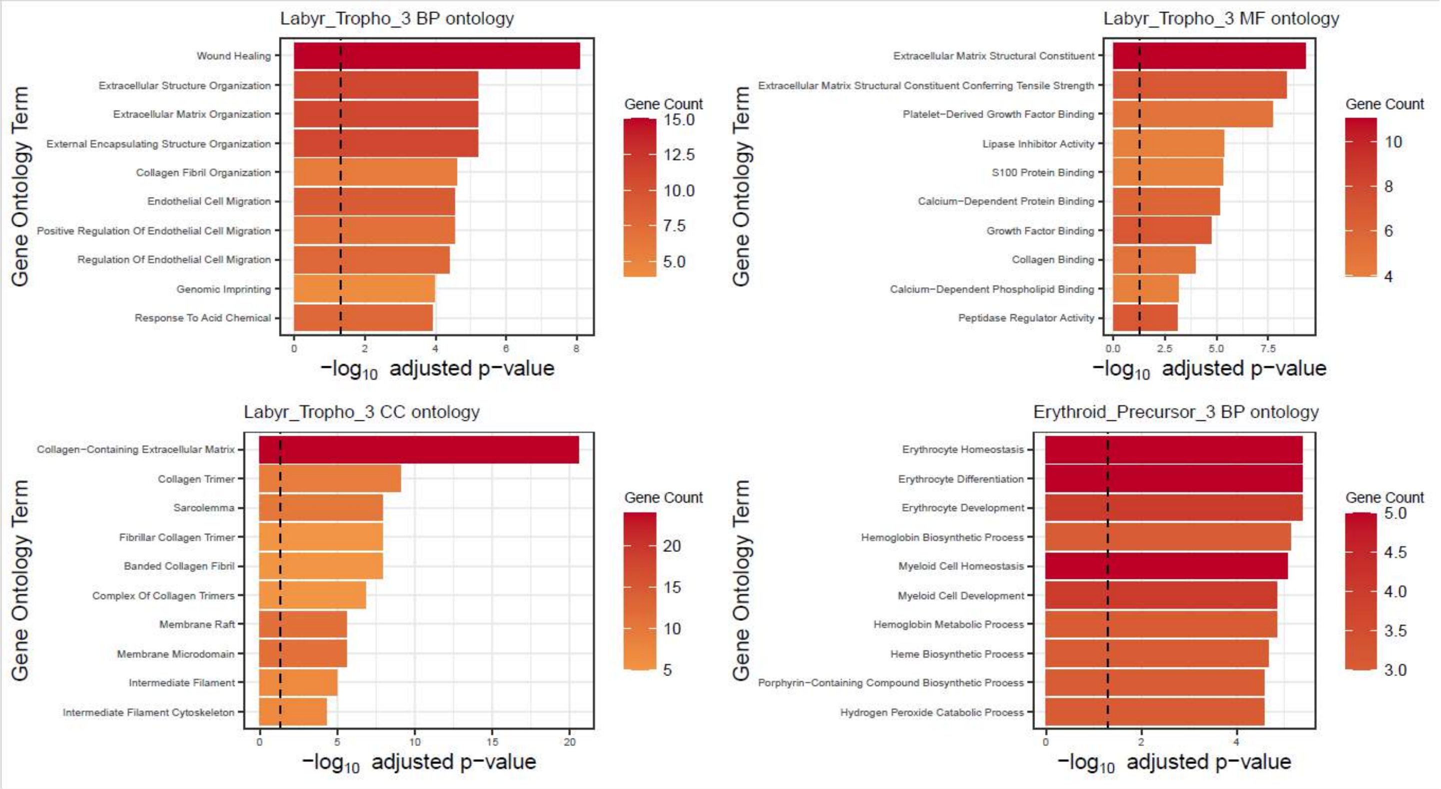
Labyr_Tropho_3 BP ontology
Wound Healing
Extracellular Structure Organization
Extracellular Matrix Organization
External Encapsulating Structure Organization
Collagen Fibril Organization
Endothelial Cell Migration
Positive Regulation Of Endothelial Cell Migration
Regulation Of Endothelial Cell Migration
Genomic Imprinting
Response To Acid Chemical
Gene Ontology Term
−log10 adjusted p−value
Gene Count
Labyr_Tropho_3 MF ontology
Extracellular Matrix Structural Constituent
Extracellular Matrix Structural Constituent Conferring Tensile Strength
Platelet−Derived Growth Factor Binding
Lipase Inhibitor Activity
S100 Protein Binding
Calcium−Dependent Protein Binding
Growth Factor Binding
Collagen Binding
Calcium−Dependent Phospholipid Binding
Peptidase Regulator Activity
Labyr_Tropho_3 CC ontology
Collagen−Containing Extracellular Matrix
Collagen Trimer
Sarcolemma
Fibrillar Collagen Trimer
Banded Collagen Fibril
Complex Of Collagen Trimers
Membrane Raft
Membrane Microdomain
Intermediate Filament
Intermediate Filament Cytoskeleton
Erythroid_Precursor_3 BP ontology
Erythrocyte Homeostasis
Erythrocyte Differentiation
Erythrocyte Development
Hemoglobin Biosynthetic Process
Myeloid Cell Homeostasis
Myeloid Cell Development
Hemoglobin Metabolic Process
Heme Biosynthetic Process
Porphyrin−Containing Compound Biosynthetic Process
Hydrogen Peroxide Catabolic Process

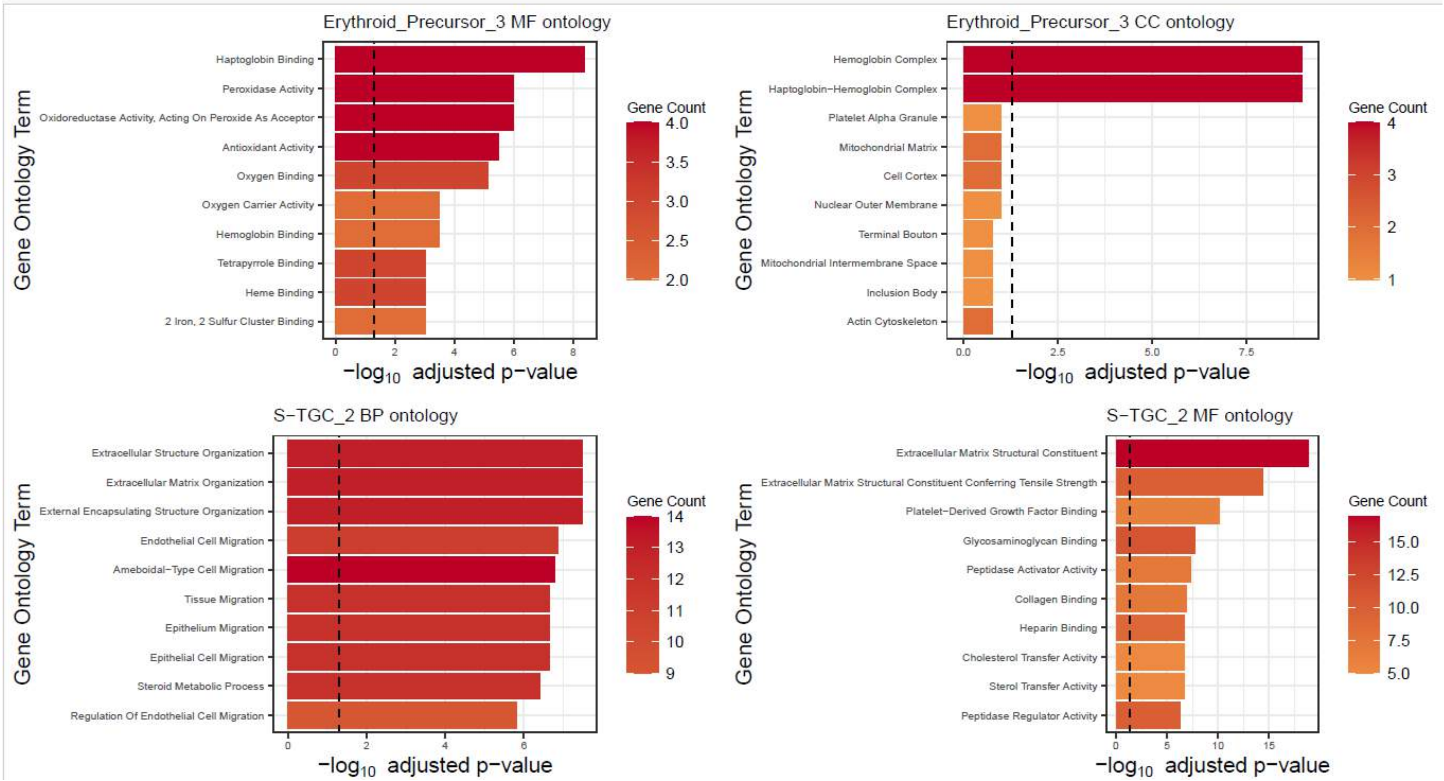
Erythroid_Precursor_3 MF ontology
Haptoglobin Binding
Peroxidase Activity
Oxidoreductase Activity, Acting On Peroxide As Acceptor
Antioxidant Activity
Oxygen Binding
Oxygen Carrier Activity
Hemoglobin Binding
Tetrapyrrole Binding
Heme Binding
2 Iron, 2 Sulfur Cluster Binding
Gene Ontology Term
−log10 adjusted p−value
Gene Count
Erythroid_Precursor_3 CC ontology
Hemoglobin Complex
Haptoglobin−Hemoglobin Complex
Platelet Alpha Granule
Mitochondrial Matrix
Cell Cortex
Nuclear Outer Membrane
Terminal Bouton
Mitochondrial Intermembrane Space
Inclusion Body
Actin Cytoskeleton
S−TGC_2 BP ontology
Extracellular Structure Organization
Extracellular Matrix Organization
External Encapsulating Structure Organization
Endothelial Cell Migration
Ameboidal−Type Cell Migration
Tissue Migration
Epithelium Migration
Epithelial Cell Migration
Steroid Metabolic Process
Regulation Of Endothelial Cell Migration
S−TGC_2 MF ontology
Extracellular Matrix Structural Constituent
Extracellular Matrix Structural Constituent Conferring Tensile Strength
Platelet−Derived Growth Factor Binding
Glycosaminoglycan Binding
Peptidase Activator Activity
Collagen Binding
Heparin Binding
Cholesterol Transfer Activity
Sterol Transfer Activity
Peptidase Regulator Activity

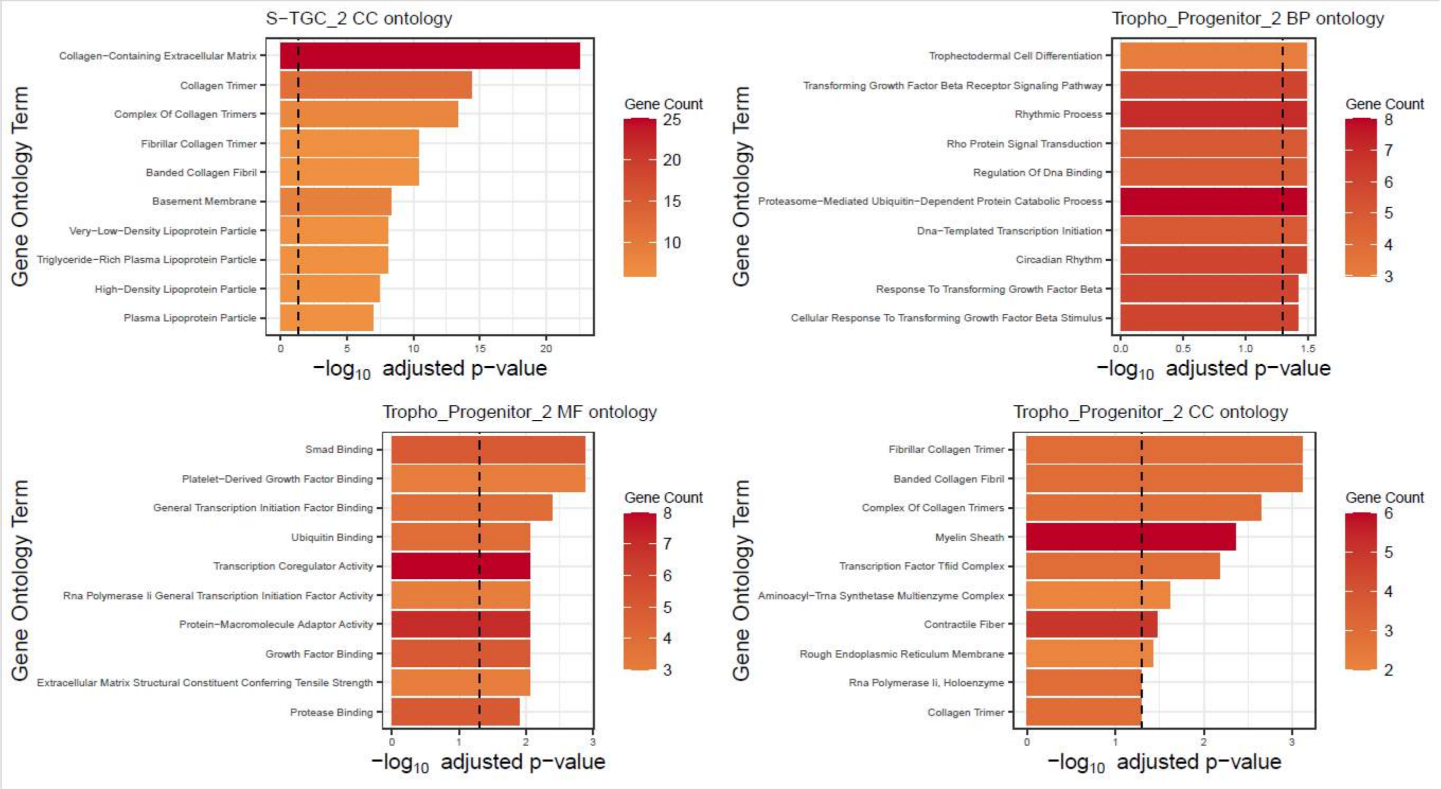
S−TGC_2 CC ontology
Gene Ontology Term
Collagen−Containing Extracellular Matrix
Collagen Trimer
Complex Of Collagen Trimers
Fibrillar Collagen Trimer
Banded Collagen Fibril
Basement Membrane
Very−Low−Density Lipoprotein Particle
Triglyceride−Rich Plasma Lipoprotein Particle
High−Density Lipoprotein Particle
Plasma Lipoprotein Particle
0 5 10 15 20
−log10 adjusted p−value
Gene Count
25 20 15 10
Tropho_Progenitor_2 BP ontology
Gene Ontology Term
Trophectodermal Cell Differentiation
Transforming Growth Factor Beta Receptor Signaling Pathway
Rhythmic Process
Rho Protein Signal Transduction
Regulation Of Dna Binding
Proteasome−Mediated Ubiquitin−Dependent Protein Catabolic Process
Dna−Templated Transcription Initiation
Circadian Rhythm
Response To Transforming Growth Factor Beta
Cellular Response To Transforming Growth Factor Beta Stimulus
0.0 0.5 1.0 1.5
−log10 adjusted p−value
Gene Count
8 7 6 5 4 3
Tropho_Progenitor_2 MF ontology
Gene Ontology Term
Smad Binding
Platelet−Derived Growth Factor Binding
General Transcription Initiation Factor Binding
Ubiquitin Binding
Transcription Coregulator Activity
Rna Polymerase Ii General Transcription Initiation Factor Activity
Protein−Macromolecule Adaptor Activity
Growth Factor Binding
Extracellular Matrix Structural Constituent Conferring Tensile Strength
Protease Binding
0 1 2 3
−log10 adjusted p−value
Gene Count
8 7 6 5 4 3
Tropho_Progenitor_2 CC ontology
Gene Ontology Term
Fibrillar Collagen Trimer
Banded Collagen Fibril
Complex Of Collagen Trimers
Myelin Sheath
Transcription Factor Tfiid Complex
Aminoacyl−Trna Synthetase Multienzyme Complex
Contractile Fiber
Rough Endoplasmic Reticulum Membrane
Rna Polymerase Ii, Holoenzyme
Collagen Trimer
0 1 2 3
−log10 adjusted p−value
Gene Count
6 5 4 3 2

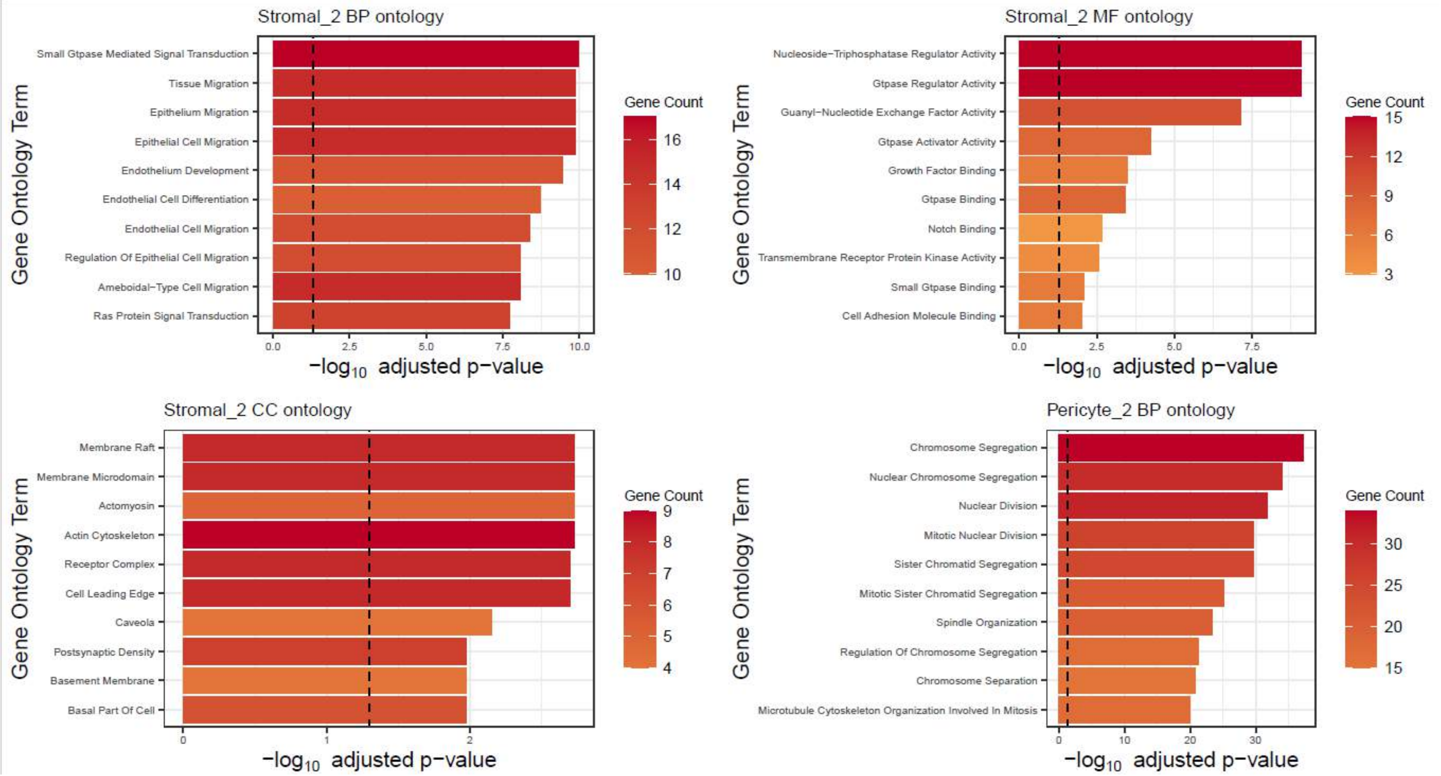
Stromal_2 BP ontology
Gene Ontology Term
Small Gtpase Mediated Signal Transduction
Tissue Migration
Epithelium Migration
Epithelial Cell Migration
Endothelium Development
Endothelial Cell Differentiation
Endothelial Cell Migration
Regulation Of Epithelial Cell Migration
Ameboidal−Type Cell Migration
Ras Protein Signal Transduction
0.0 2.5 5.0 7.5 10.0
−log10 adjusted p−value
Gene Count
16 14 12 10
Stromal_2 MF ontology
Gene Ontology Term
Nucleoside−Triphosphatase Regulator Activity
Gtpase Regulator Activity
Guanyl−Nucleotide Exchange Factor Activity
Gtpase Activator Activity
Growth Factor Binding
Gtpase Binding
Notch Binding
Transmembrane Receptor Protein Kinase Activity
Small Gtpase Binding
Cell Adhesion Molecule Binding
0.0 2.5 5.0 7.5
−log10 adjusted p−value
Gene Count
15 12 9 6 3
Stromal_2 CC ontology
Gene Ontology Term
Membrane Raft
Membrane Microdomain
Actomyosin
Actin Cytoskeleton
Receptor Complex
Cell Leading Edge
Caveola
Postsynaptic Density
Basement Membrane
Basal Part Of Cell
0 1 2
−log10 adjusted p−value
Gene Count
9 8 7 6 5 4
Pericyte_2 BP ontology
Gene Ontology Term
Chromosome Segregation
Nuclear Chromosome Segregation
Nuclear Division
Mitotic Nuclear Division
Sister Chromatid Segregation
Mitotic Sister Chromatid Segregation
Spindle Organization
Regulation Of Chromosome Segregation
Chromosome Separation
Microtubule Cytoskeleton Organization Involved In Mitosis
0 10 20 30
−log10 adjusted p−value
Gene Count
30 25 20 15

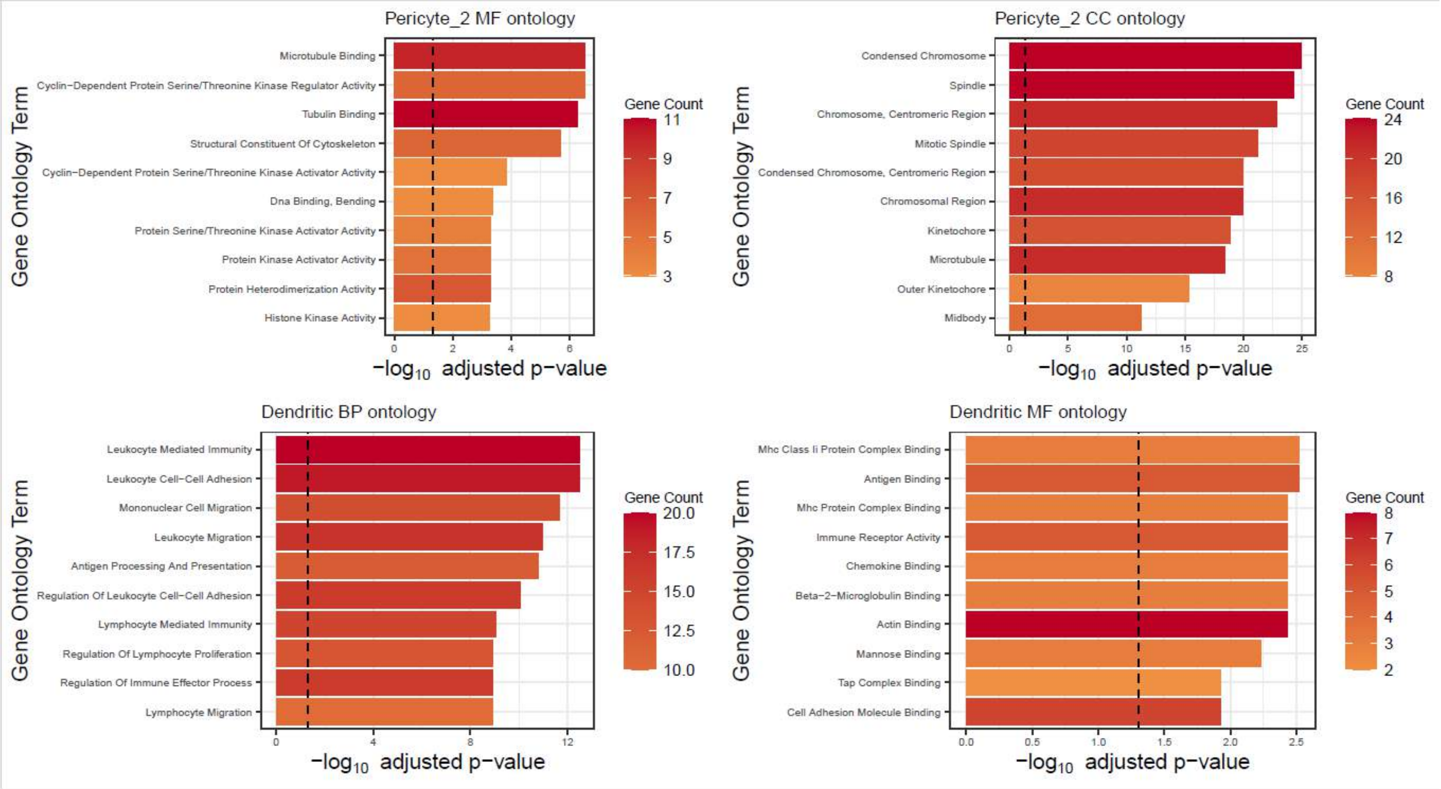
Pericyte_2 MF ontology
Microtubule Binding
Cyclin−Dependent Protein Serine/Threonine Kinase Regulator Activity
Tubulin Binding
Structural Constituent Of Cytoskeleton
Cyclin−Dependent Protein Serine/Threonine Kinase Activator Activity
Dna Binding, Bending
Protein Serine/Threonine Kinase Activator Activity
Protein Kinase Activator Activity
Protein Heterodimerization Activity
Histone Kinase Activity
Gene Ontology Term
−log10 adjusted p−value
Gene Count
Pericyte_2 CC ontology
Condensed Chromosome
Spindle
Chromosome, Centromeric Region
Mitotic Spindle
Condensed Chromosome, Centromeric Region
Chromosomal Region
Kinetochore
Microtubule
Outer Kinetochore
Midbody
Dendritic BP ontology
Leukocyte Mediated Immunity
Leukocyte Cell−Cell Adhesion
Mononuclear Cell Migration
Leukocyte Migration
Antigen Processing And Presentation
Regulation Of Leukocyte Cell−Cell Adhesion
Lymphocyte Mediated Immunity
Regulation Of Lymphocyte Proliferation
Regulation Of Immune Effector Process
Lymphocyte Migration
Dendritic MF ontology
Mhc Class Ii Protein Complex Binding
Antigen Binding
Mhc Protein Complex Binding
Immune Receptor Activity
Chemokine Binding
Beta−2−Microglobulin Binding
Actin Binding
Mannose Binding
Tap Complex Binding
Cell Adhesion Molecule Binding

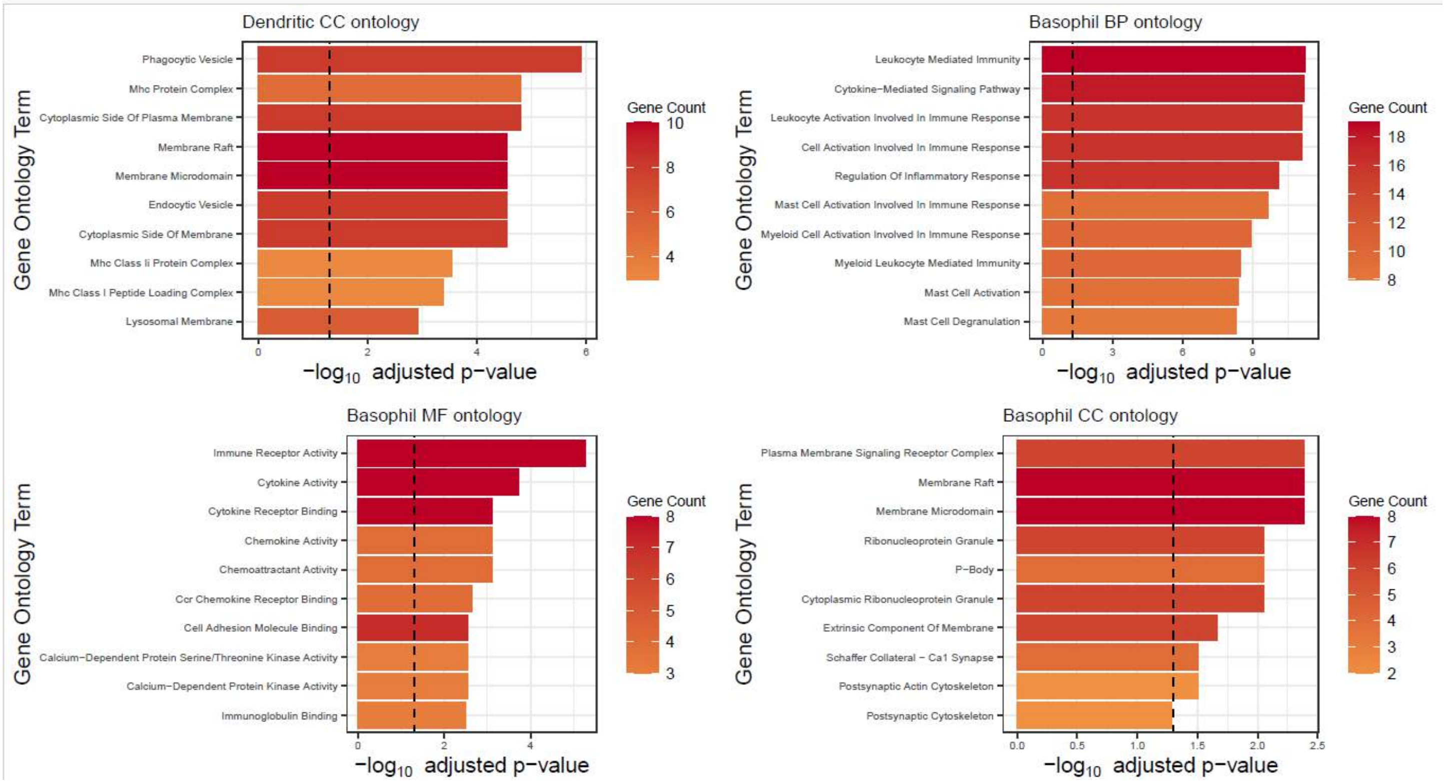
Dendritic CC ontology
Phagocytic Vesicle
Mhc Protein Complex
Cytoplasmic Side Of Plasma Membrane
Membrane Raft
Membrane Microdomain
Endocytic Vesicle
Cytoplasmic Side Of Membrane
Mhc Class Ii Protein Complex
Mhc Class I Peptide Loading Complex
Lysosomal Membrane
Gene Ontology Term
−log10 adjusted p−value
Gene Count
Basophil BP ontology
Leukocyte Mediated Immunity
Cytokine−Mediated Signaling Pathway
Leukocyte Activation Involved In Immune Response
Cell Activation Involved In Immune Response
Regulation Of Inflammatory Response
Mast Cell Activation Involved In Immune Response
Myeloid Cell Activation Involved In Immune Response
Myeloid Leukocyte Mediated Immunity
Mast Cell Activation
Mast Cell Degranulation
Basophil MF ontology
Immune Receptor Activity
Cytokine Activity
Cytokine Receptor Binding
Chemokine Activity
Chemoattractant Activity
Ccr Chemokine Receptor Binding
Cell Adhesion Molecule Binding
Calcium−Dependent Protein Serine/Threonine Kinase Activity
Calcium−Dependent Protein Kinase Activity
Immunoglobulin Binding
Basophil CC ontology
Plasma Membrane Signaling Receptor Complex
Membrane Raft
Membrane Microdomain
Ribonucleoprotein Granule
P−Body
Cytoplasmic Ribonucleoprotein Granule
Extrinsic Component Of Membrane
Schaffer Collateral − Ca1 Synapse
Postsynaptic Actin Cytoskeleton
Postsynaptic Cytoskeleton

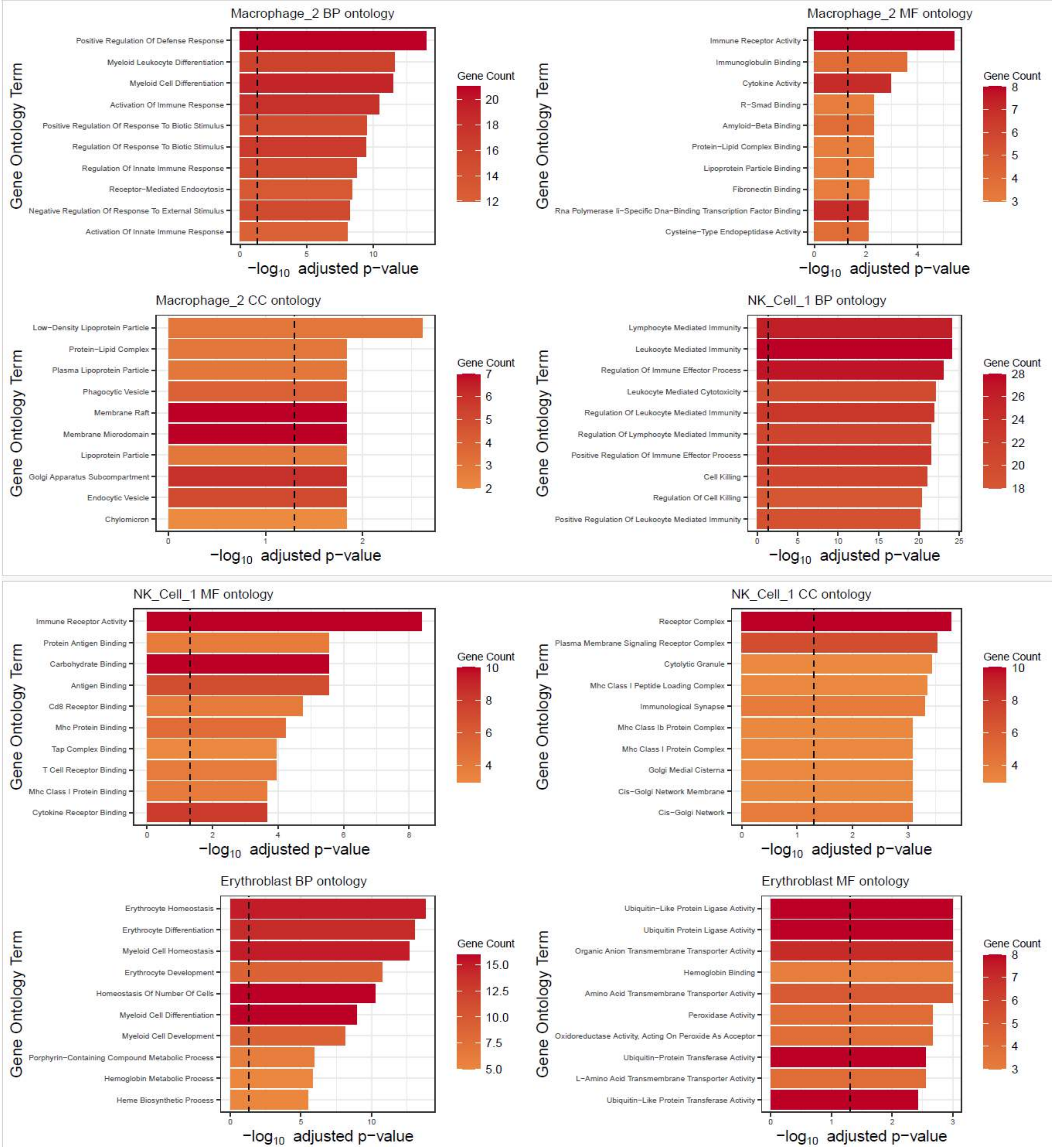
Macrophage_2 BP ontology
Positive Regulation Of Defense Response
Myeloid Leukocyte Differentiation
Myeloid Cell Differentiation
Activation Of Immune Response
Positive Regulation Of Response To Biotic Stimulus
Regulation Of Response To Biotic Stimulus
Regulation Of Innate Immune Response
Receptor-Mediated Endocytosis
Negative Regulation Of Response To External Stimulus
Activation Of Innate Immune Response
Gene Ontology Term
−log10 adjusted p−value
Gene Count
Macrophage_2 MF ontology
Immune Receptor Activity
Immunoglobulin Binding
Cytokine Activity
R-Smad Binding
Amyloid-Beta Binding
Protein-Lipid Complex Binding
Lipoprotein Particle Binding
Fibronectin Binding
Rna Polymerase Ii-Specific Dna-Binding Transcription Factor Binding
Cysteine-Type Endopeptidase Activity
Macrophage_2 CC ontology
Low-Density Lipoprotein Particle
Protein-Lipid Complex
Plasma Lipoprotein Particle
Phagocytic Vesicle
Membrane Raft
Membrane Microdomain
Lipoprotein Particle
Golgi Apparatus Subcompartment
Endocytic Vesicle
Chylomicron
NK_Cell_1 BP ontology
Lymphocyte Mediated Immunity
Leukocyte Mediated Immunity
Regulation Of Immune Effector Process
Leukocyte Mediated Cytotoxicity
Regulation Of Leukocyte Mediated Immunity
Regulation Of Lymphocyte Mediated Immunity
Positive Regulation Of Immune Effector Process
Cell Killing
Regulation Of Cell Killing
Positive Regulation Of Leukocyte Mediated Immunity
NK_Cell_1 MF ontology
Immune Receptor Activity
Protein Antigen Binding
Carbohydrate Binding
Antigen Binding
Cd8 Receptor Binding
Mhc Protein Binding
Tap Complex Binding
T Cell Receptor Binding
Mhc Class I Protein Binding
Cytokine Receptor Binding
NK_Cell_1 CC ontology
Receptor Complex
Plasma Membrane Signaling Receptor Complex
Cytolytic Granule
Mhc Class I Peptide Loading Complex
Immunological Synapse
Mhc Class Ib Protein Complex
Mhc Class I Protein Complex
Golgi Medial Cisterna
Cis-Golgi Network Membrane
Cis-Golgi Network
Erythroblast BP ontology
Erythrocyte Homeostasis
Erythrocyte Differentiation
Myeloid Cell Homeostasis
Erythrocyte Development
Homeostasis Of Number Of Cells
Myeloid Cell Differentiation
Myeloid Cell Development
Porphyrin-Containing Compound Metabolic Process
Hemoglobin Metabolic Process
Heme Biosynthetic Process
Erythroblast MF ontology
Ubiquitin-Like Protein Ligase Activity
Ubiquitin Protein Ligase Activity
Organic Anion Transmembrane Transporter Activity
Hemoglobin Binding
Amino Acid Transmembrane Transporter Activity
Peroxidase Activity
Oxidoreductase Activity, Acting On Peroxide As Acceptor
Ubiquitin-Protein Transferase Activity
L-Amino Acid Transmembrane Transporter Activity
Ubiquitin-Like Protein Transferase Activity

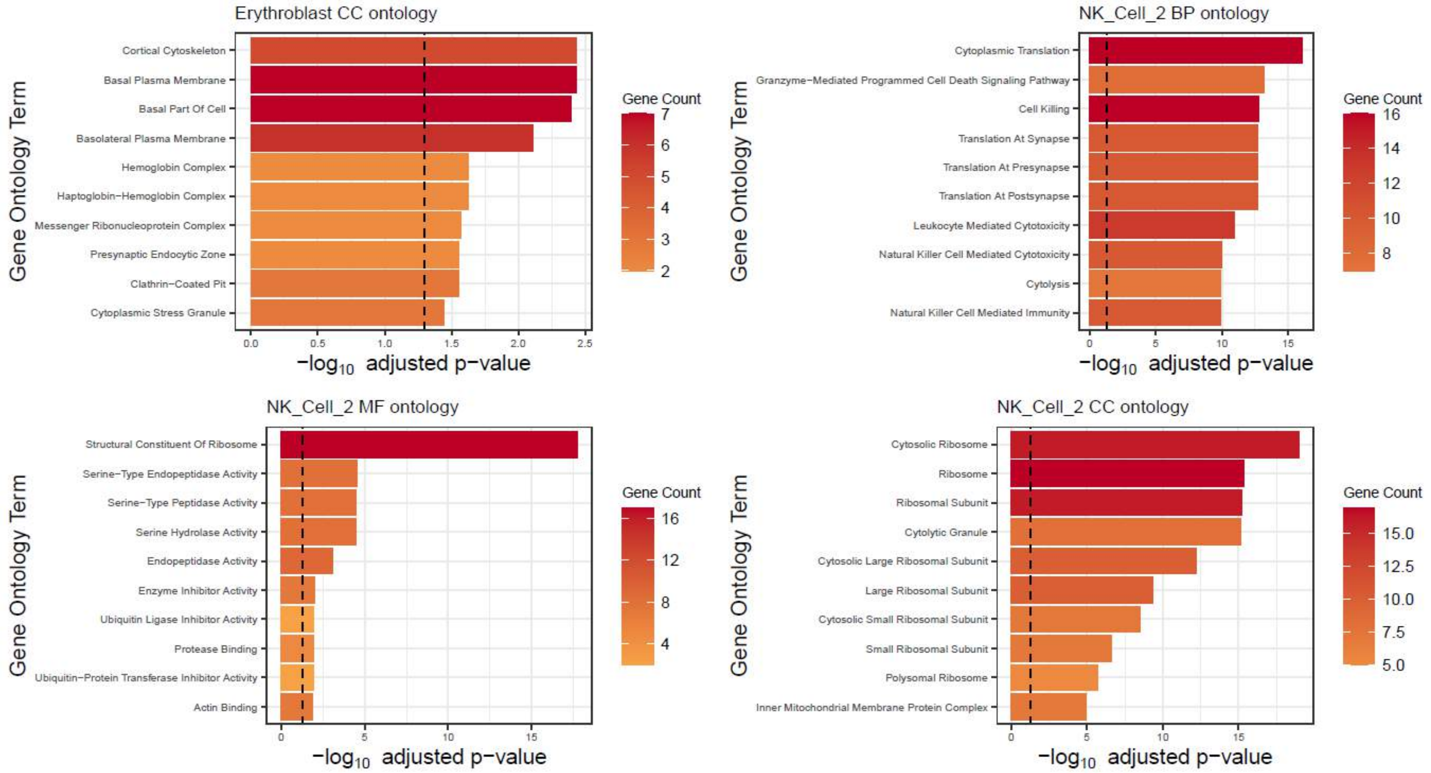

Supplementary Fig. 12. GO term analyses comparing cell types to one another. BP: Biological Processes; MF: Molecular Function; CC: Cellular Component. GO term analyses are based on the hypergeometric test, with two-sided p-values adjusting for multiple comparisons via the Benjamini-Hochberg method to control the False Discovery Rate.

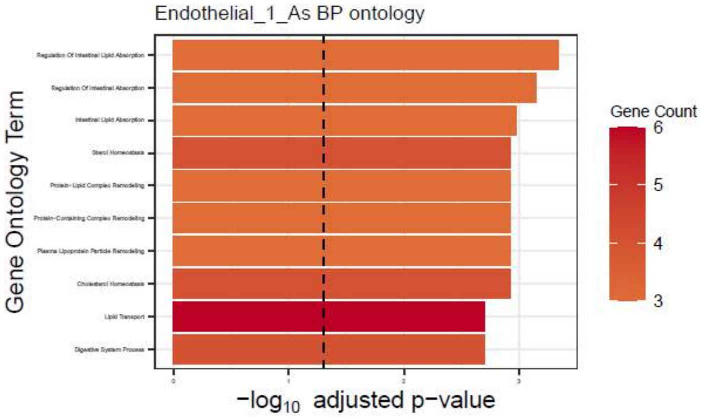
Endothelial_1_As BP ontology
Gene Ontology Term
−log10 adjusted p−value
Gene Count
6
5
4
3

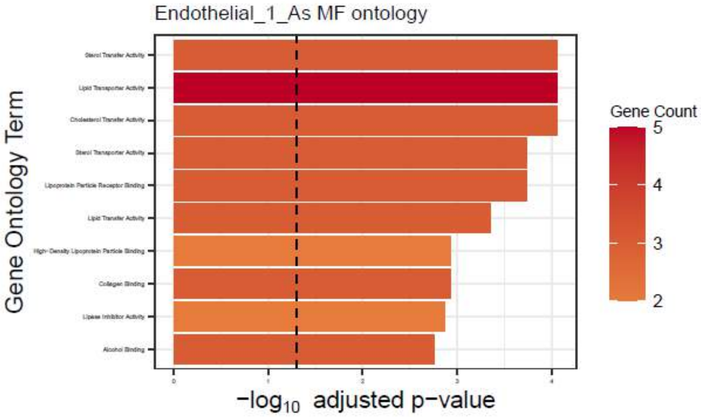
Endothelial_1_As MF ontology
Gene Ontology Term
−log10 adjusted p−value
Gene Count
5
4
3
2

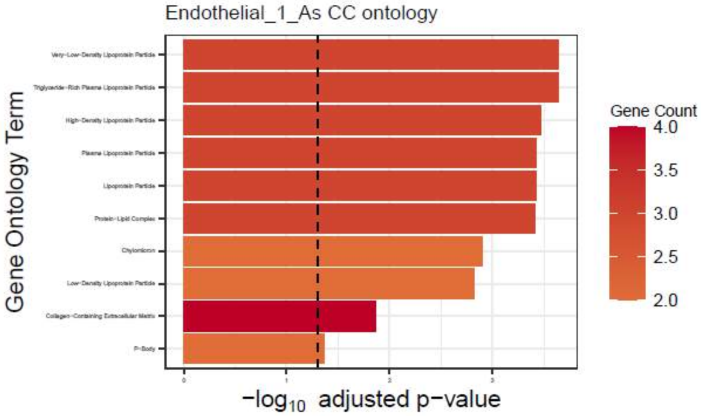
Endothelial_1_As CC ontology
Gene Ontology Term
−log10 adjusted p−value
Gene Count
4.0
3.5
3.0
2.5
2.0

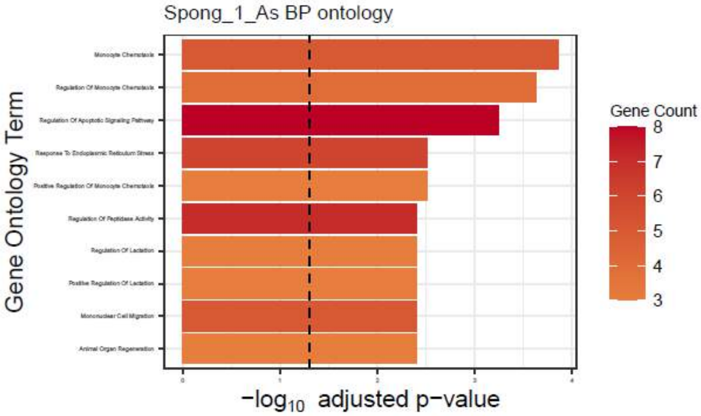
Spong_1_As BP ontology
Gene Ontology Term
−log10 adjusted p−value
Gene Count
8
7
6
5
4
3

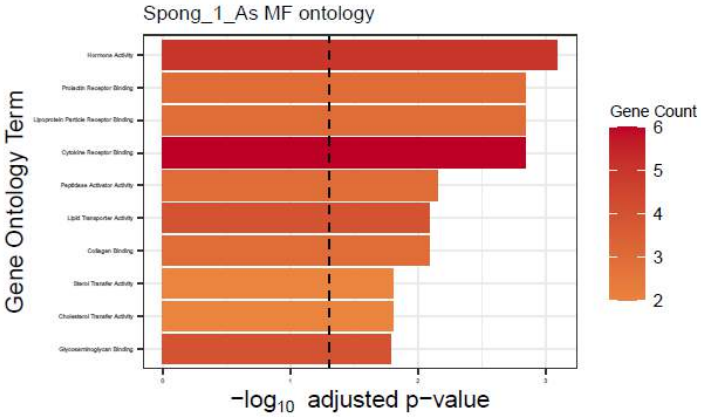
Spong_1_As MF ontology
Gene Ontology Term
−log10 adjusted p−value
Gene Count
6
5
4
3
2

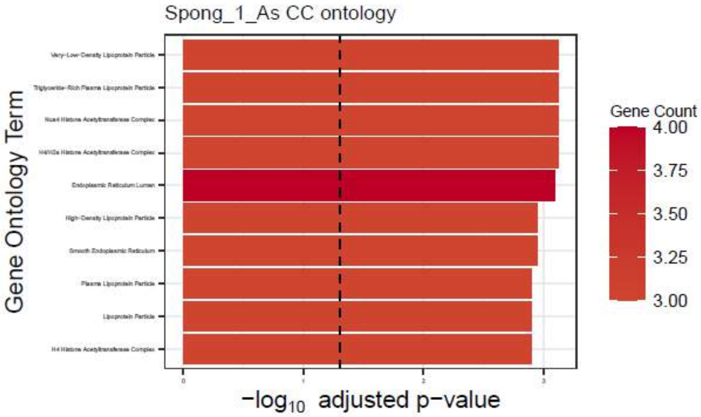
Spong_1_As CC ontology
Gene Ontology Term
−log10 adjusted p−value
Gene Count
4.00
3.75
3.50
3.25
3.00

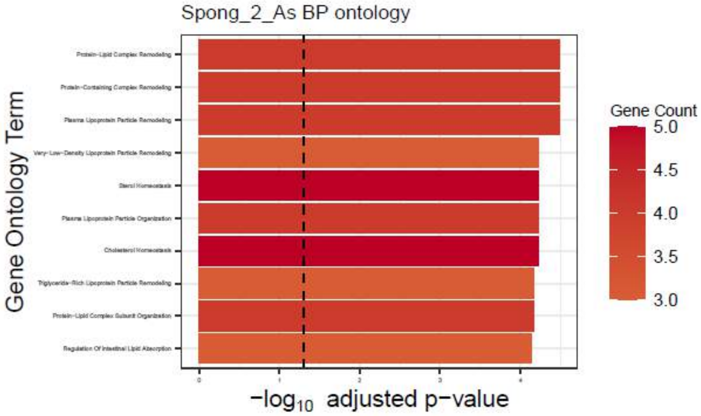
Spong_2_As BP ontology
Gene Ontology Term
−log10 adjusted p−value
Gene Count
5.0
4.5
4.0
3.5
3.0

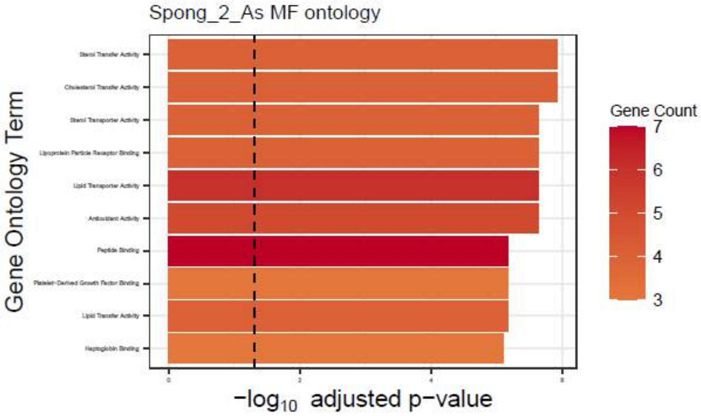
Spong_2_As MF ontology
Gene Ontology Term
−log10 adjusted p−value
Gene Count
7
6
5
4
3

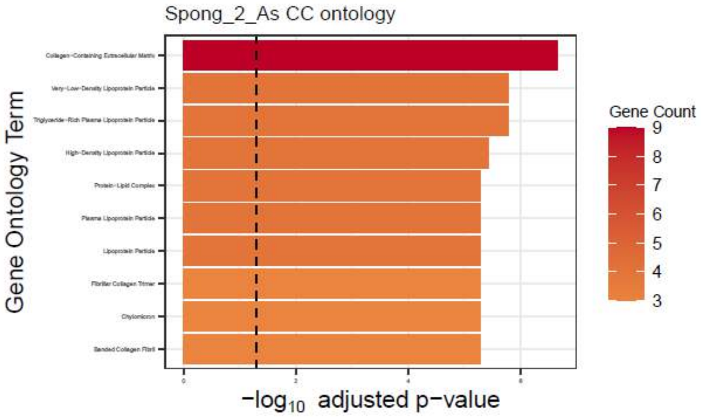
Spong_2_As CC ontology
Gene Ontology Term
−log10 adjusted p−value
Gene Count
9
8
7
6
5
4
3

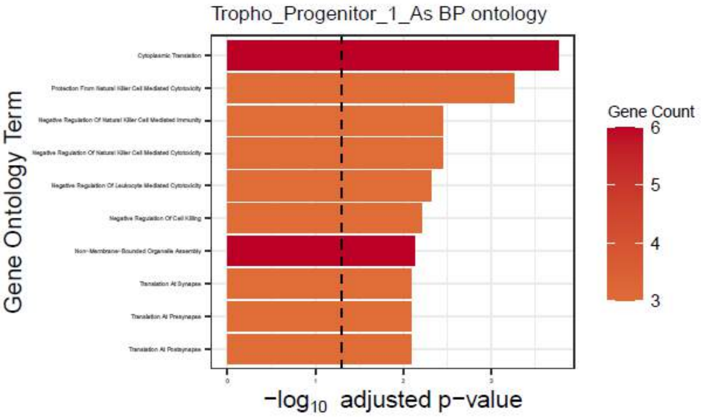
Tropho_Progenitor_1_As BP ontology
Gene Ontology Term
−log10 adjusted p−value
Gene Count
6
5
4
3

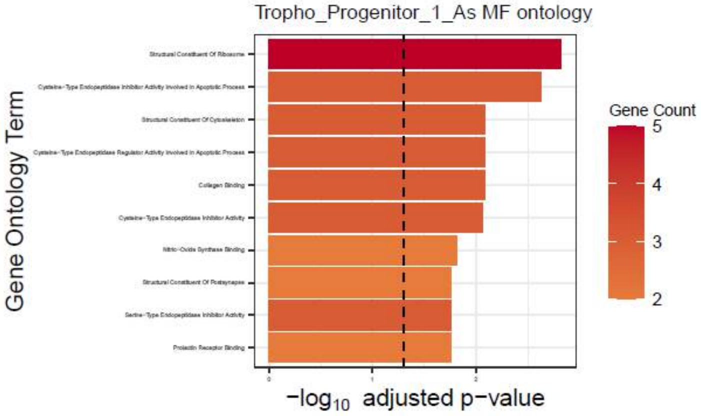
Tropho_Progenitor_1_As MF ontology
Gene Ontology Term
−log10 adjusted p−value
Gene Count
5
4
3
2

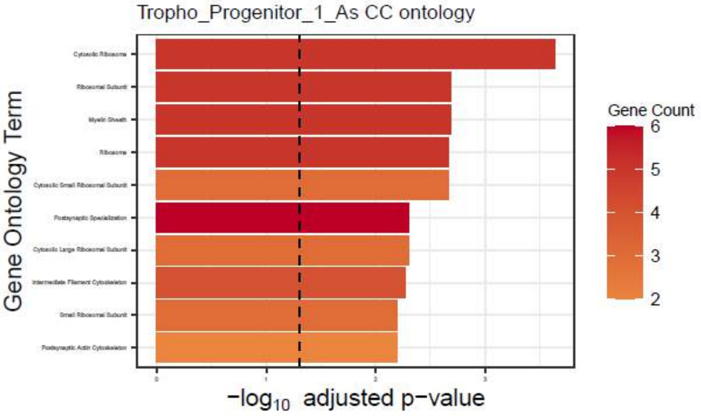
Tropho_Progenitor_1_As CC ontology
Gene Ontology Term
−log10 adjusted p−value
Gene Count
6
5
4
3
2

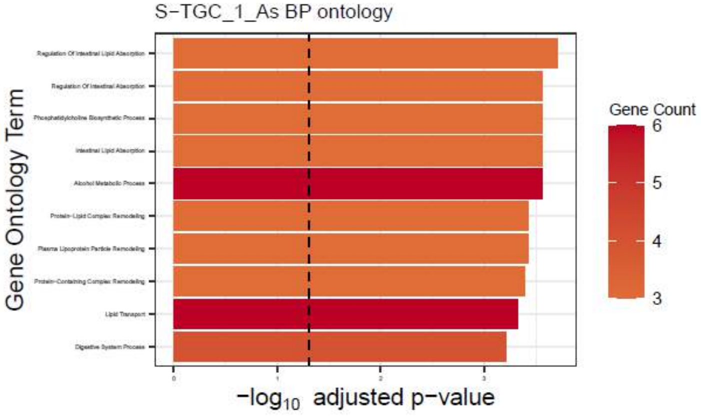
S−TGC_1_As BP ontology
Gene Ontology Term
−log10 adjusted p−value
Gene Count
6
5
4
3

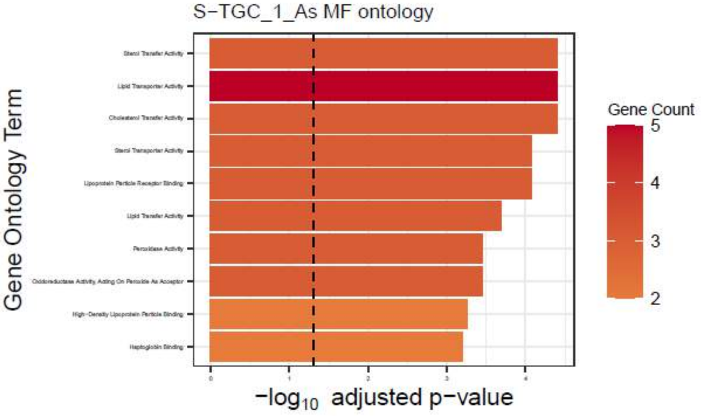
S−TGC_1_As MF ontology
Gene Ontology Term
−log10 adjusted p−value
Gene Count
5
4
3
2

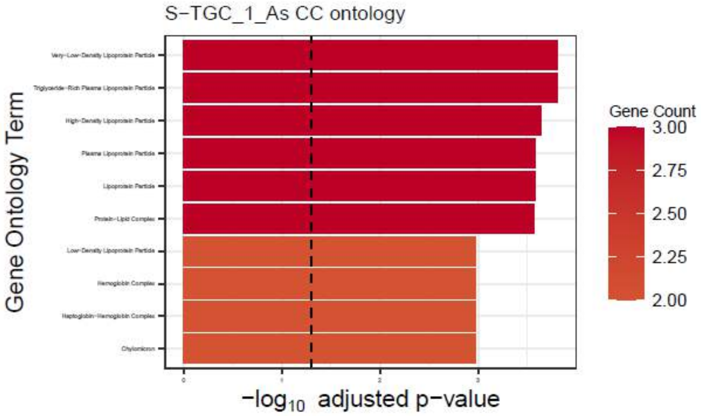
S−TGC_1_As CC ontology
Gene Ontology Term
−log10 adjusted p−value
Gene Count
3.00
2.75
2.50
2.25
2.00

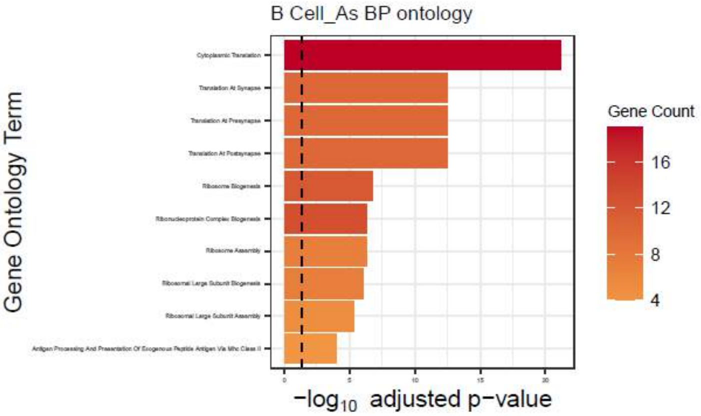
B Cell_As BP ontology
Gene Ontology Term
−log10 adjusted p−value
Gene Count
16
12
8
4

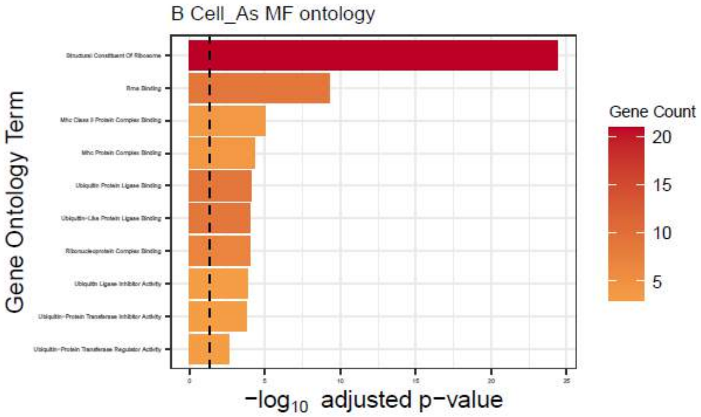
B Cell_As MF ontology
Gene Ontology Term
Gene Count
20
15
10
5
$-\log_{10}$ adjusted p−value

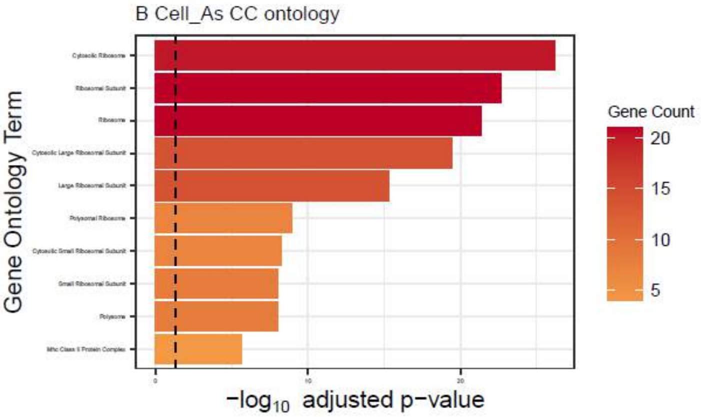
B Cell_As CC ontology
Gene Ontology Term
Gene Count
20
15
10
5
$-\log_{10}$ adjusted p−value

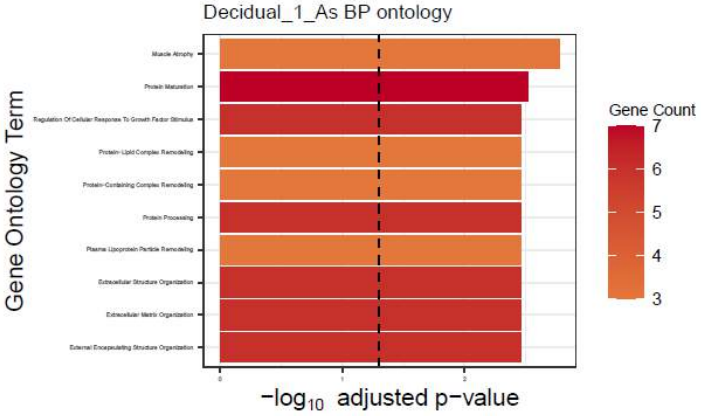
Decidual_1_As BP ontology
Gene Ontology Term
Gene Count
7
6
5
4
3
$-\log_{10}$ adjusted p−value

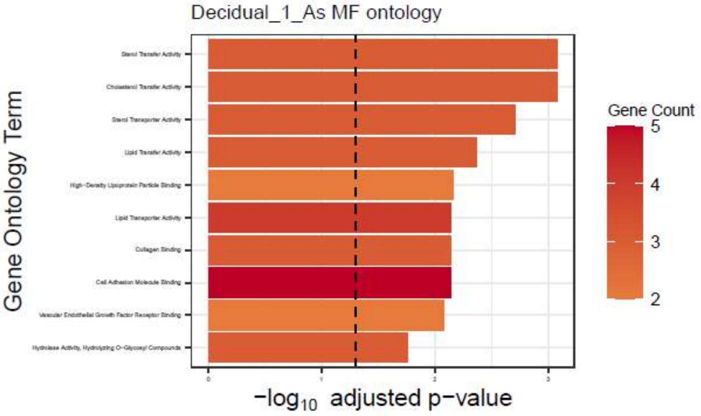
Decidual_1_As MF ontology
Gene Ontology Term
Gene Count
5
4
3
2
$-\log_{10}$ adjusted p−value

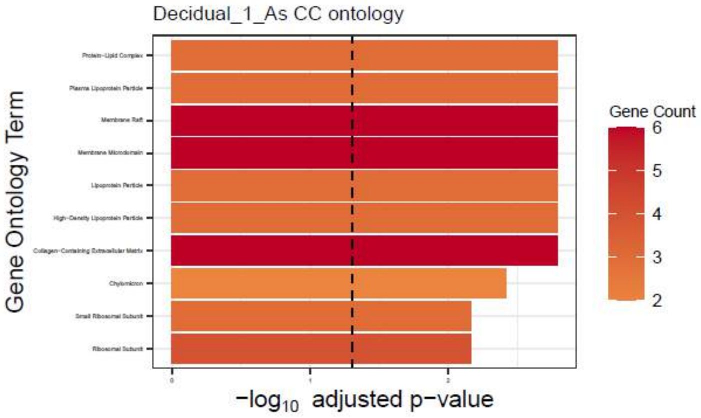
Decidual_1_As CC ontology
Gene Ontology Term
Gene Count
6
5
4
3
2
$-\log_{10}$ adjusted p−value

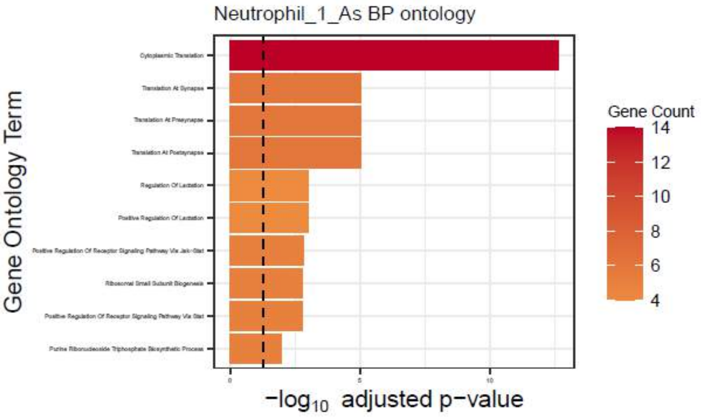
Neutrophil_1_As BP ontology
Gene Ontology Term
Gene Count
14
12
10
8
6
4
$-\log_{10}$ adjusted p−value

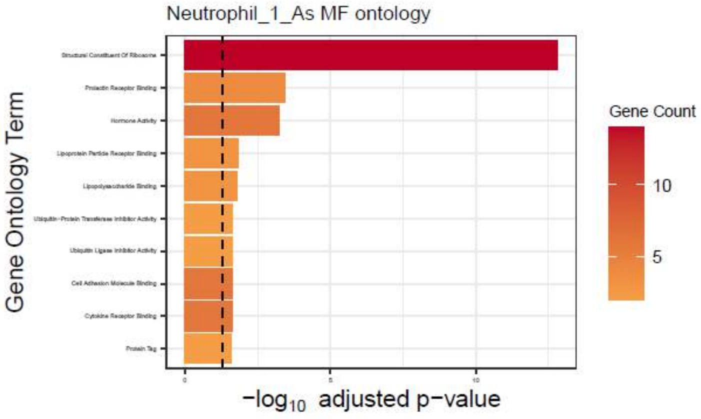
Neutrophil_1_As MF ontology
Gene Ontology Term
Gene Count
10
5
$-\log_{10}$ adjusted p−value

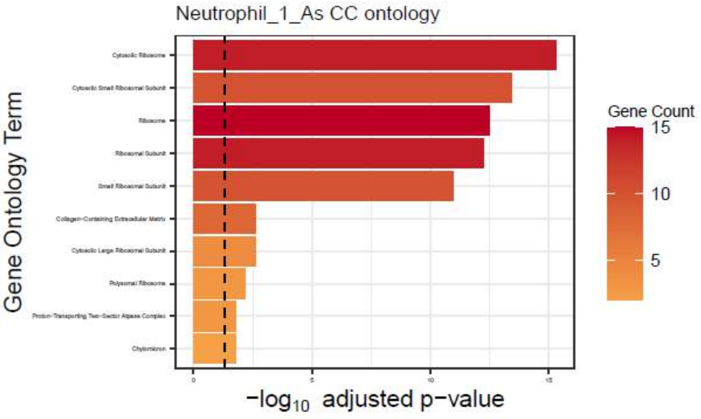
Neutrophil_1_As CC ontology
Gene Ontology Term
Gene Count
15
10
5
$-\log_{10}$ adjusted p−value

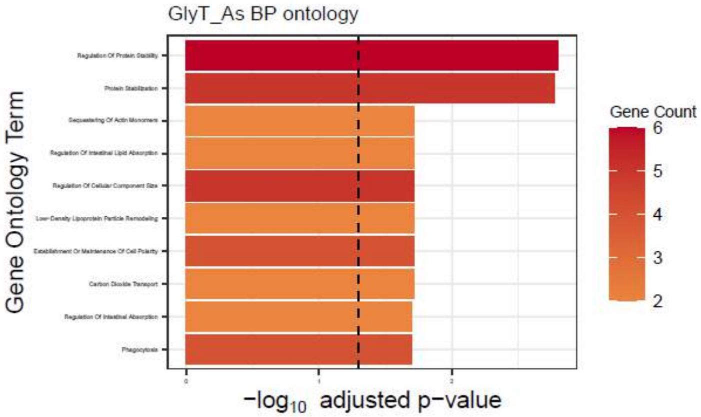
GlyT_As BP ontology
Gene Ontology Term
−log10 adjusted p−value
Gene Count
6
5
4
3
2

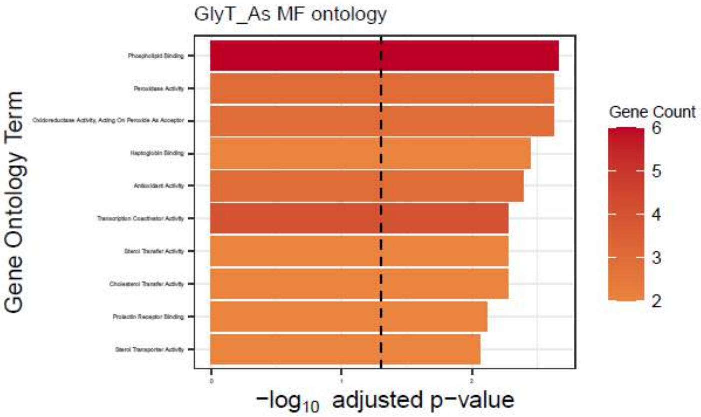
GlyT_As MF ontology
Gene Ontology Term
−log10 adjusted p−value
Gene Count
6
5
4
3
2

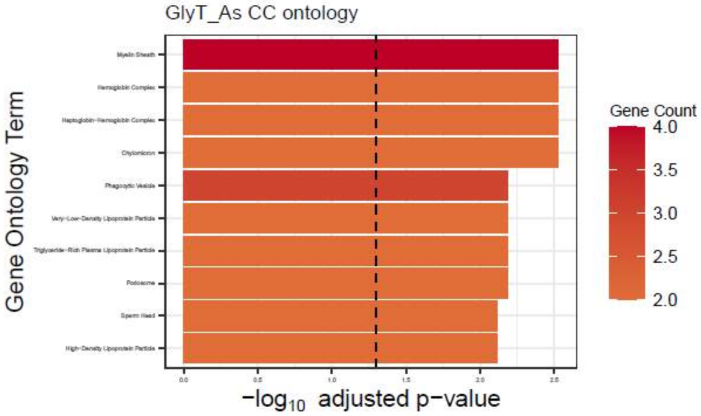
GlyT_As CC ontology
Gene Ontology Term
−log10 adjusted p−value
Gene Count
4.0
3.5
3.0
2.5
2.0

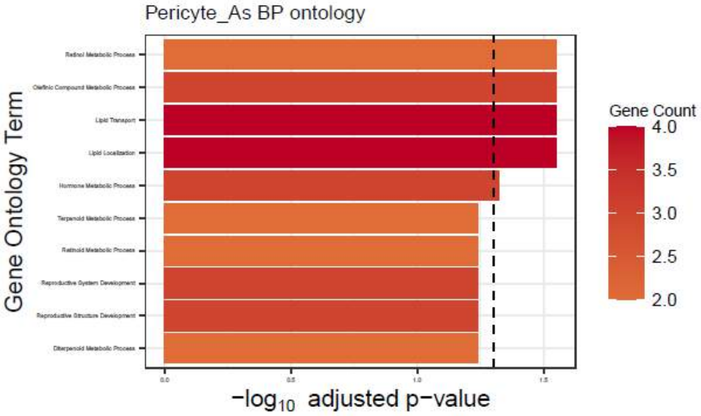
Pericyte_As BP ontology
Gene Ontology Term
−log10 adjusted p−value
Gene Count
4.0
3.5
3.0
2.5
2.0

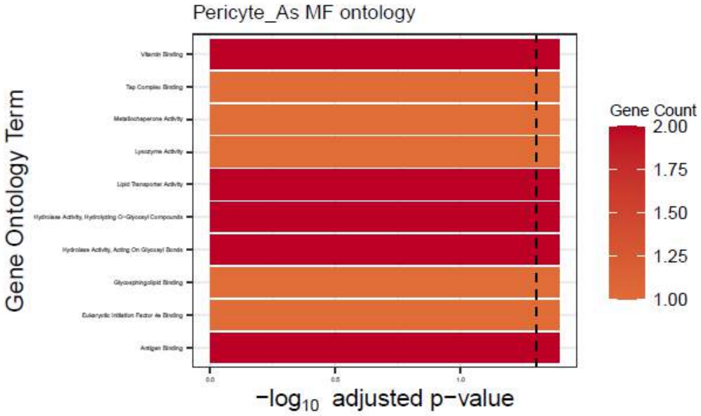
Pericyte_As MF ontology
Gene Ontology Term
−log10 adjusted p−value
Gene Count
2.00
1.75
1.50
1.25
1.00

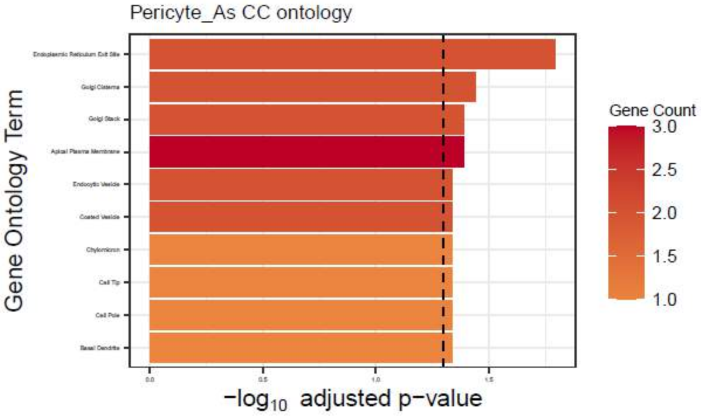
Pericyte_As CC ontology
Gene Ontology Term
−log10 adjusted p−value
Gene Count
3.0
2.5
2.0
1.5
1.0

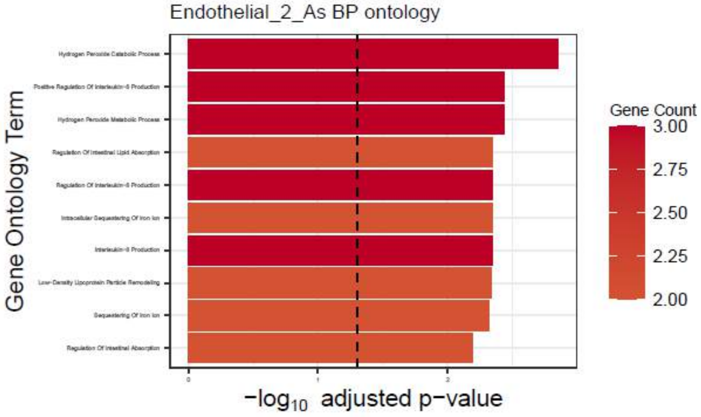
Endothelial_2_As BP ontology
Gene Ontology Term
−log10 adjusted p−value
Gene Count
3.00
2.75
2.50
2.25
2.00

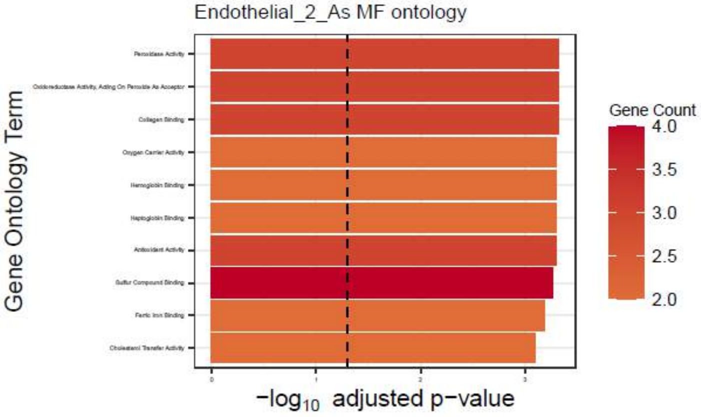
Endothelial_2_As MF ontology
Gene Ontology Term
−log10 adjusted p−value
Gene Count
4.0
3.5
3.0
2.5
2.0

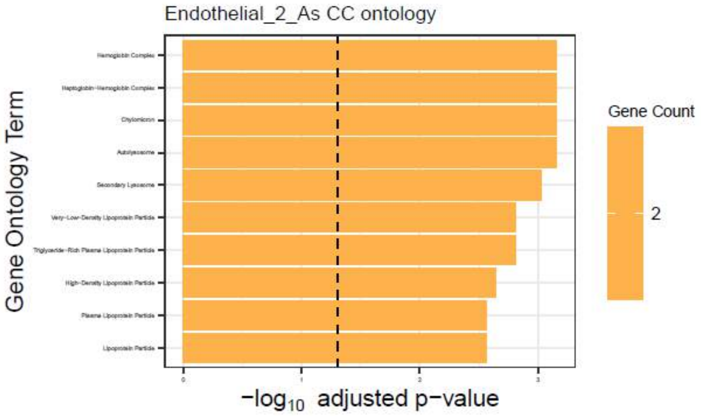
Endothelial_2_As CC ontology
Gene Ontology Term
Hemoglobin Complex
Haptoglobin−Hemoglobin Complex
Chylomicron
Autolysosome
Secondary Lysosome
Very−Low−Density Lipoprotein Particle
Triglyceride−Rich Plasma Lipoprotein Particle
High−Density Lipoprotein Particle
Plasma Lipoprotein Particle
Lipoprotein Particle
0
1
2
3
$-\log_{10}$ adjusted p−value
Gene Count
2

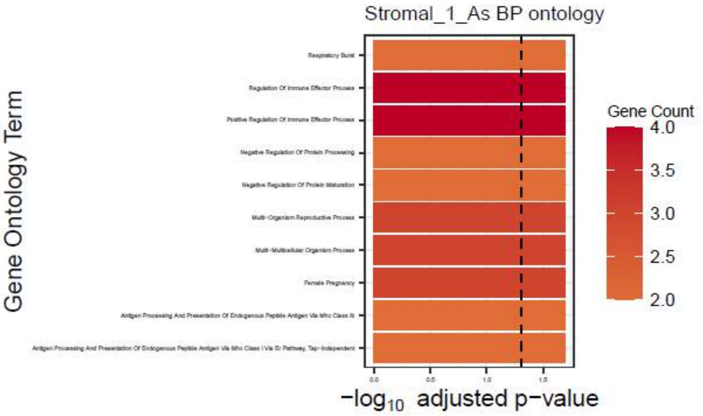
Stromal_1_As BP ontology
Gene Ontology Term
Respiratory Burst
Regulation Of Immune Effector Process
Positive Regulation Of Immune Effector Process
Negative Regulation Of Protein Processing
Negative Regulation Of Protein Maturation
Multi−Organism Reproductive Process
Multi−Multicellular Organism Process
Female Pregnancy
Antigen Processing And Presentation Of Endogenous Peptide Antigen Via Mhc Class Ib
Antigen Processing And Presentation Of Endogenous Peptide Antigen Via Mhc Class I Via Er Pathway, Tap−Independent
0.0
0.5
1.0
1.5
$-\log_{10}$ adjusted p−value
Gene Count
4.0
3.5
3.0
2.5
2.0

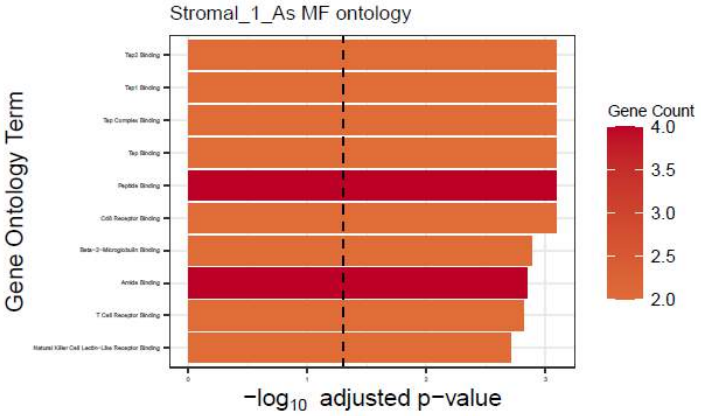
Stromal_1_As MF ontology
Gene Ontology Term
Tap2 Binding
Tap1 Binding
Tap Complex Binding
Tap Binding
Peptide Binding
Cd8 Receptor Binding
Beta−2−Microglobulin Binding
Amide Binding
T Cell Receptor Binding
Natural Killer Cell Lectin−Like Receptor Binding
0
1
2
3
$-\log_{10}$ adjusted p−value
Gene Count
4.0
3.5
3.0
2.5
2.0

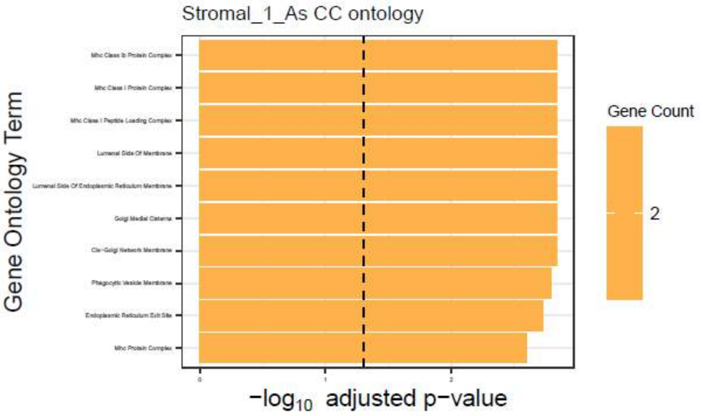
Stromal_1_As CC ontology
Gene Ontology Term
Mhc Class Ib Protein Complex
Mhc Class I Protein Complex
Mhc Class I Peptide Loading Complex
Lumenal Side Of Membrane
Lumenal Side Of Endoplasmic Reticulum Membrane
Golgi Medial Cisterna
Cis−Golgi Network Membrane
Phagocytic Vesicle Membrane
Endoplasmic Reticulum Exit Site
Mhc Protein Complex
0
1
2
$-\log_{10}$ adjusted p−value
Gene Count
2

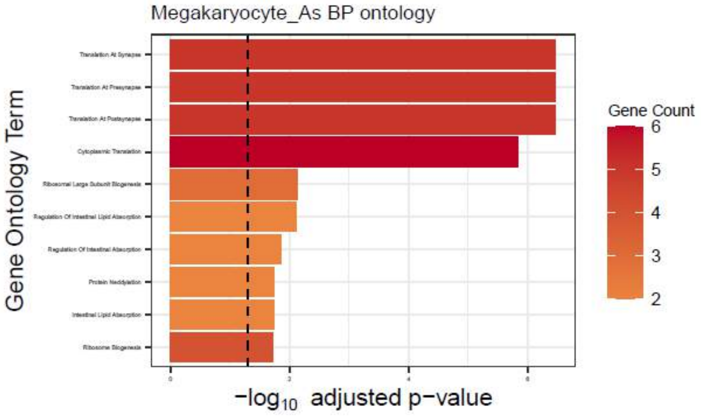
Megakaryocyte_As BP ontology
Gene Ontology Term
Translation At Synapse
Translation At Presynapse
Translation At Postsynapse
Cytoplasmic Translation
Ribosomal Large Subunit Biogenesis
Regulation Of Intestinal Lipid Absorption
Regulation Of Intestinal Absorption
Protein Neddylation
Intestinal Lipid Absorption
Ribosome Biogenesis
0
2
4
6
$-\log_{10}$ adjusted p−value
Gene Count
6
5
4
3
2

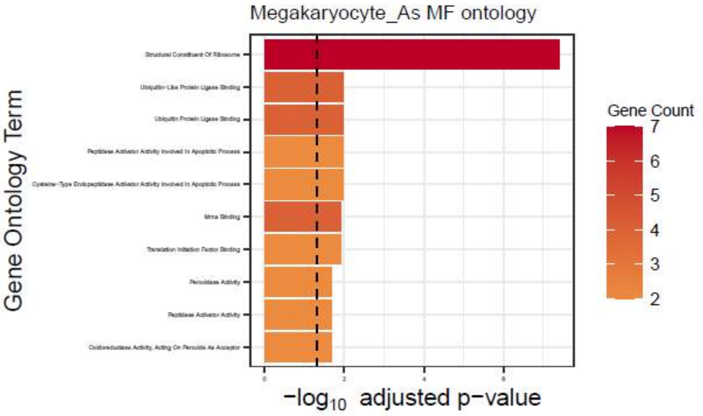
Megakaryocyte_As MF ontology
Gene Ontology Term
Structural Constituent Of Ribosome
Ubiquitin−Like Protein Ligase Binding
Ubiquitin Protein Ligase Binding
Peptidase Activator Activity Involved In Apoptotic Process
Cysteine−Type Endopeptidase Activator Activity Involved In Apoptotic Process
Mrna Binding
Translation Initiation Factor Binding
Peroxidase Activity
Peptidase Activator Activity
Oxidoreductase Activity, Acting On Peroxide As Acceptor
0
2
4
6
$-\log_{10}$ adjusted p−value
Gene Count
7
6
5
4
3
2

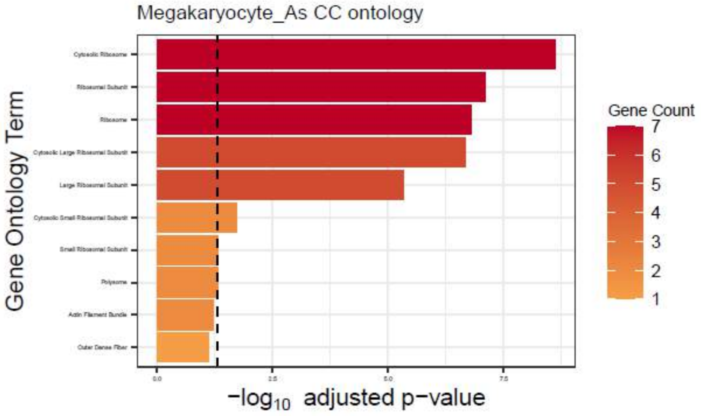
Megakaryocyte_As CC ontology
Gene Ontology Term
Cytosolic Ribosome
Ribosomal Subunit
Ribosome
Cytosolic Large Ribosomal Subunit
Large Ribosomal Subunit
Cytosolic Small Ribosomal Subunit
Small Ribosomal Subunit
Polysome
Actin Filament Bundle
Outer Dense Fiber
0.0
2.5
5.0
7.5
$-\log_{10}$ adjusted p−value
Gene Count
7
6
5
4
3
2
1

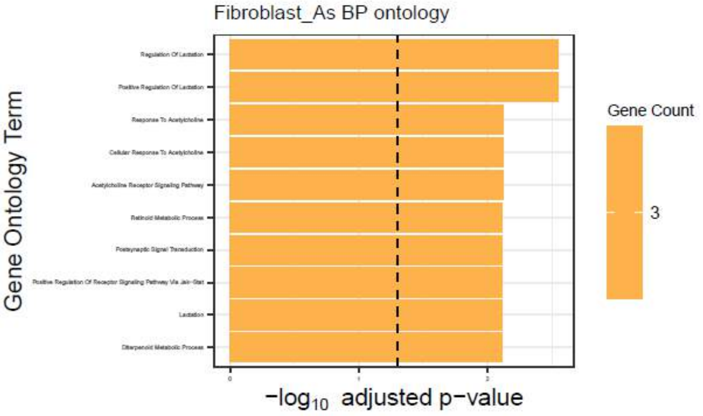
Fibroblast_As BP ontology
Gene Ontology Term
Regulation Of Lactation
Positive Regulation Of Lactation
Response To Acetylcholine
Cellular Response To Acetylcholine
Acetylcholine Receptor Signaling Pathway
Retinoid Metabolic Process
Postsynaptic Signal Transduction
Positive Regulation Of Receptor Signaling Pathway Via Jak−Stat
Lactation
Diterpenoid Metabolic Process
0
1
2
$-\log_{10}$ adjusted p−value
Gene Count
3

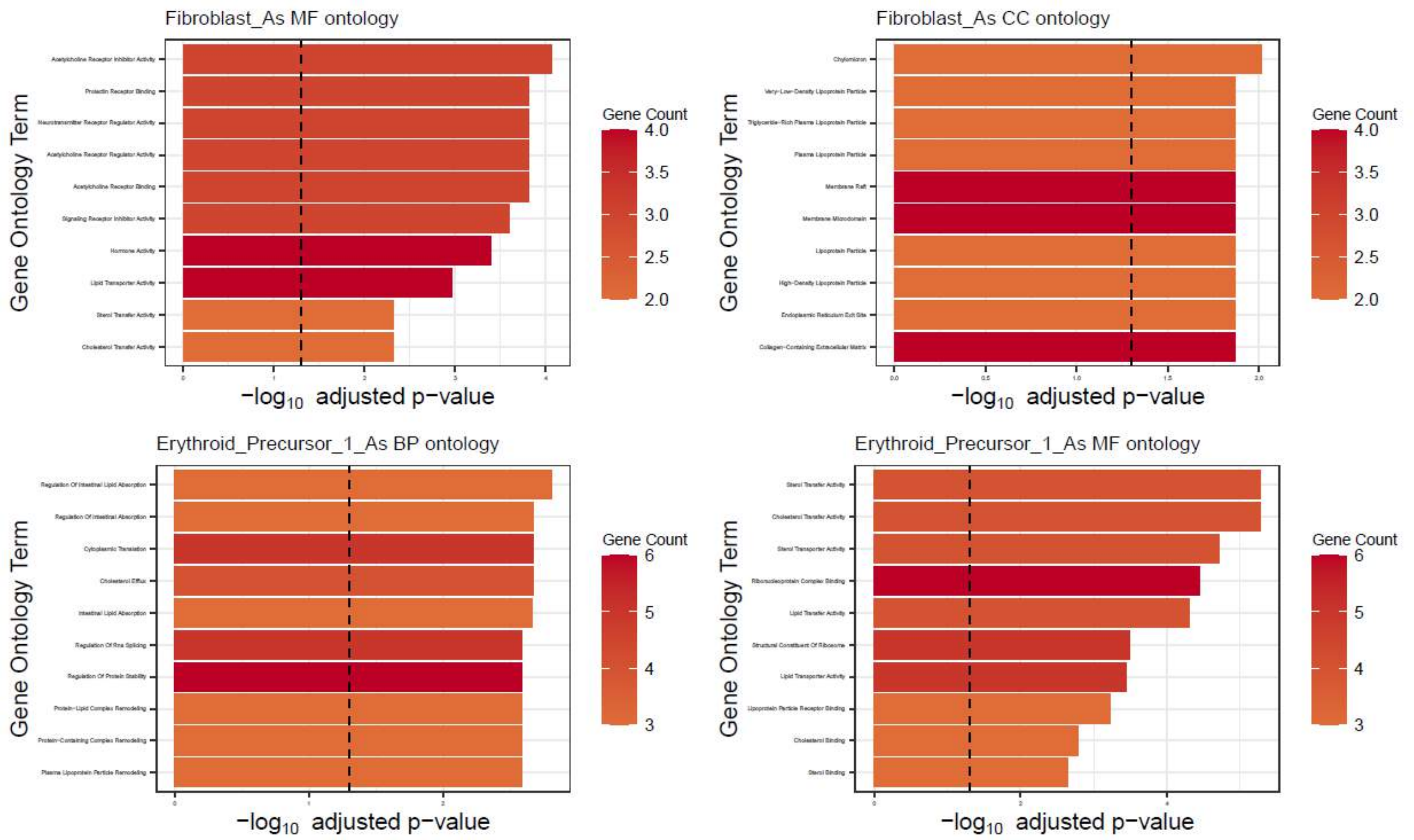
Fibroblast_As MF ontology
Fibroblast_As CC ontology
Erythroid_Precursor_1_As BP ontology
Erythroid_Precursor_1_As MF ontology
Gene Ontology Term
Gene Count
$-\log_{10}$ adjusted p−value

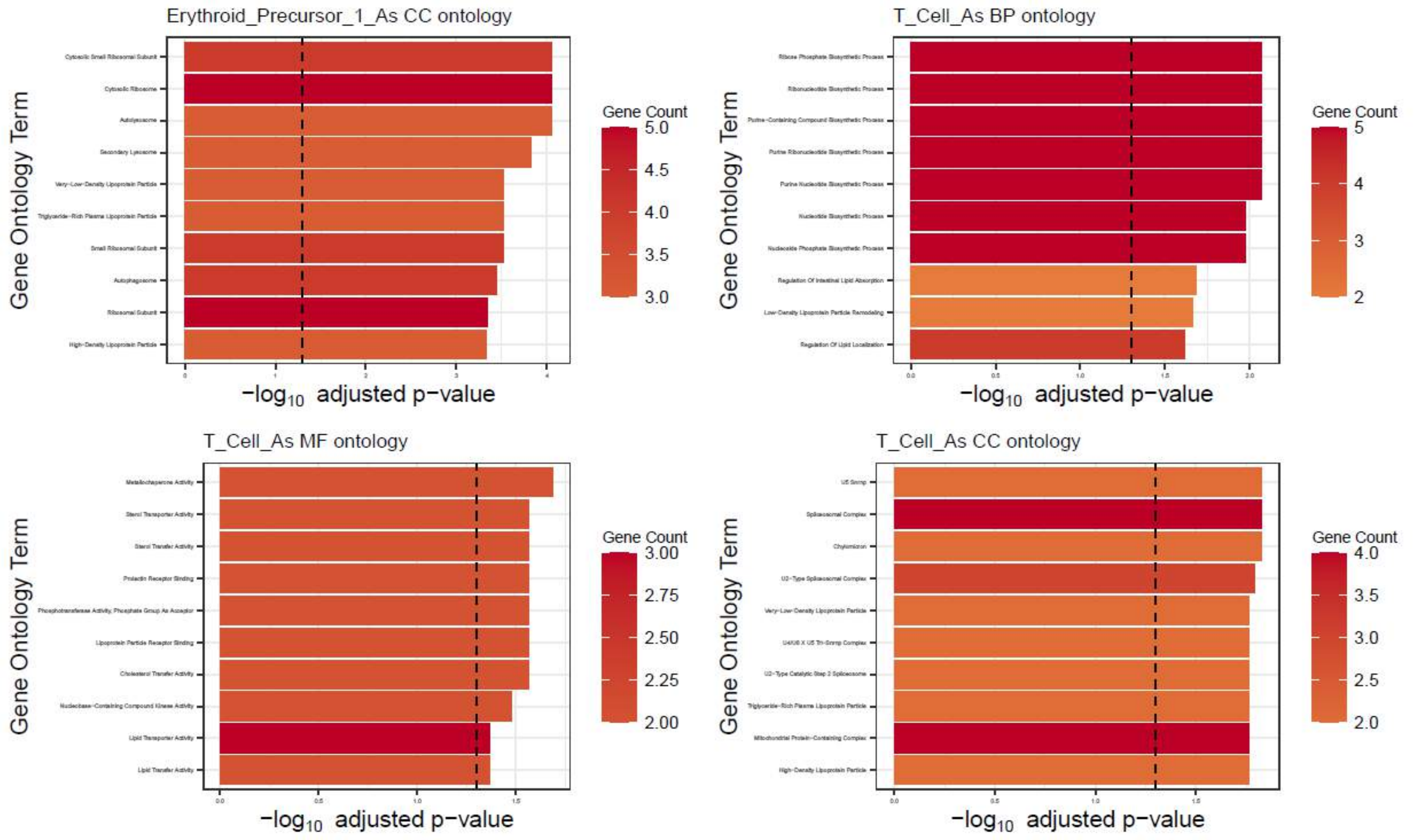
Erythroid_Precursor_1_As CC ontology
T_Cell_As BP ontology
T_Cell_As MF ontology
T_Cell_As CC ontology
Gene Ontology Term
Gene Count
$-\log_{10}$ adjusted p−value

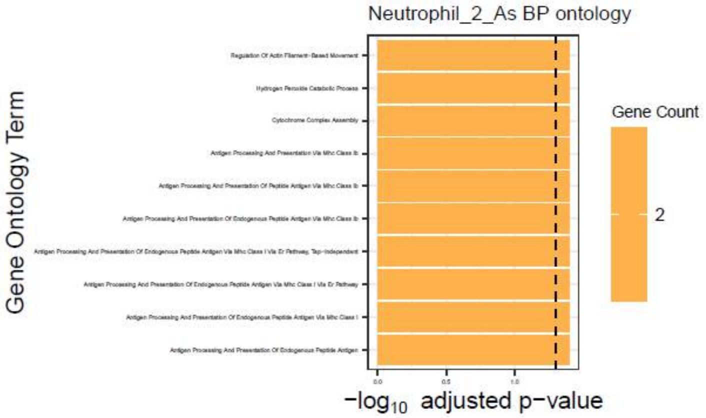
Neutrophil_2_As BP ontology
Gene Ontology Term
Regulation Of Actin Filament-Based Movement
Hydrogen Peroxide Catabolic Process
Cytochrome Complex Assembly
Antigen Processing And Presentation Via Mhc Class Ib
Antigen Processing And Presentation Of Peptide Antigen Via Mhc Class Ib
Antigen Processing And Presentation Of Endogenous Peptide Antigen Via Mhc Class Ib
Antigen Processing And Presentation Of Endogenous Peptide Antigen Via Mhc Class I Via Er Pathway, Tap-Independent
Antigen Processing And Presentation Of Endogenous Peptide Antigen Via Mhc Class I Via Er Pathway
Antigen Processing And Presentation Of Endogenous Peptide Antigen Via Mhc Class I
Antigen Processing And Presentation Of Endogenous Peptide Antigen
$-\log_{10}$ adjusted p-value
Gene Count
2

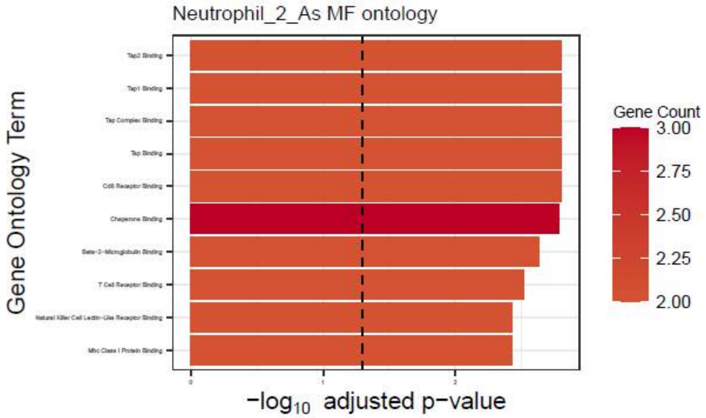
Neutrophil_2_As MF ontology
Gene Ontology Term
Tap2 Binding
Tap1 Binding
Tap Complex Binding
Tap Binding
Cd8 Receptor Binding
Chaperone Binding
Beta-2-Microglobulin Binding
T Cell Receptor Binding
Natural Killer Cell Lectin-Like Receptor Binding
Mhc Class I Protein Binding
$-\log_{10}$ adjusted p-value
Gene Count
3.00
2.75
2.50
2.25
2.00

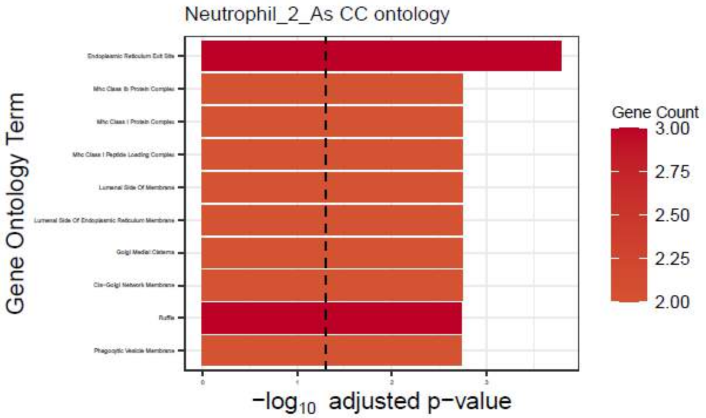
Neutrophil_2_As CC ontology
Gene Ontology Term
Endoplasmic Reticulum Exit Site
Mhc Class Ib Protein Complex
Mhc Class I Protein Complex
Mhc Class I Peptide Loading Complex
Lumenal Side Of Membrane
Lumenal Side Of Endoplasmic Reticulum Membrane
Golgi Medial Cisterna
Cis-Golgi Network Membrane
Ruffle
Phagocytic Vesicle Membrane
$-\log_{10}$ adjusted p-value
Gene Count
3.00
2.75
2.50
2.25
2.00

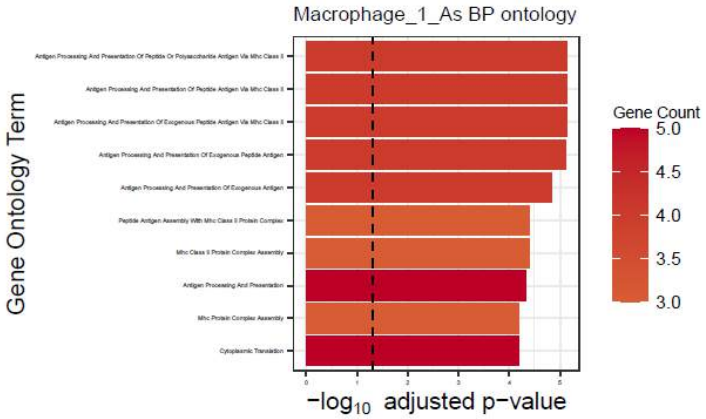
Macrophage_1_As BP ontology
Gene Ontology Term
Antigen Processing And Presentation Of Peptide Or Polysaccharide Antigen Via Mhc Class II
Antigen Processing And Presentation Of Peptide Antigen Via Mhc Class II
Antigen Processing And Presentation Of Exogenous Peptide Antigen Via Mhc Class II
Antigen Processing And Presentation Of Exogenous Peptide Antigen
Antigen Processing And Presentation Of Exogenous Antigen
Peptide Antigen Assembly With Mhc Class II Protein Complex
Mhc Class II Protein Complex Assembly
Antigen Processing And Presentation
Mhc Protein Complex Assembly
Cytoplasmic Translation
$-\log_{10}$ adjusted p-value
Gene Count
5.0
4.5
4.0
3.5
3.0

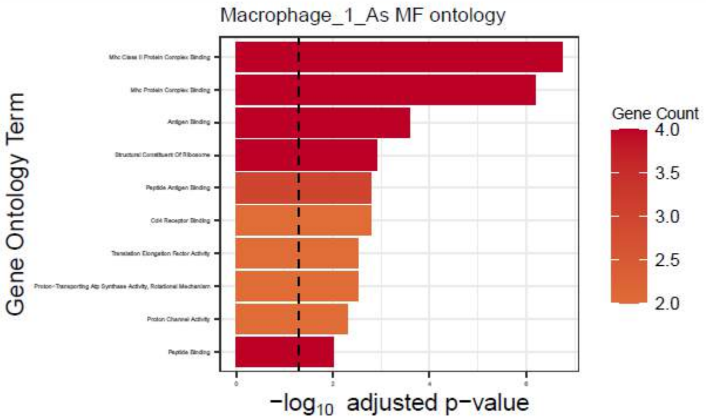
Macrophage_1_As MF ontology
Gene Ontology Term
Mhc Class II Protein Complex Binding
Mhc Protein Complex Binding
Antigen Binding
Structural Constituent Of Ribosome
Peptide Antigen Binding
Cd4 Receptor Binding
Translation Elongation Factor Activity
Proton-Transporting Atp Synthase Activity, Rotational Mechanism
Proton Channel Activity
Peptide Binding
$-\log_{10}$ adjusted p-value
Gene Count
4.0
3.5
3.0
2.5
2.0

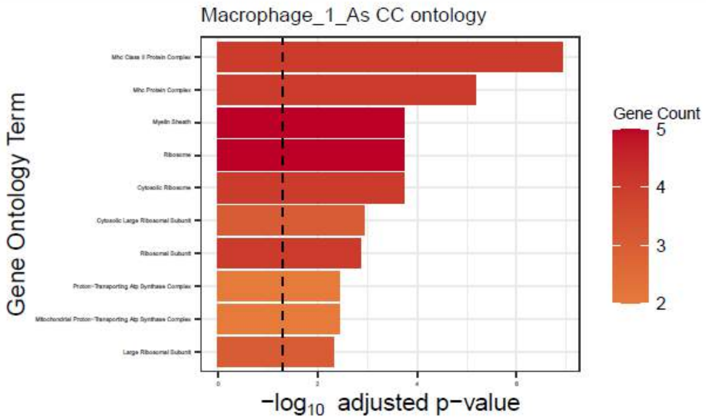
Macrophage_1_As CC ontology
Gene Ontology Term
Mhc Class II Protein Complex
Mhc Protein Complex
Myelin Sheath
Ribosome
Cytosolic Ribosome
Cytosolic Large Ribosomal Subunit
Ribosomal Subunit
Proton-Transporting Atp Synthase Complex
Mitochondrial Proton-Transporting Atp Synthase Complex
Large Ribosomal Subunit
$-\log_{10}$ adjusted p-value
Gene Count
5
4
3
2

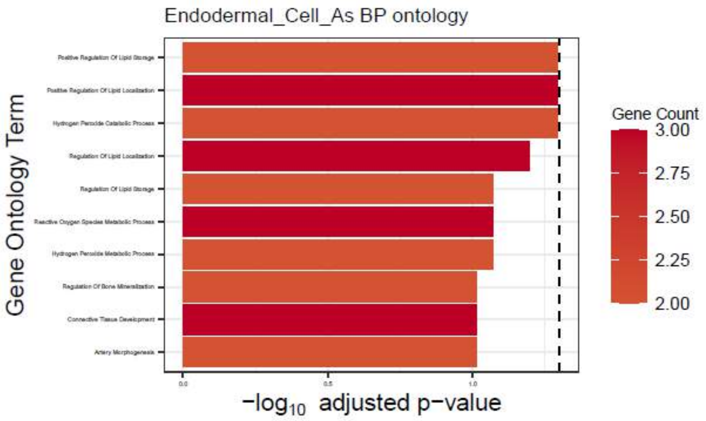
Endodermal_Cell_As BP ontology
Gene Ontology Term
Positive Regulation Of Lipid Storage
Positive Regulation Of Lipid Localization
Hydrogen Peroxide Catabolic Process
Regulation Of Lipid Localization
Regulation Of Lipid Storage
Reactive Oxygen Species Metabolic Process
Hydrogen Peroxide Metabolic Process
Regulation Of Bone Mineralization
Connective Tissue Development
Artery Morphogenesis
$-\log_{10}$ adjusted p-value
Gene Count
3.00
2.75
2.50
2.25
2.00

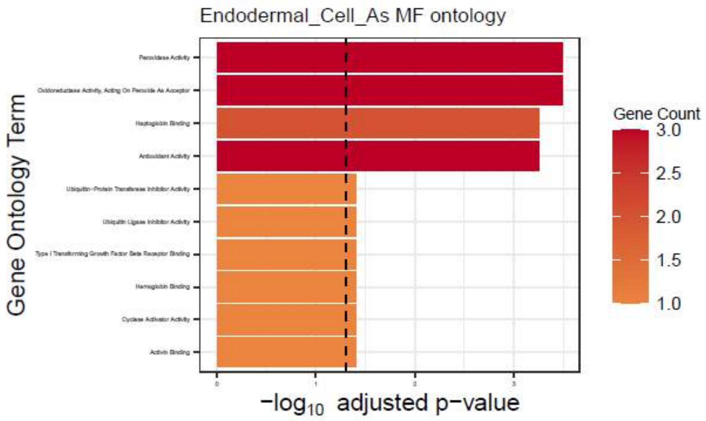
Endodermal_Cell_As MF ontology
Gene Ontology Term
Peroxidase Activity
Oxidoreductase Activity, Acting On Peroxide As Acceptor
Haptoglobin Binding
Antioxidant Activity
Ubiquitin-Protein Transferase Inhibitor Activity
Ubiquitin Ligase Inhibitor Activity
Type I Transforming Growth Factor Beta Receptor Binding
Hemoglobin Binding
Cyclase Activator Activity
Activin Binding
$-\log_{10}$ adjusted p-value
Gene Count
3.0
2.5
2.0
1.5
1.0

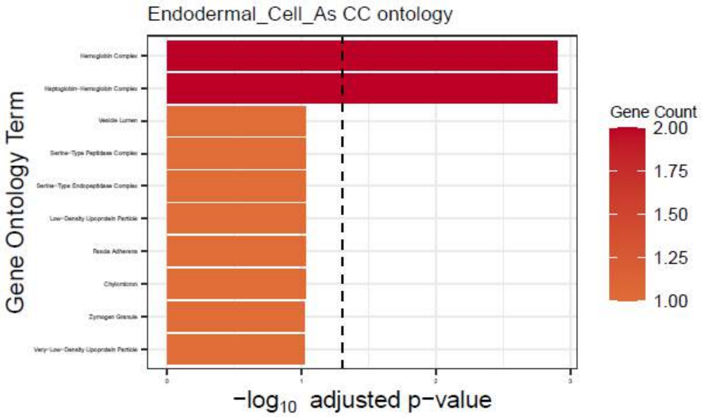
Endodermal_Cell_As CC ontology
Gene Ontology Term
Hemoglobin Complex
Haptoglobin–Hemoglobin Complex
Vesicle Lumen
Serine–Type Peptidase Complex
Serine–Type Endopeptidase Complex
Low–Density Lipoprotein Particle
Fascia Adherens
Chylomicron
Zymogen Granule
Very–Low–Density Lipoprotein Particle
0
1
2
3
$-\log_{10}$ adjusted p−value
Gene Count
2.00
1.75
1.50
1.25
1.00

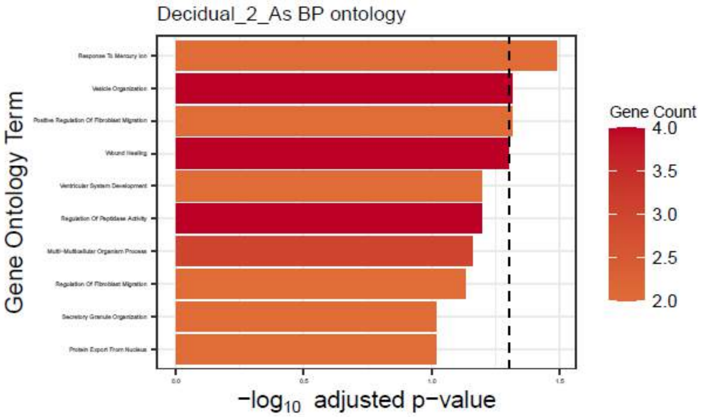
Decidual_2_As BP ontology
Gene Ontology Term
Response To Mercury Ion
Vesicle Organization
Positive Regulation Of Fibroblast Migration
Wound Healing
Ventricular System Development
Regulation Of Peptidase Activity
Multi-Multicellular Organism Process
Regulation Of Fibroblast Migration
Secretory Granule Organization
Protein Export From Nucleus
0.0
0.5
1.0
1.5
$-\log_{10}$ adjusted p−value
Gene Count
4.0
3.5
3.0
2.5
2.0

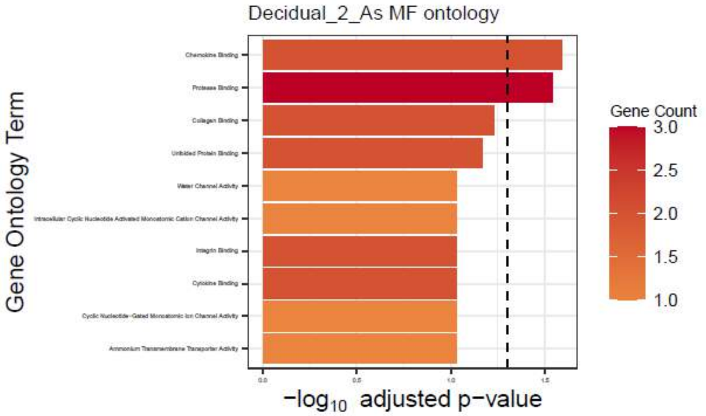
Decidual_2_As MF ontology
Gene Ontology Term
Chemokine Binding
Protease Binding
Collagen Binding
Unfolded Protein Binding
Water Channel Activity
Intracellular Cyclic Nucleotide Activated Monoatomic Cation Channel Activity
Integrin Binding
Cytokine Binding
Cyclic Nucleotide–Gated Monoatomic Ion Channel Activity
Ammonium Transmembrane Transporter Activity
0.0
0.5
1.0
1.5
$-\log_{10}$ adjusted p−value
Gene Count
3.0
2.5
2.0
1.5
1.0

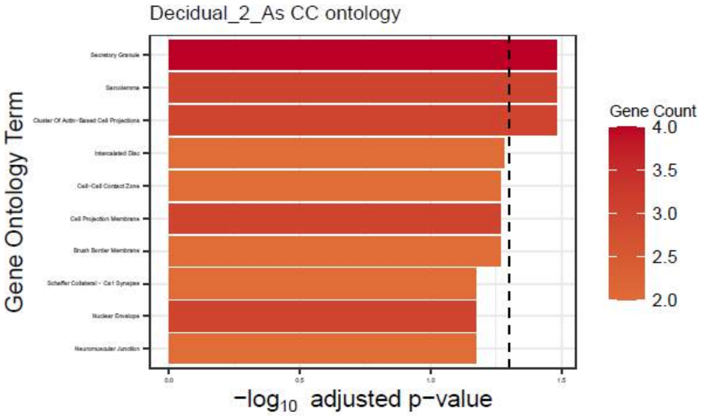
Decidual_2_As CC ontology
Gene Ontology Term
Secretory Granule
Sarcolemma
Cluster Of Actin–Based Cell Projections
Intercalated Disc
Cell–Cell Contact Zone
Cell Projection Membrane
Brush Border Membrane
Schaffer Collateral – Ca1 Synapse
Nuclear Envelope
Neuromuscular Junction
0.0
0.5
1.0
1.5
$-\log_{10}$ adjusted p−value
Gene Count
4.0
3.5
3.0
2.5
2.0

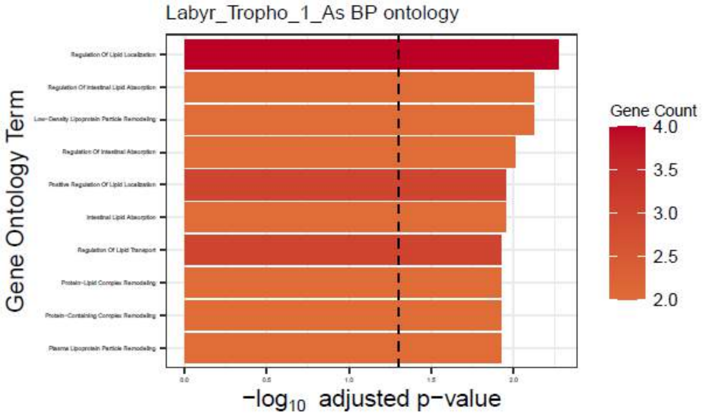
Labyr_Tropho_1_As BP ontology
Gene Ontology Term
Regulation Of Lipid Localization
Regulation Of Intestinal Lipid Absorption
Low–Density Lipoprotein Particle Remodeling
Regulation Of Intestinal Absorption
Positive Regulation Of Lipid Localization
Intestinal Lipid Absorption
Regulation Of Lipid Transport
Protein–Lipid Complex Remodeling
Protein–Containing Complex Remodeling
Plasma Lipoprotein Particle Remodeling
0.0
0.5
1.0
1.5
2.0
$-\log_{10}$ adjusted p−value
Gene Count
4.0
3.5
3.0
2.5
2.0

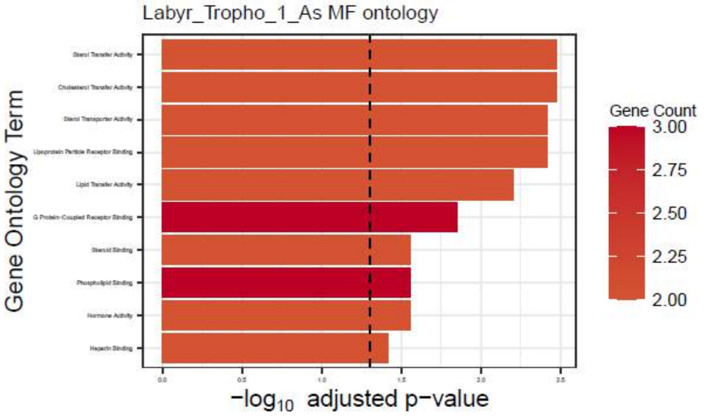
Labyr_Tropho_1_As MF ontology
Gene Ontology Term
Sterol Transfer Activity
Cholesterol Transfer Activity
Sterol Transporter Activity
Lipoprotein Particle Receptor Binding
Lipid Transfer Activity
G Protein–Coupled Receptor Binding
Steroid Binding
Phospholipid Binding
Hormone Activity
Heparin Binding
0.0
0.5
1.0
1.5
2.0
2.5
$-\log_{10}$ adjusted p−value
Gene Count
3.00
2.75
2.50
2.25
2.00

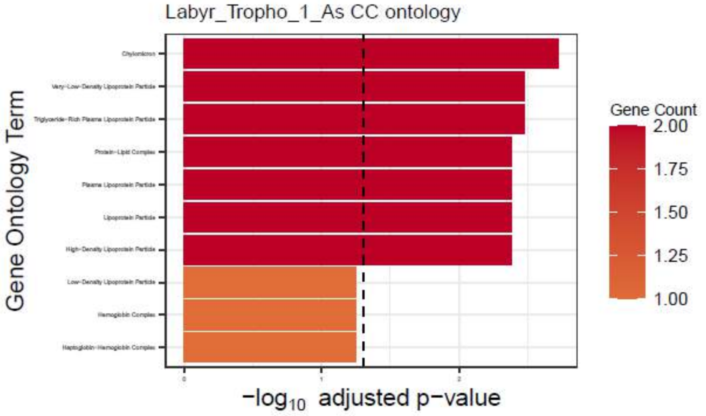
Labyr_Tropho_1_As CC ontology
Gene Ontology Term
Chylomicron
Very–Low–Density Lipoprotein Particle
Triglyceride–Rich Plasma Lipoprotein Particle
Protein–Lipid Complex
Plasma Lipoprotein Particle
Lipoprotein Particle
High–Density Lipoprotein Particle
Low–Density Lipoprotein Particle
Hemoglobin Complex
Haptoglobin–Hemoglobin Complex
0
1
2
$-\log_{10}$ adjusted p−value
Gene Count
2.00
1.75
1.50
1.25
1.00

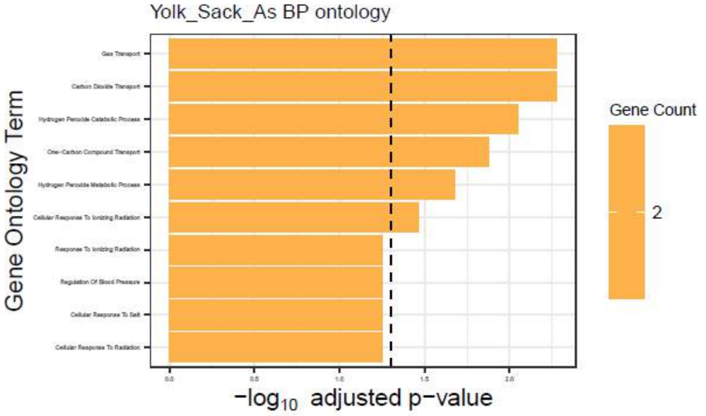
Yolk_Sack_As BP ontology
Gene Ontology Term
Gas Transport
Carbon Dioxide Transport
Hydrogen Peroxide Catabolic Process
One–Carbon Compound Transport
Hydrogen Peroxide Metabolic Process
Cellular Response To Ionizing Radiation
Response To Ionizing Radiation
Regulation Of Blood Pressure
Cellular Response To Salt
Cellular Response To Radiation
0.0
0.5
1.0
1.5
2.0
$-\log_{10}$ adjusted p−value
Gene Count
2

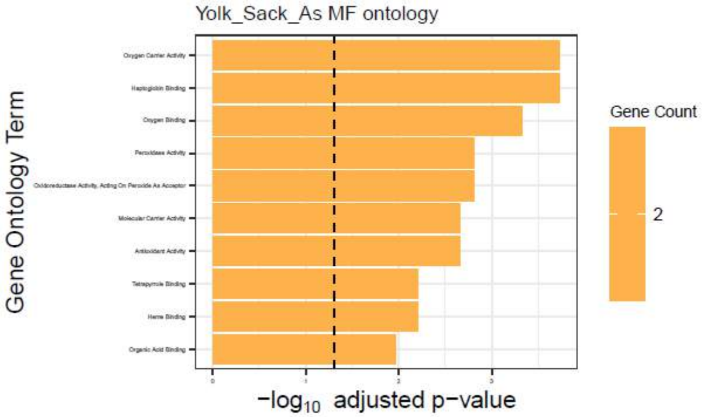

Yolk_Sack_As MF ontology
Gene Ontology Term
Oxygen Carrier Activity
Haptoglobin Binding
Oxygen Binding
Peroxidase Activity
Oxidoreductase Activity, Acting On Peroxide As Acceptor
Molecular Carrier Activity
Antioxidant Activity
Tetrapyrrole Binding
Heme Binding
Organic Acid Binding
−log10 adjusted p−value
Gene Count
2

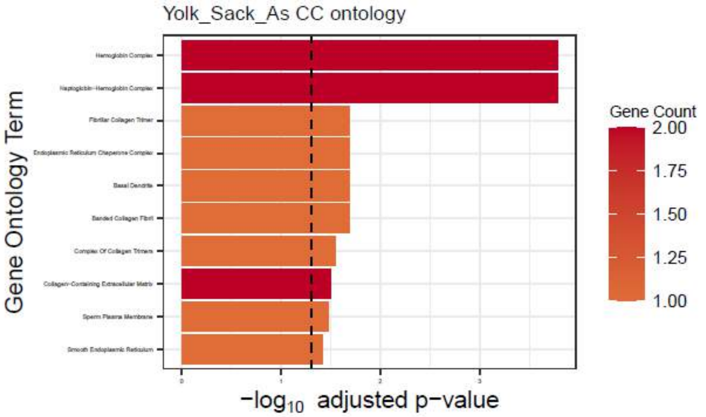

Yolk_Sack_As CC ontology
Gene Ontology Term
Hemoglobin Complex
Haptoglobin−Hemoglobin Complex
Fibrillar Collagen Trimer
Endoplasmic Reticulum Chaperone Complex
Basal Dendrite
Banded Collagen Fibril
Complex Of Collagen Trimers
Collagen−Containing Extracellular Matrix
Sperm Plasma Membrane
Smooth Endoplasmic Reticulum
−log10 adjusted p−value
Gene Count
2.00
1.75
1.50
1.25
1.00

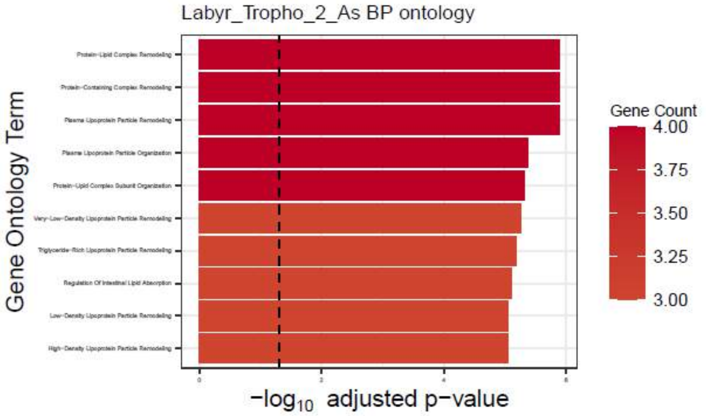

Labyr_Tropho_2_As BP ontology
Gene Ontology Term
Protein−Lipid Complex Remodeling
Protein−Containing Complex Remodeling
Plasma Lipoprotein Particle Remodeling
Plasma Lipoprotein Particle Organization
Protein−Lipid Complex Subunit Organization
Very−Low−Density Lipoprotein Particle Remodeling
Triglyceride−Rich Lipoprotein Particle Remodeling
Regulation Of Intestinal Lipid Absorption
Low−Density Lipoprotein Particle Remodeling
High−Density Lipoprotein Particle Remodeling
−log10 adjusted p−value
Gene Count
4.00
3.75
3.50
3.25
3.00

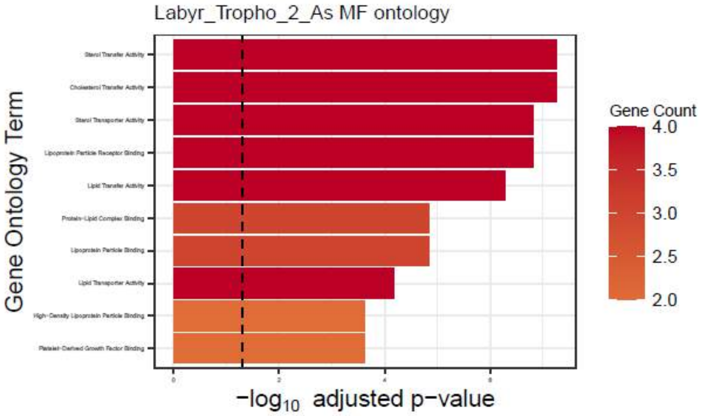

Labyr_Tropho_2_As MF ontology
Gene Ontology Term
Sterol Transfer Activity
Cholesterol Transfer Activity
Sterol Transporter Activity
Lipoprotein Particle Receptor Binding
Lipid Transfer Activity
Protein−Lipid Complex Binding
Lipoprotein Particle Binding
Lipid Transporter Activity
High−Density Lipoprotein Particle Binding
Platelet−Derived Growth Factor Binding
−log10 adjusted p−value
Gene Count
4.0
3.5
3.0
2.5
2.0

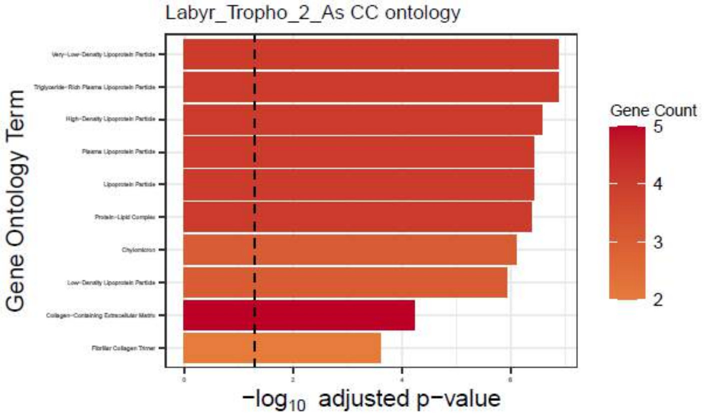

Labyr_Tropho_2_As CC ontology
Gene Ontology Term
Very−Low−Density Lipoprotein Particle
Triglyceride−Rich Plasma Lipoprotein Particle
High−Density Lipoprotein Particle
Plasma Lipoprotein Particle
Lipoprotein Particle
Protein−Lipid Complex
Chylomicron
Low−Density Lipoprotein Particle
Collagen−Containing Extracellular Matrix
Fibrillar Collagen Trimer
−log10 adjusted p−value
Gene Count
5
4
3
2

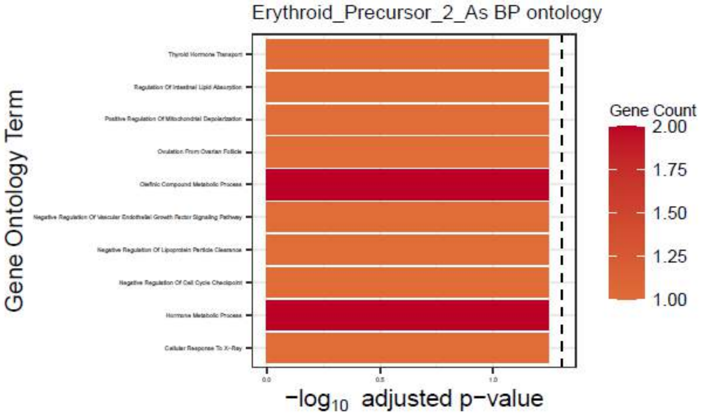

Erythroid_Precursor_2_As BP ontology
Gene Ontology Term
Thyroid Hormone Transport
Regulation Of Intestinal Lipid Absorption
Positive Regulation Of Mitochondrial Depolarization
Ovulation From Ovarian Follicle
Olefinic Compound Metabolic Process
Negative Regulation Of Vascular Endothelial Growth Factor Signaling Pathway
Negative Regulation Of Lipoprotein Particle Clearance
Negative Regulation Of Cell Cycle Checkpoint
Hormone Metabolic Process
Cellular Response To X−Ray
−log10 adjusted p−value
Gene Count
2.00
1.75
1.50
1.25
1.00

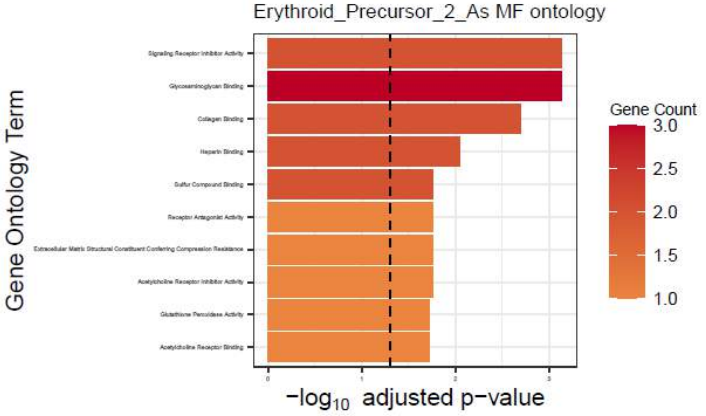

Erythroid_Precursor_2_As MF ontology
Gene Ontology Term
Signaling Receptor Inhibitor Activity
Glycosaminoglycan Binding
Collagen Binding
Heparin Binding
Sulfur Compound Binding
Receptor Antagonist Activity
Extracellular Matrix Structural Constituent Conferring Compression Resistance
Acetylcholine Receptor Inhibitor Activity
Glutathione Peroxidase Activity
Acetylcholine Receptor Binding
−log10 adjusted p−value
Gene Count
3.0
2.5
2.0
1.5
1.0

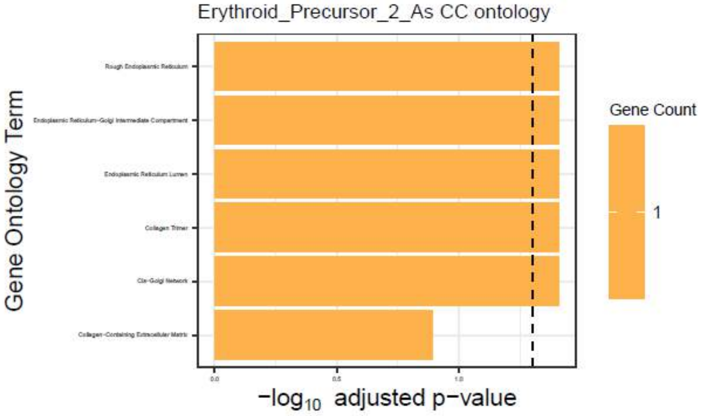

Erythroid_Precursor_2_As CC ontology
Gene Ontology Term
Rough Endoplasmic Reticulum
Endoplasmic Reticulum−Golgi Intermediate Compartment
Endoplasmic Reticulum Lumen
Collagen Trimer
Cis−Golgi Network
Collagen−Containing Extracellular Matrix
−log10 adjusted p−value
Gene Count
1

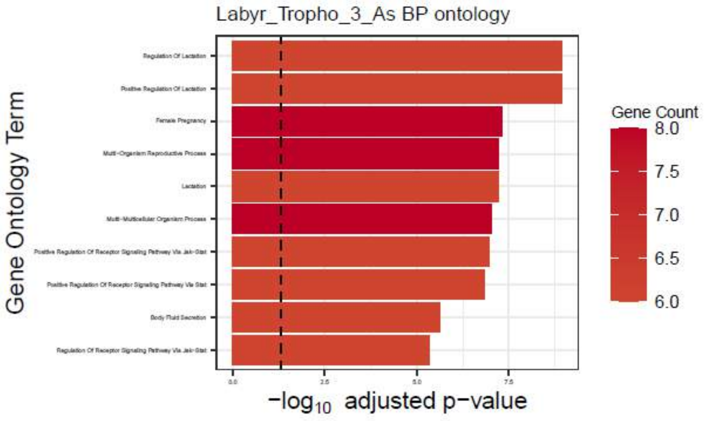
Labyr_Tropho_3_As BP ontology
Gene Ontology Term
Gene Count
8.0
7.5
7.0
6.5
6.0
−log10 adjusted p−value

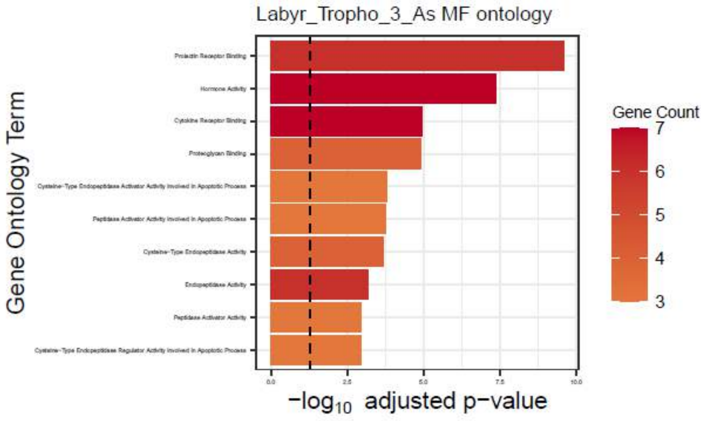
Labyr_Tropho_3_As MF ontology
Gene Ontology Term
Gene Count
7
6
5
4
3
−log10 adjusted p−value

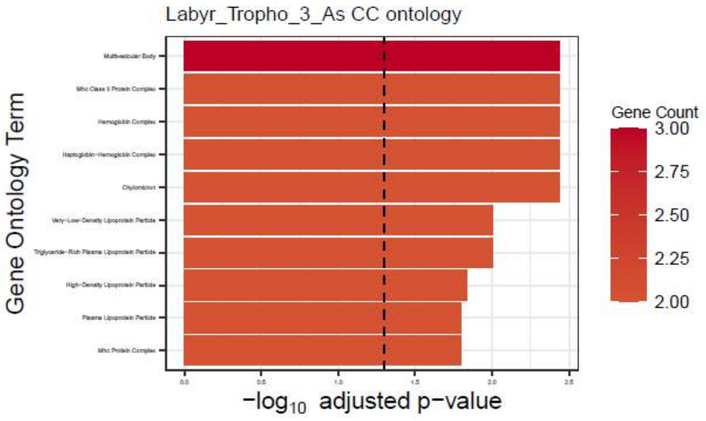
Labyr_Tropho_3_As CC ontology
Gene Ontology Term
Gene Count
3.00
2.75
2.50
2.25
2.00
−log10 adjusted p−value

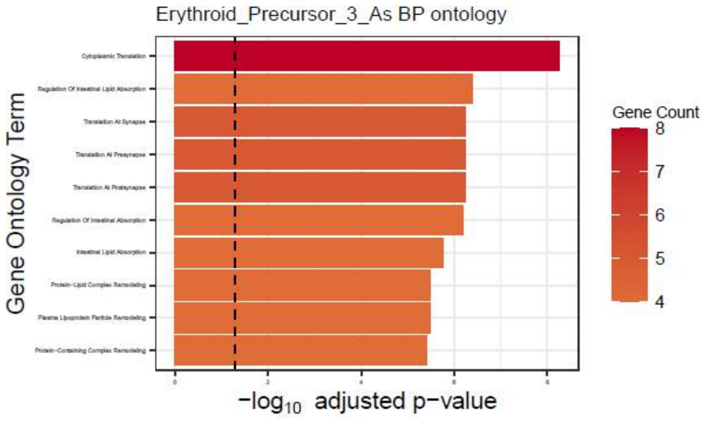
Erythroid_Precursor_3_As BP ontology
Gene Ontology Term
Gene Count
8
7
6
5
4
−log10 adjusted p−value

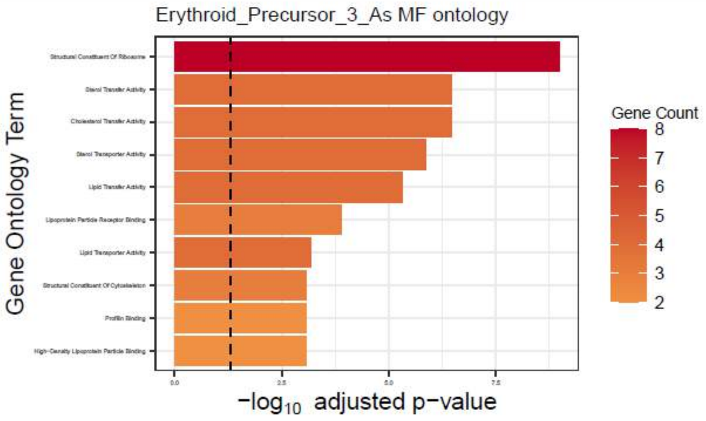
Erythroid_Precursor_3_As MF ontology
Gene Ontology Term
Gene Count
8
7
6
5
4
3
2
−log10 adjusted p−value

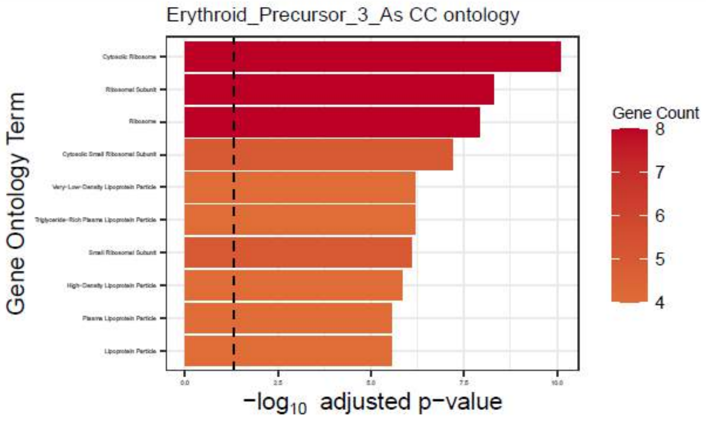
Erythroid_Precursor_3_As CC ontology
Gene Ontology Term
Gene Count
8
7
6
5
4
−log10 adjusted p−value

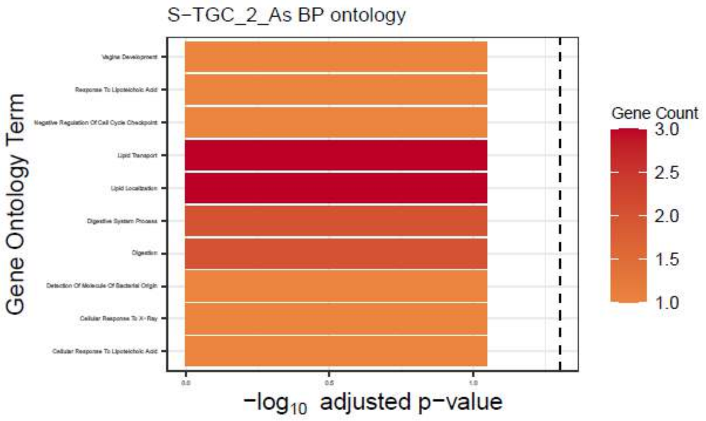
S−TGC_2_As BP ontology
Gene Ontology Term
Gene Count
3.0
2.5
2.0
1.5
1.0
−log10 adjusted p−value

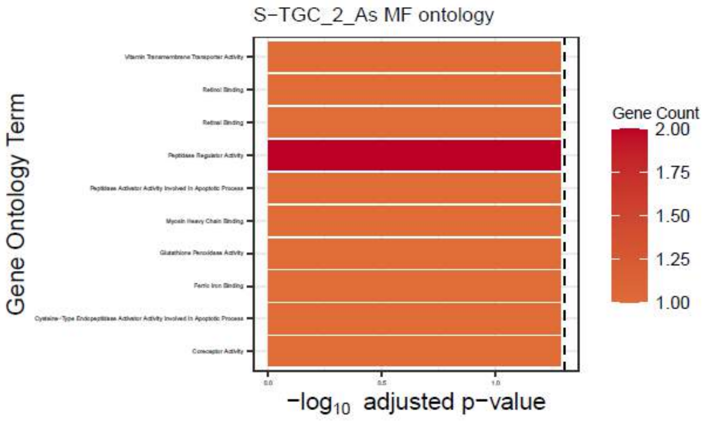
S−TGC_2_As MF ontology
Gene Ontology Term
Gene Count
2.00
1.75
1.50
1.25
1.00
−log10 adjusted p−value

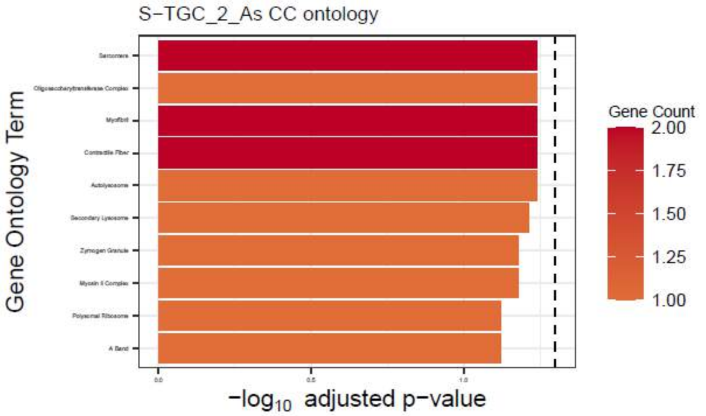
S−TGC_2_As CC ontology
Gene Ontology Term
−log10 adjusted p−value
Gene Count
2.00
1.75
1.50
1.25
1.00

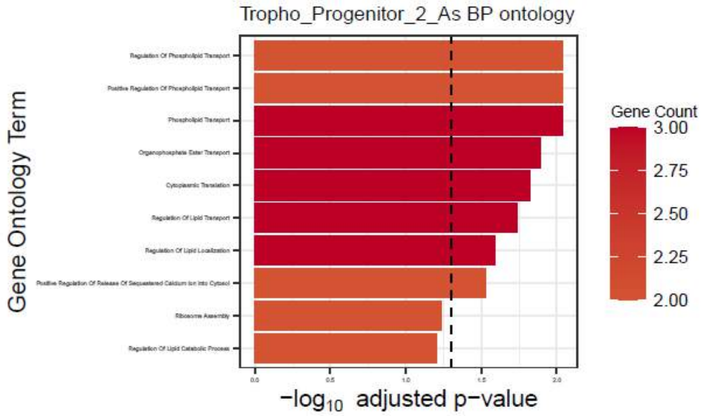
Tropho_Progenitor_2_As BP ontology
Gene Ontology Term
−log10 adjusted p−value
Gene Count
3.00
2.75
2.50
2.25
2.00

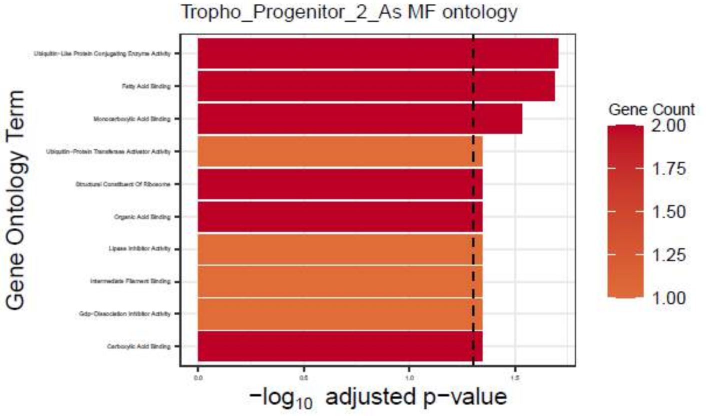
Tropho_Progenitor_2_As MF ontology
Gene Ontology Term
−log10 adjusted p−value
Gene Count
2.00
1.75
1.50
1.25
1.00

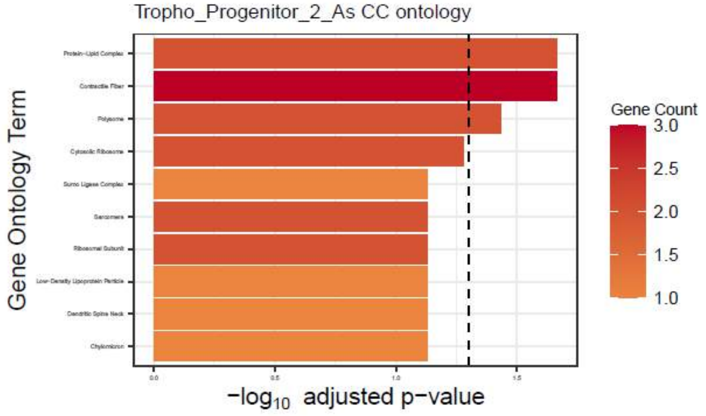
Tropho_Progenitor_2_As CC ontology
Gene Ontology Term
−log10 adjusted p−value
Gene Count
3.0
2.5
2.0
1.5
1.0

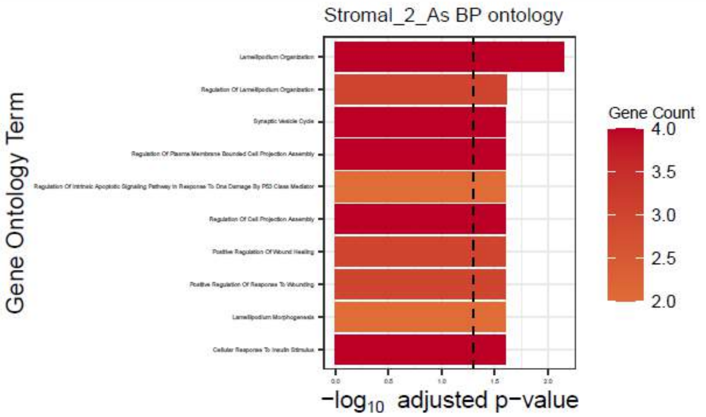
Stromal_2_As BP ontology
Gene Ontology Term
−log10 adjusted p−value
Gene Count
4.0
3.5
3.0
2.5
2.0

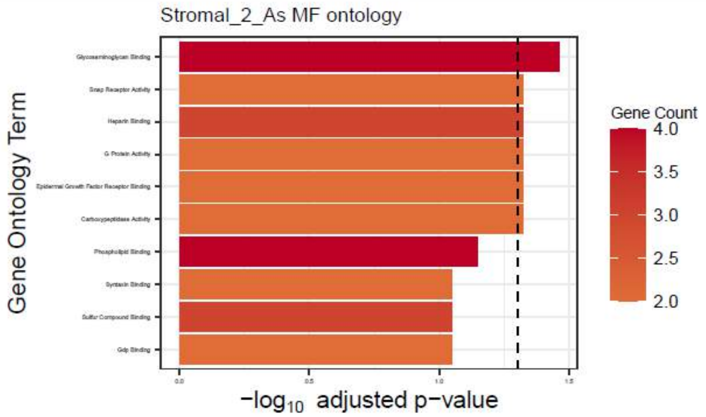
Stromal_2_As MF ontology
Gene Ontology Term
−log10 adjusted p−value
Gene Count
4.0
3.5
3.0
2.5
2.0

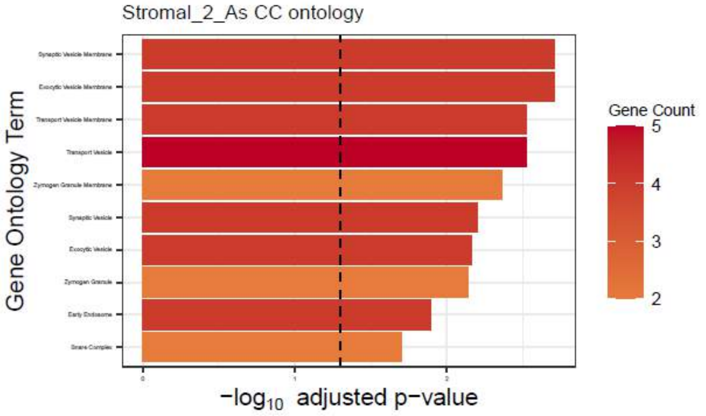
Stromal_2_As CC ontology
Gene Ontology Term
−log10 adjusted p−value
Gene Count
5
4
3
2

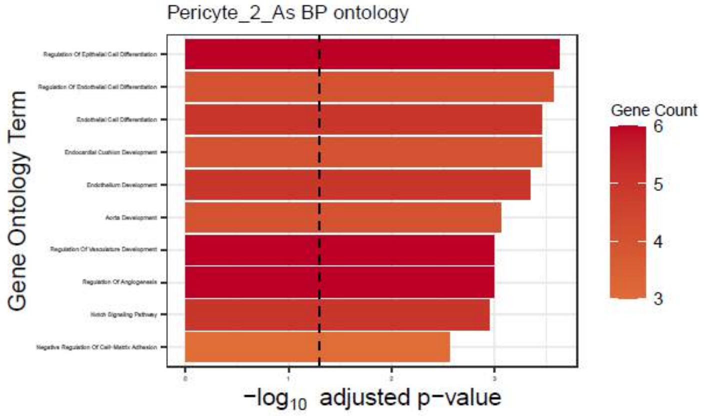
Pericyte_2_As BP ontology
Gene Ontology Term
−log10 adjusted p−value
Gene Count
6
5
4
3

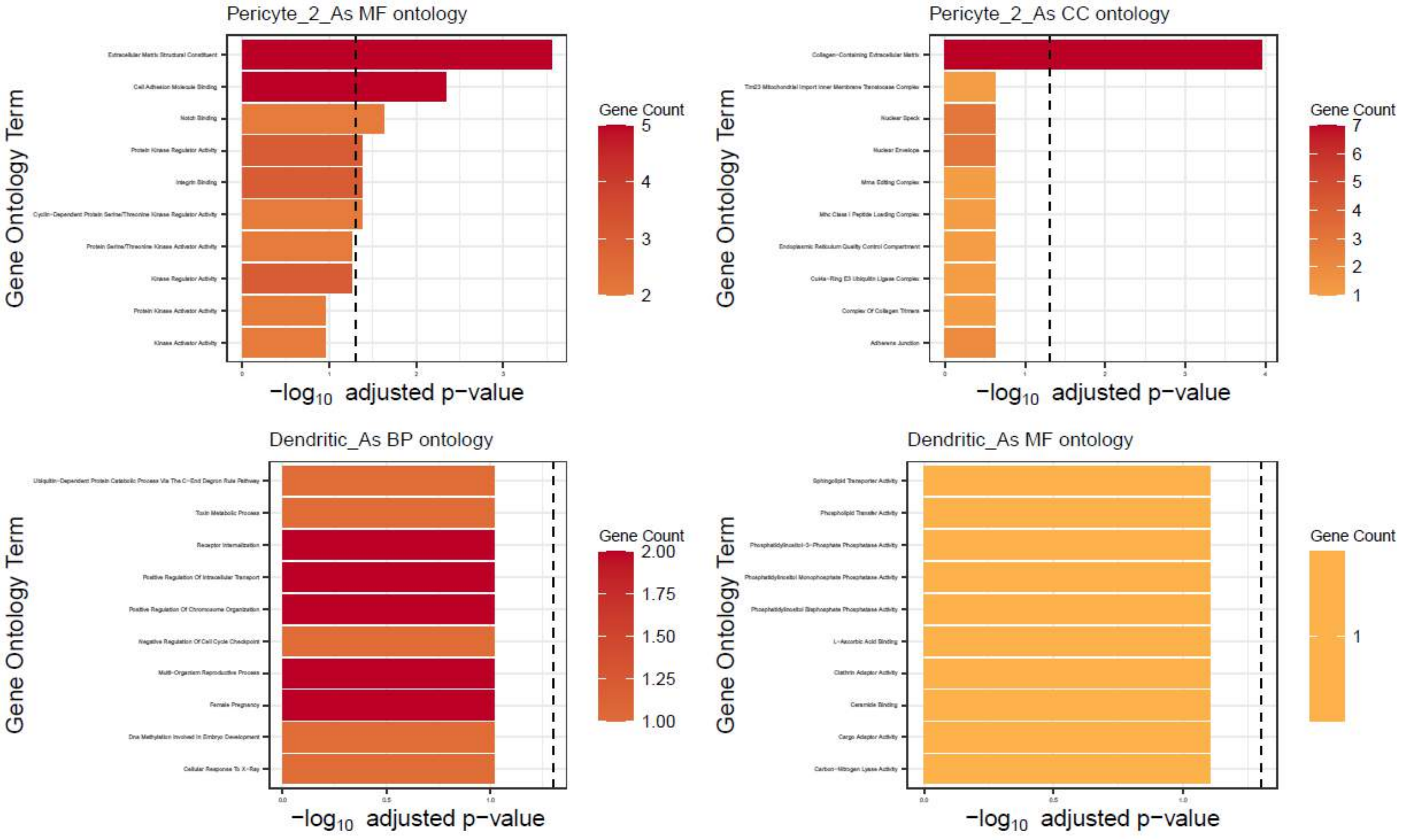
Pericyte_2_As MF ontology
Gene Ontology Term
Extracellular Matrix Structural Constituent
Cell Adhesion Molecule Binding
Notch Binding
Protein Kinase Regulator Activity
Integrin Binding
Cyclin-Dependent Protein Serine/Threonine Kinase Regulator Activity
Protein Serine/Threonine Kinase Activator Activity
Kinase Regulator Activity
Protein Kinase Activator Activity
Kinase Activator Activity
−log10 adjusted p−value
Gene Count
5
4
3
2
Pericyte_2_As CC ontology
Gene Ontology Term
Collagen−Containing Extracellular Matrix
Tim23 Mitochondrial Import Inner Membrane Translocase Complex
Nuclear Speck
Nuclear Envelope
Mrna Editing Complex
Mhc Class I Peptide Loading Complex
Endoplasmic Reticulum Quality Control Compartment
Cullin−Ring E3 Ubiquitin Ligase Complex
Complex Of Collagen Trimers
Adherens Junction
−log10 adjusted p−value
Gene Count
7
6
5
4
3
2
1
Dendritic_As BP ontology
Gene Ontology Term
Ubiquitin−Dependent Protein Catabolic Process Via The C−End Degron Rule Pathway
Toxin Metabolic Process
Receptor Internalization
Positive Regulation Of Intracellular Transport
Positive Regulation Of Chromosome Organization
Negative Regulation Of Cell Cycle Checkpoint
Multi-Organism Reproductive Process
Female Pregnancy
Dna Methylation Involved In Embryo Development
Cellular Response To X−Ray
−log10 adjusted p−value
Gene Count
2.00
1.75
1.50
1.25
1.00
Dendritic_As MF ontology
Gene Ontology Term
Sphingolipid Transporter Activity
Phospholipid Transfer Activity
Phosphatidylinositol-3-Phosphate Phosphatase Activity
Phosphatidylinositol Monophosphate Phosphatase Activity
Phosphatidylinositol Bisphosphate Phosphatase Activity
L−Ascorbic Acid Binding
Clathrin Adaptor Activity
Ceramide Binding
Cargo Adaptor Activity
Carbon−Nitrogen Lyase Activity
−log10 adjusted p−value
Gene Count
1

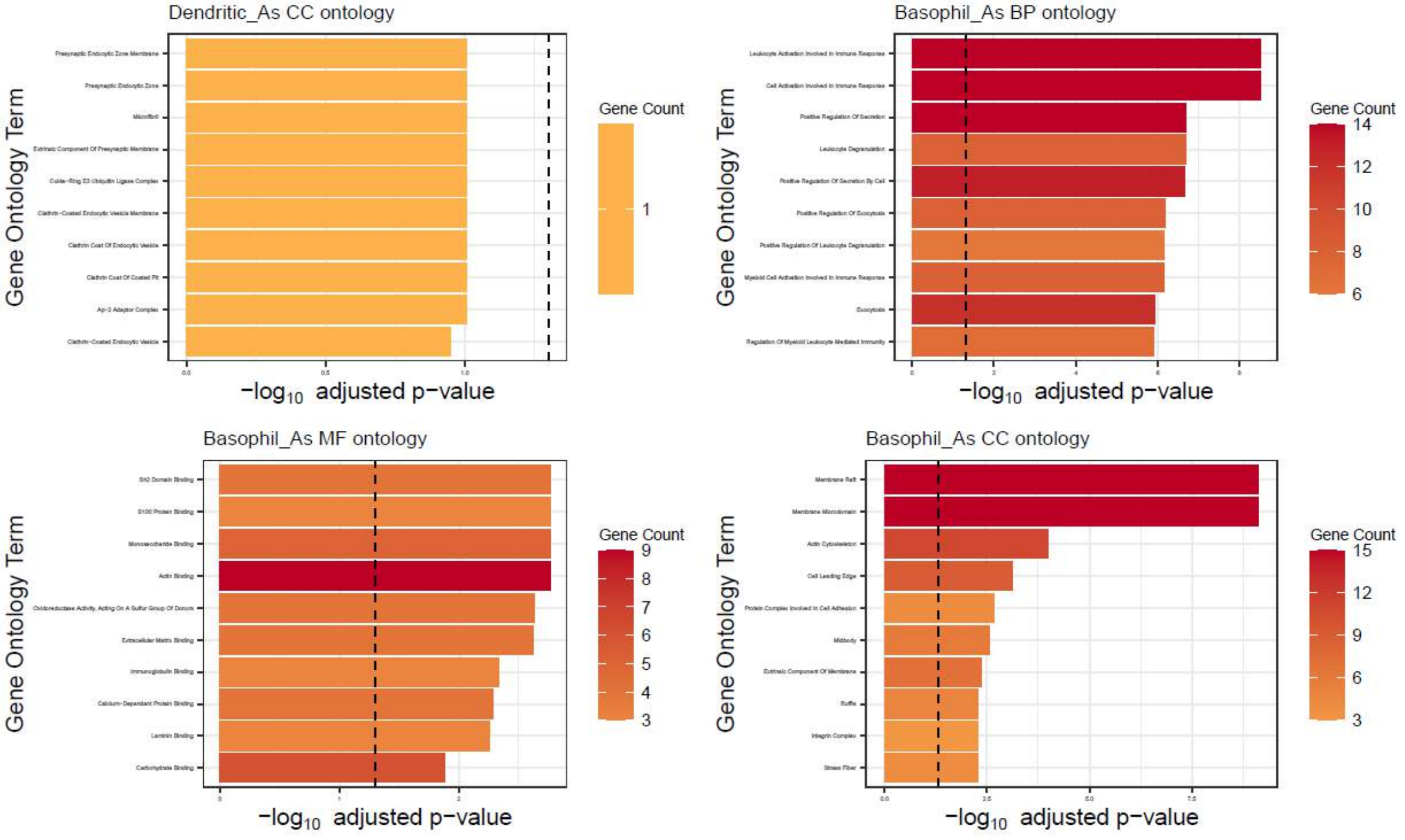
Dendritic_As CC ontology
Gene Ontology Term
Presynaptic Endocytic Zone Membrane
Presynaptic Endocytic Zone
Microfibril
Extrinsic Component Of Presynaptic Membrane
Cullin−Ring E3 Ubiquitin Ligase Complex
Clathrin−Coated Endocytic Vesicle Membrane
Clathrin Coat Of Endocytic Vesicle
Clathrin Coat Of Coated Pit
Ap-2 Adaptor Complex
Clathrin−Coated Endocytic Vesicle
−log10 adjusted p−value
Gene Count
1
Basophil_As BP ontology
Gene Ontology Term
Leukocyte Activation Involved In Immune Response
Cell Activation Involved In Immune Response
Positive Regulation Of Secretion
Leukocyte Degranulation
Positive Regulation Of Secretion By Cell
Positive Regulation Of Exocytosis
Positive Regulation Of Leukocyte Degranulation
Myeloid Cell Activation Involved In Immune Response
Exocytosis
Regulation Of Myeloid Leukocyte Mediated Immunity
−log10 adjusted p−value
Gene Count
14
12
10
8
6
Basophil_As MF ontology
Gene Ontology Term
Sh2 Domain Binding
S100 Protein Binding
Monosaccharide Binding
Actin Binding
Oxidoreductase Activity, Acting On A Sulfur Group Of Donors
Extracellular Matrix Binding
Immunoglobulin Binding
Calcium-Dependent Protein Binding
Laminin Binding
Carbohydrate Binding
−log10 adjusted p−value
Gene Count
9
8
7
6
5
4
3
Basophil_As CC ontology
Gene Ontology Term
Membrane Raft
Membrane Microdomain
Actin Cytoskeleton
Cell Leading Edge
Protein Complex Involved In Cell Adhesion
Midbody
Extrinsic Component Of Membrane
Ruffle
Integrin Complex
Stress Fiber
−log10 adjusted p−value
Gene Count
15
12
9
6
3

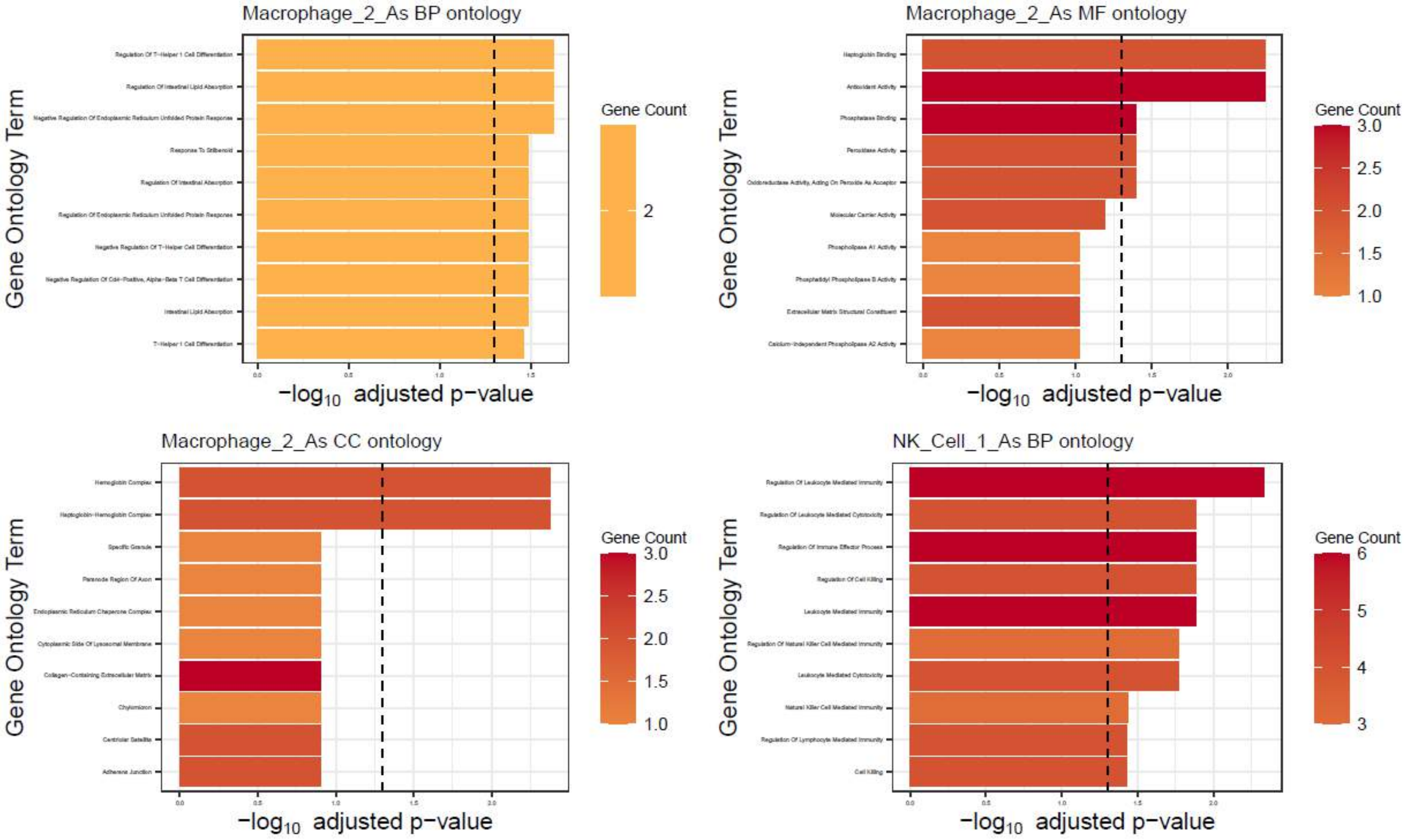
Macrophage_2_As BP ontology
Gene Ontology Term
−log10 adjusted p−value
Gene Count
2
Macrophage_2_As MF ontology
Gene Ontology Term
−log10 adjusted p−value
Gene Count
3.0
2.5
2.0
1.5
1.0
Macrophage_2_As CC ontology
Gene Ontology Term
−log10 adjusted p−value
Gene Count
3.0
2.5
2.0
1.5
1.0
NK_Cell_1_As BP ontology
Gene Ontology Term
−log10 adjusted p−value
Gene Count
6
5
4
3

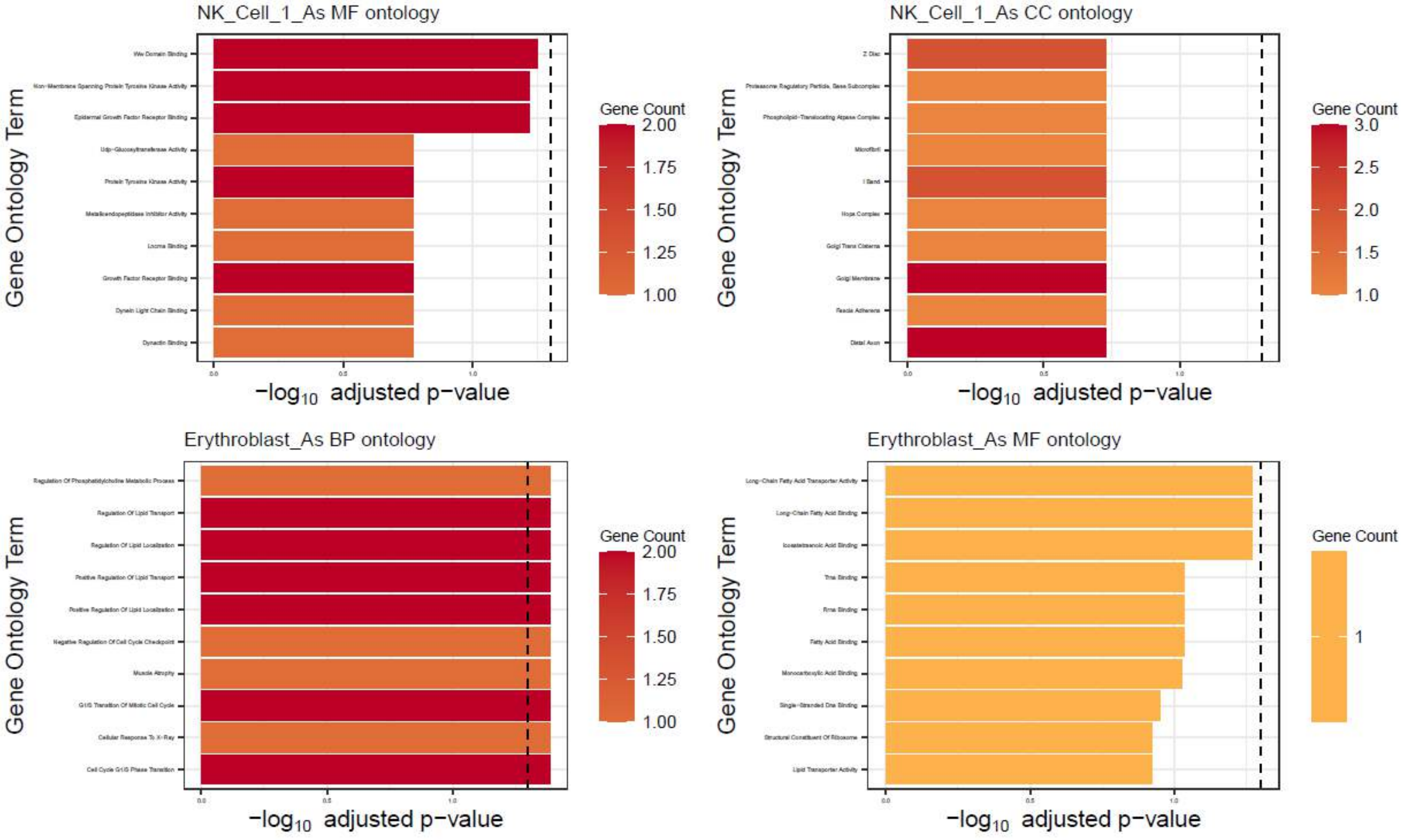
NK_Cell_1_As MF ontology
Gene Ontology Term
−log10 adjusted p−value
Gene Count
2.00
1.75
1.50
1.25
1.00
NK_Cell_1_As CC ontology
Gene Ontology Term
−log10 adjusted p−value
Gene Count
3.0
2.5
2.0
1.5
1.0
Erythroblast_As BP ontology
Gene Ontology Term
−log10 adjusted p−value
Gene Count
2.00
1.75
1.50
1.25
1.00
Erythroblast_As MF ontology
Gene Ontology Term
−log10 adjusted p−value
Gene Count
1

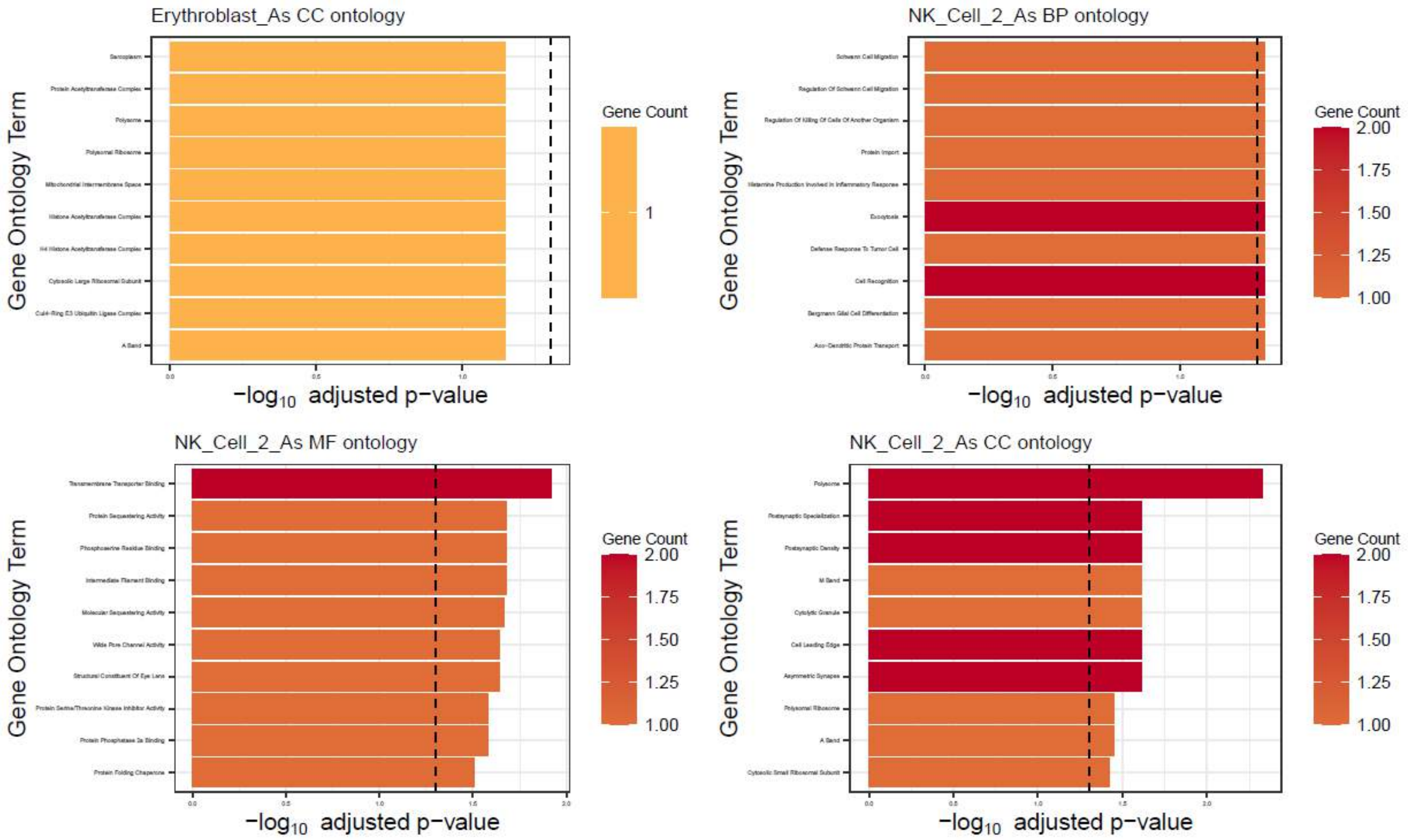

Supplementary Fig. 13. GO term analyses comparing As-exposed cells to non-exposed (control) cells. BP: Biological Processes; MF: Molecular Function; CC: Cellular Component. GO term analyses are based on the hypergeometric test, with two-sided p-values adjusting for multiple comparisons via the Benjamini-Hochberg method to control the False Discovery Rate.

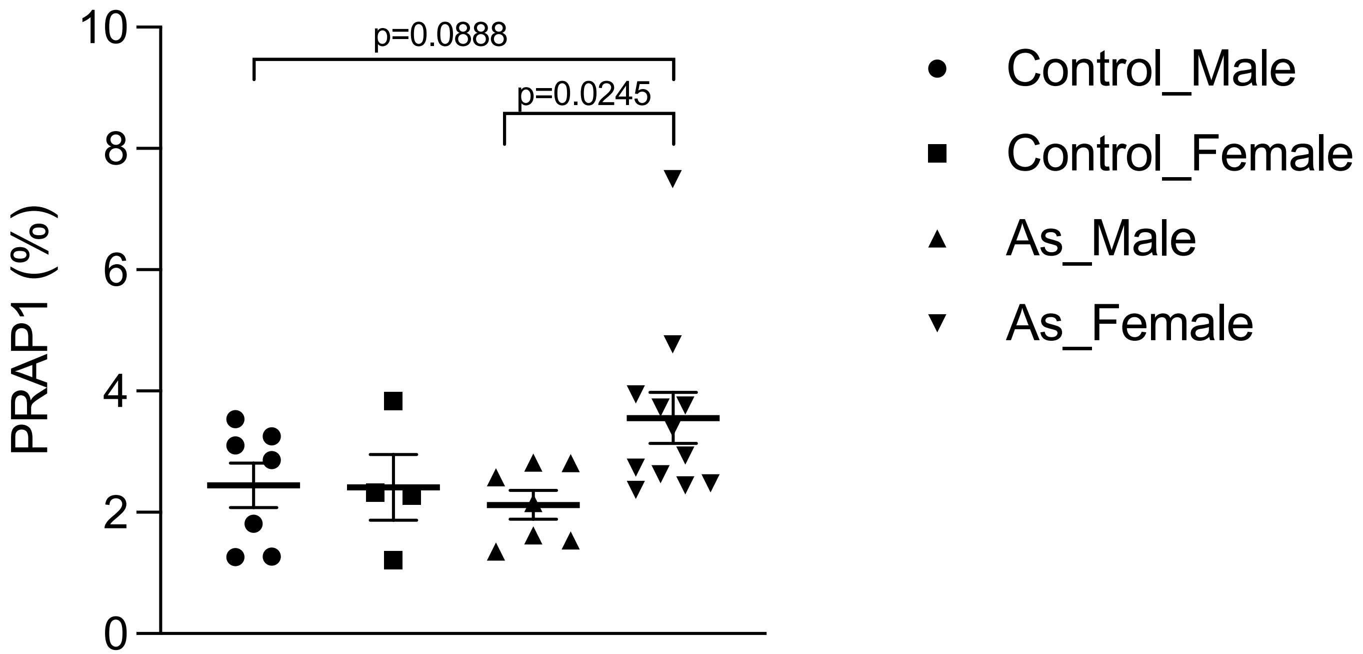

Supplementary Fig. 14. Semi-quantification of PRAP1 expression in the mouse placentae. n=7 placentae in Control_Male, 4 placentae in Control_Female, 7 placentae in As_Male, and 12 placentae in As_Female. As: Arsenic. Unpaired, two-tailed t-tests**.** Source data are provided as a Source Data file.

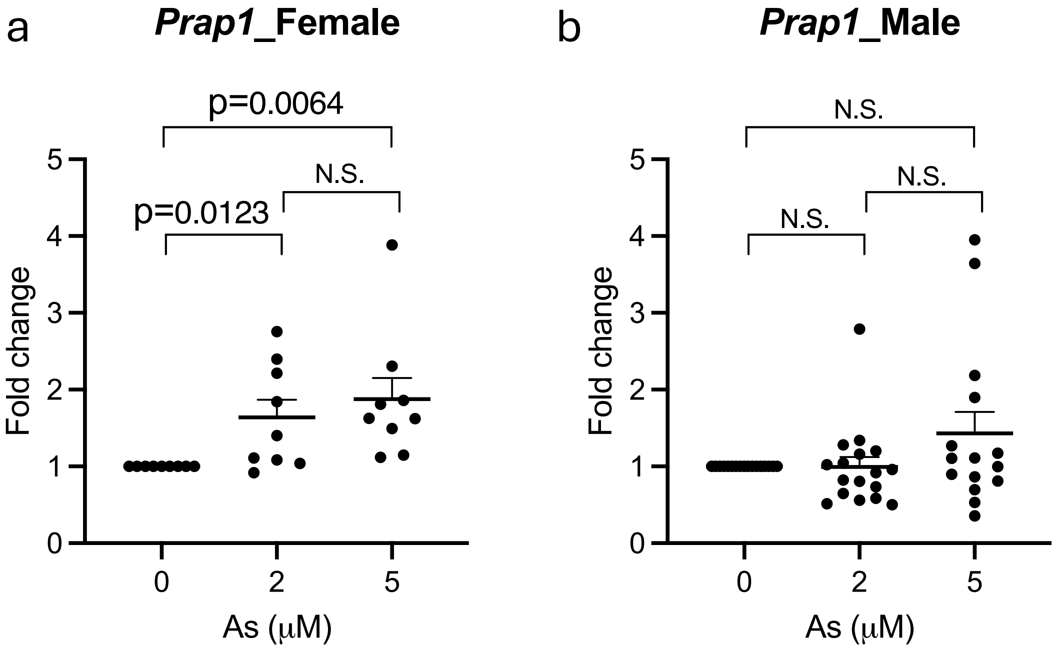

Supplementary Fig. 15. Expression of *Prap1* in mouse placental explants exposed to As. a. *Prap1* expression in female placentae (N=9 placentae). b. *Prap1* expression in male placentae (N=17 placentae). Placentae were collected from four pregnant mice at E 16.5 or 17.5. Unpaired, two-tailed t-tests. N.S, not significant. Source data are provided as a Source Data file.

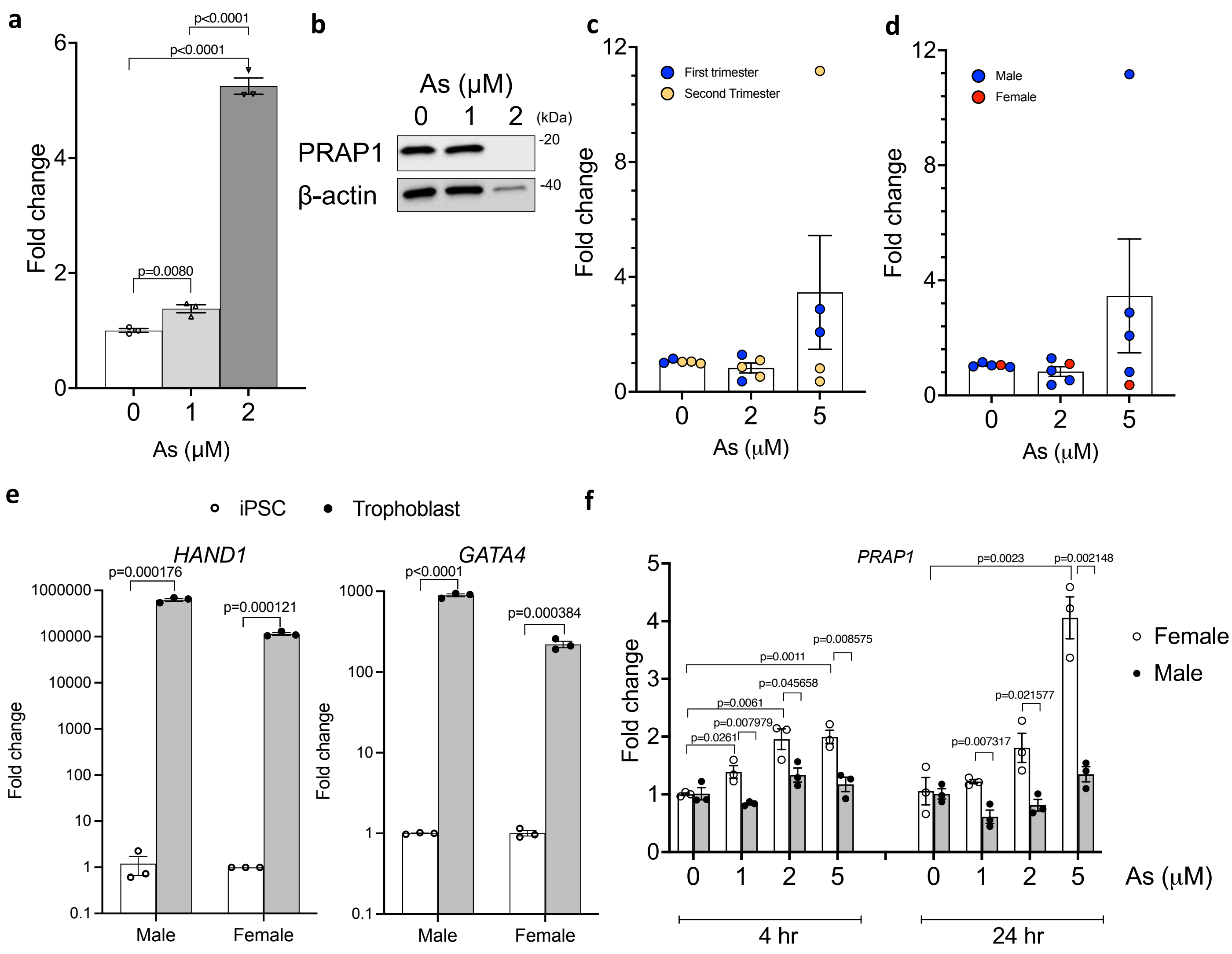

Supplementary Fig. 16. Validation of *PRAP1* expression in human placental cells and tissues. (a and b) Effect of chronic Arsenic treatment on PRAP1 expression in HTR-8/SVneo cells (n=3 cell culture plates in each group). (c and d) *Prap1* expression human placental villous explants exposed to Arsenic by trimester and by sex. (n= 5 placentae). (e) Expression of trophoblast markers in trophoblasts derived from male and female human iPSCs (n=3 cell culture plates in each group). (f) Expression of *PRAP1* in trophoblasts from male and female human iPSCs exposed to Arsenic for 4 or 24 hr (n=3 cell culture plates in each group).   As: Arsenic. Unpaired, two-tailed t-tests. Source data are provided as a Source Data file.

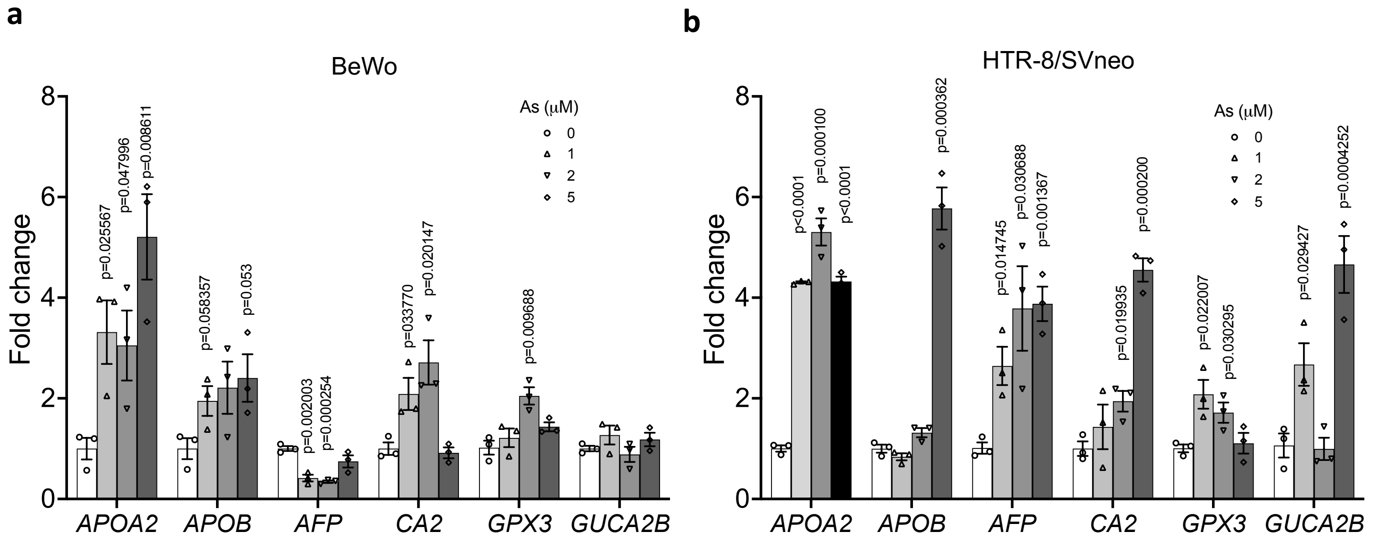

Supplementary Fig. 17. Validation of additional DE genes in (a) BeWo and (b) HTR-8/SVneo cells treated with Arsenic (n=3 cell culture plates in each group). As: Arsenic. Unpaired, two-tailed t-tests (compared to 0 μM As). Source data are provided as a Source Data file.

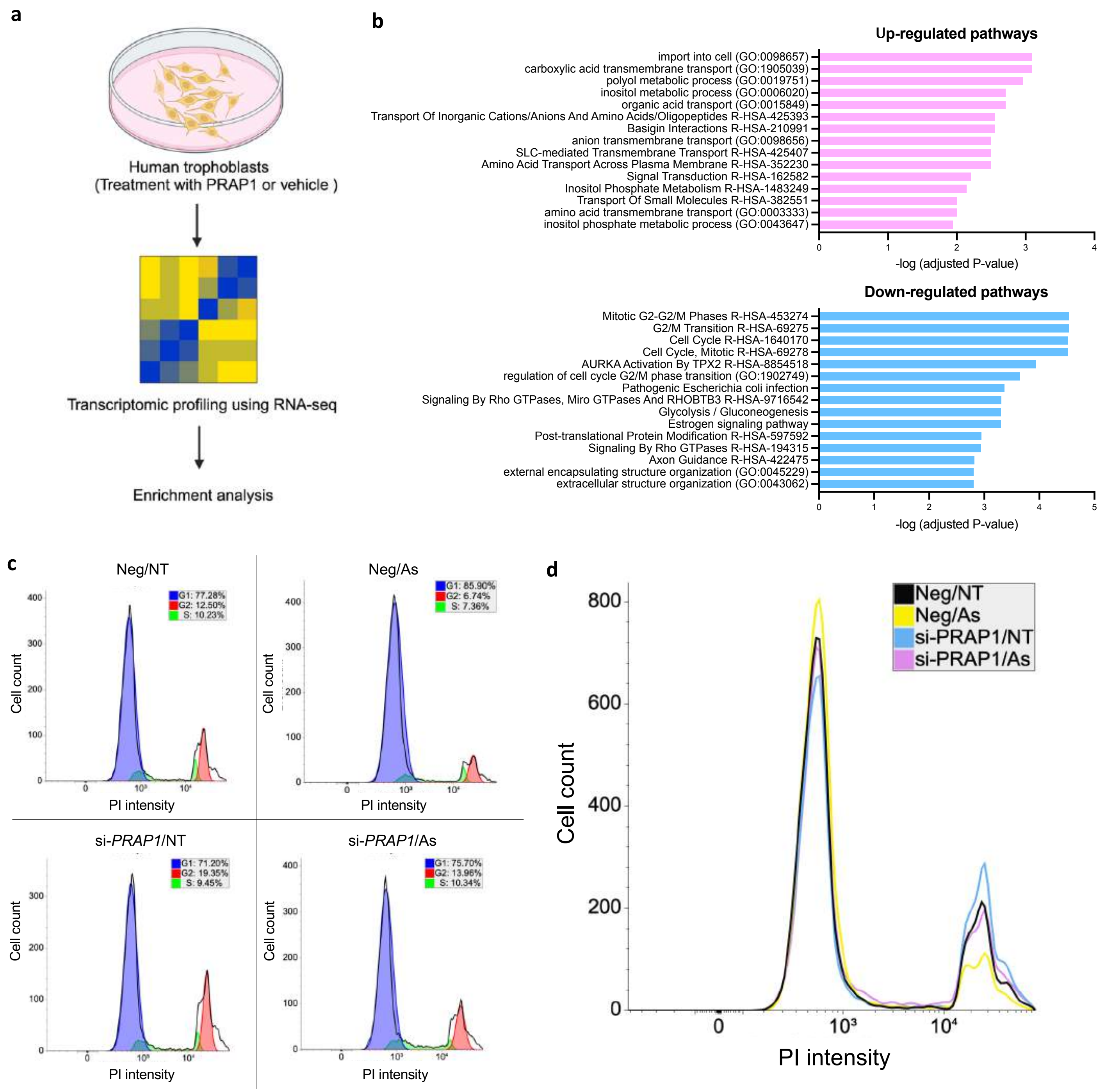

Supplementary Fig. 18. (a) Schematic workflow of RNA-seq and GO analysis on BeWo cells treated with PRAP1 protein. Created with BioRender.com released under a Creative Commons Attribution-NonCommercial-NoDerivs 4.0 International license (https://creativecommons.org/licenses/by-nc-nd/4.0/deed.en)' (b) Significantly enriched pathways with PRAP1 treatment. (c-d) Cell cycle analysis in control or PRAP1 knockdown cells treated with 0 (NT) or 2 μM Arsenic for 24 hr. NT: non-treated; As: Arsenic.

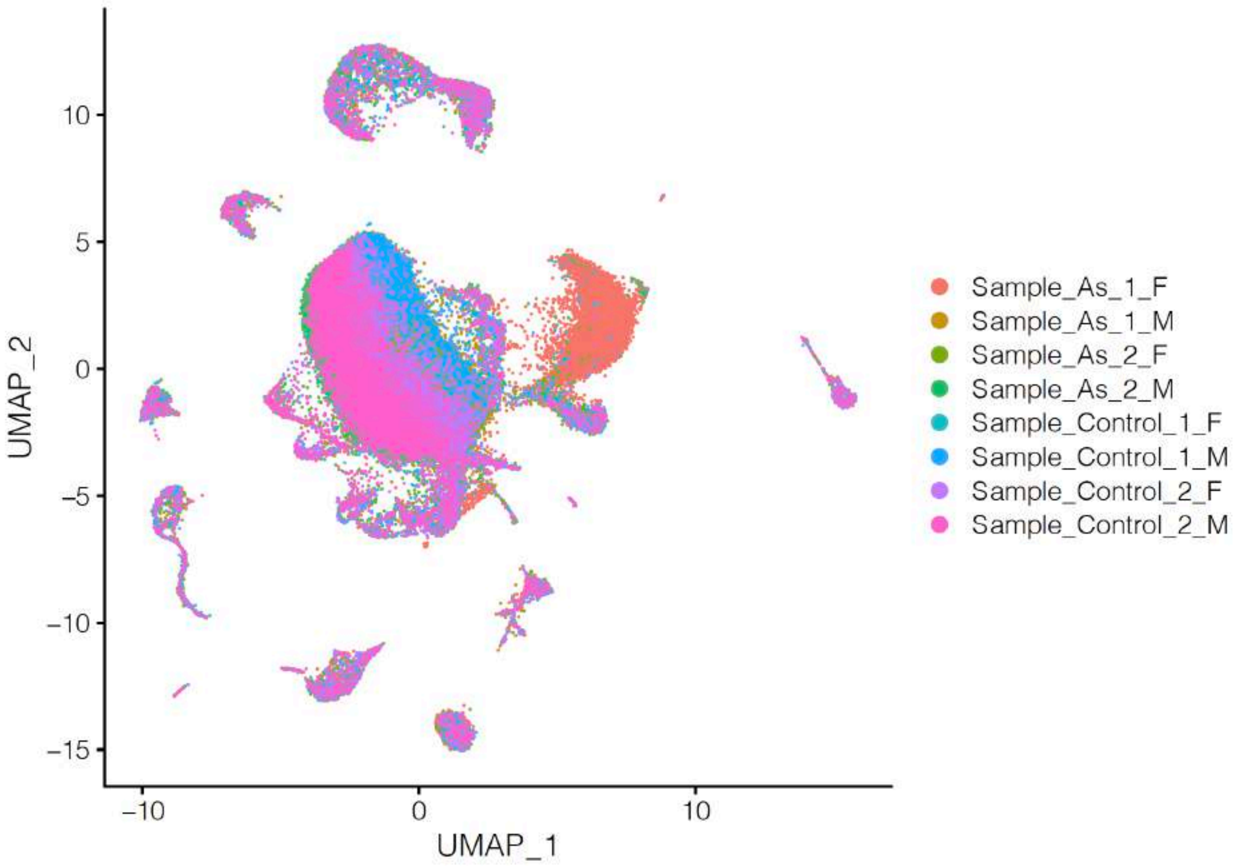

Supplementary Fig. 19. Initial UMAP by sample ID.

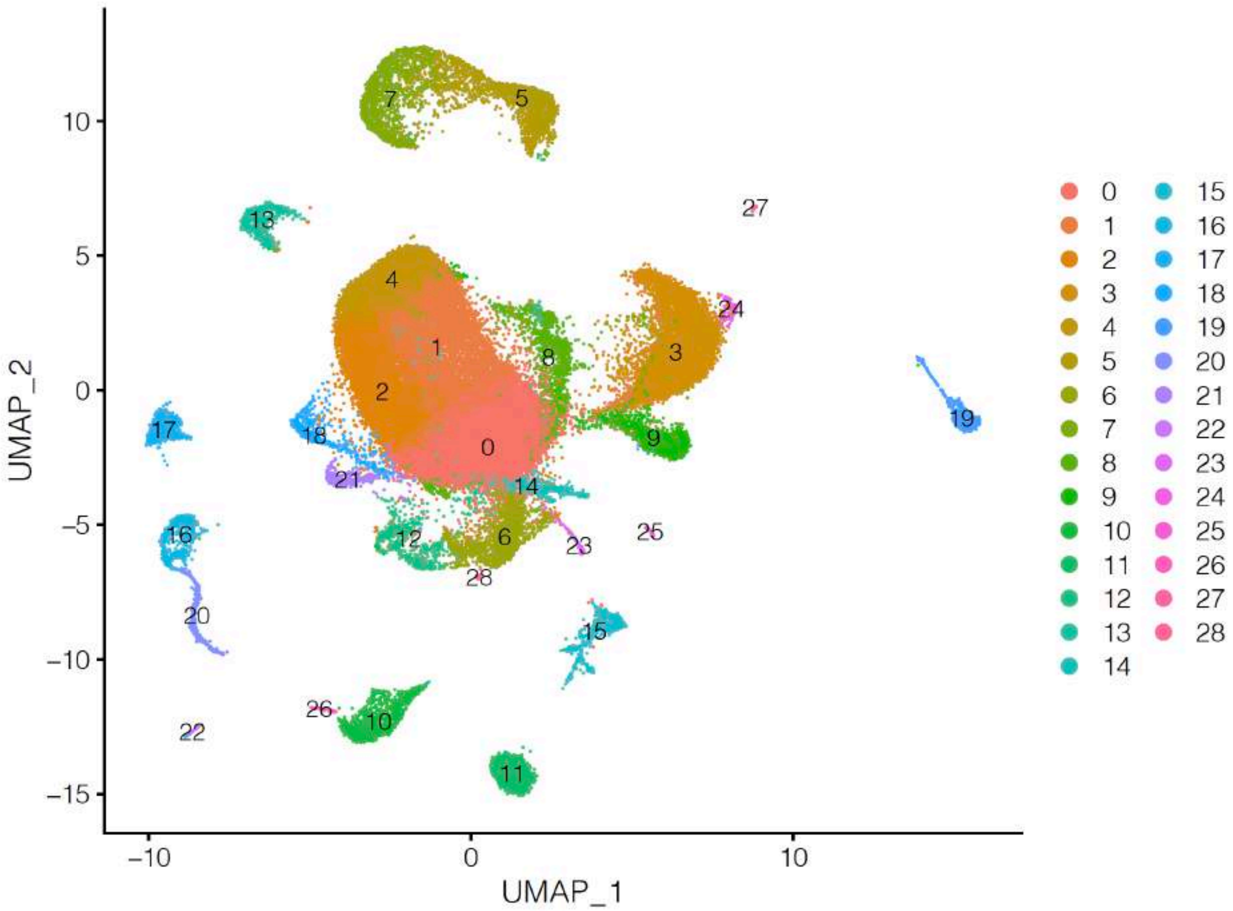

Supplementary Fig. 20. Initial UMAP with cell clustering

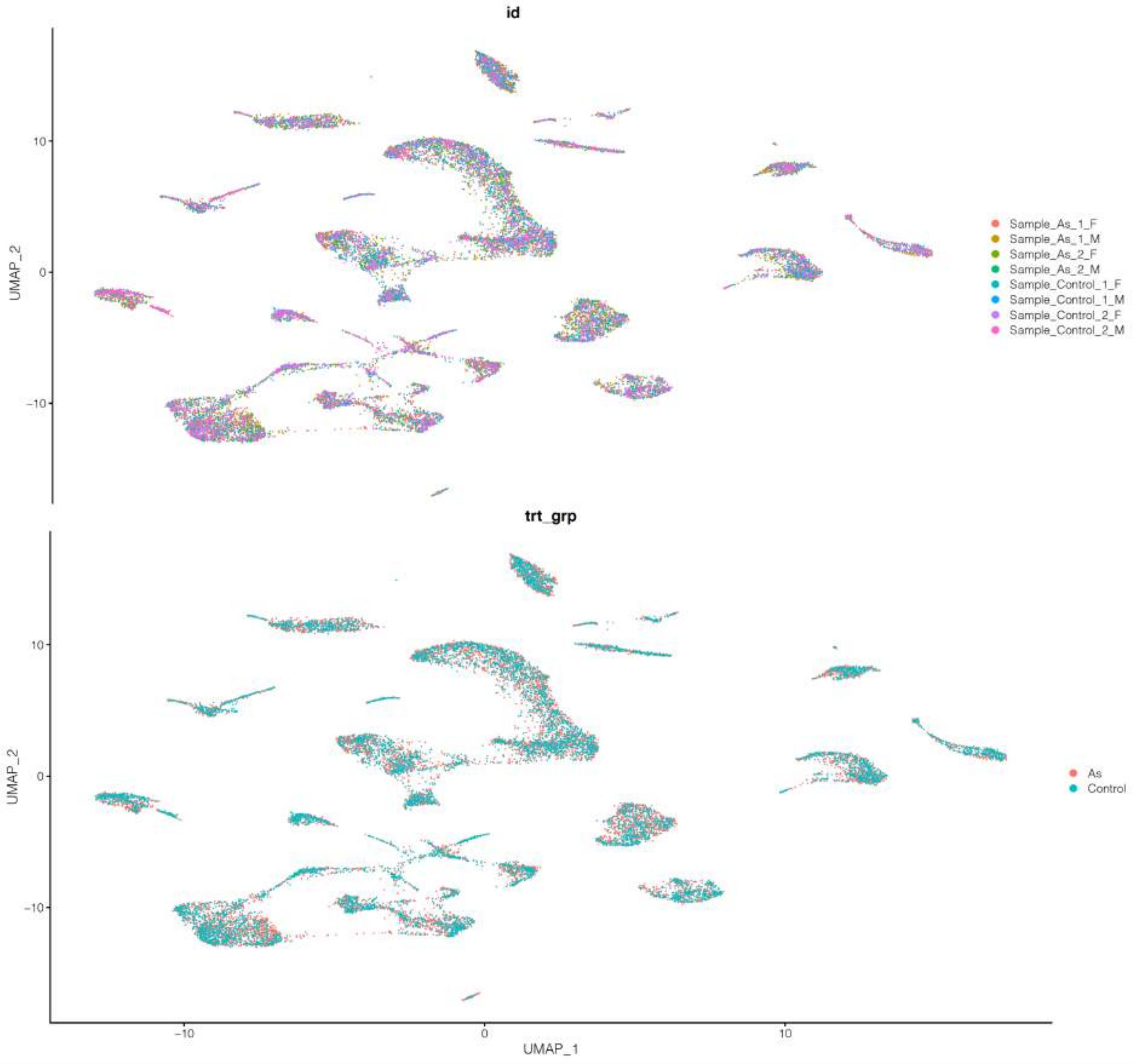

Supplementary Fig. 21. Final UMAP by sample ID and by treatment

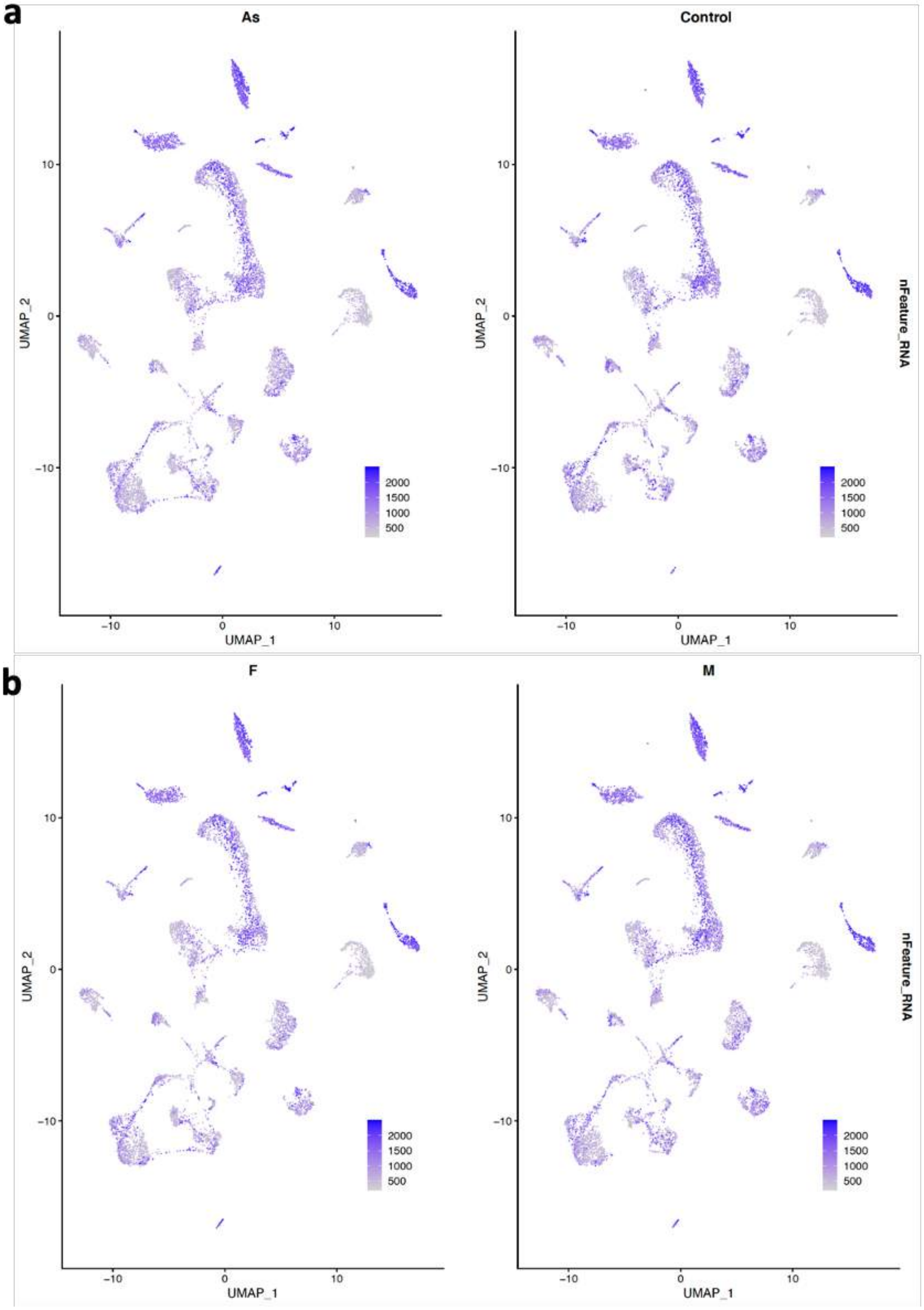

Supplementary Fig. 22. Number of genes expressed per cell by treatment group (a) and by sex (b)

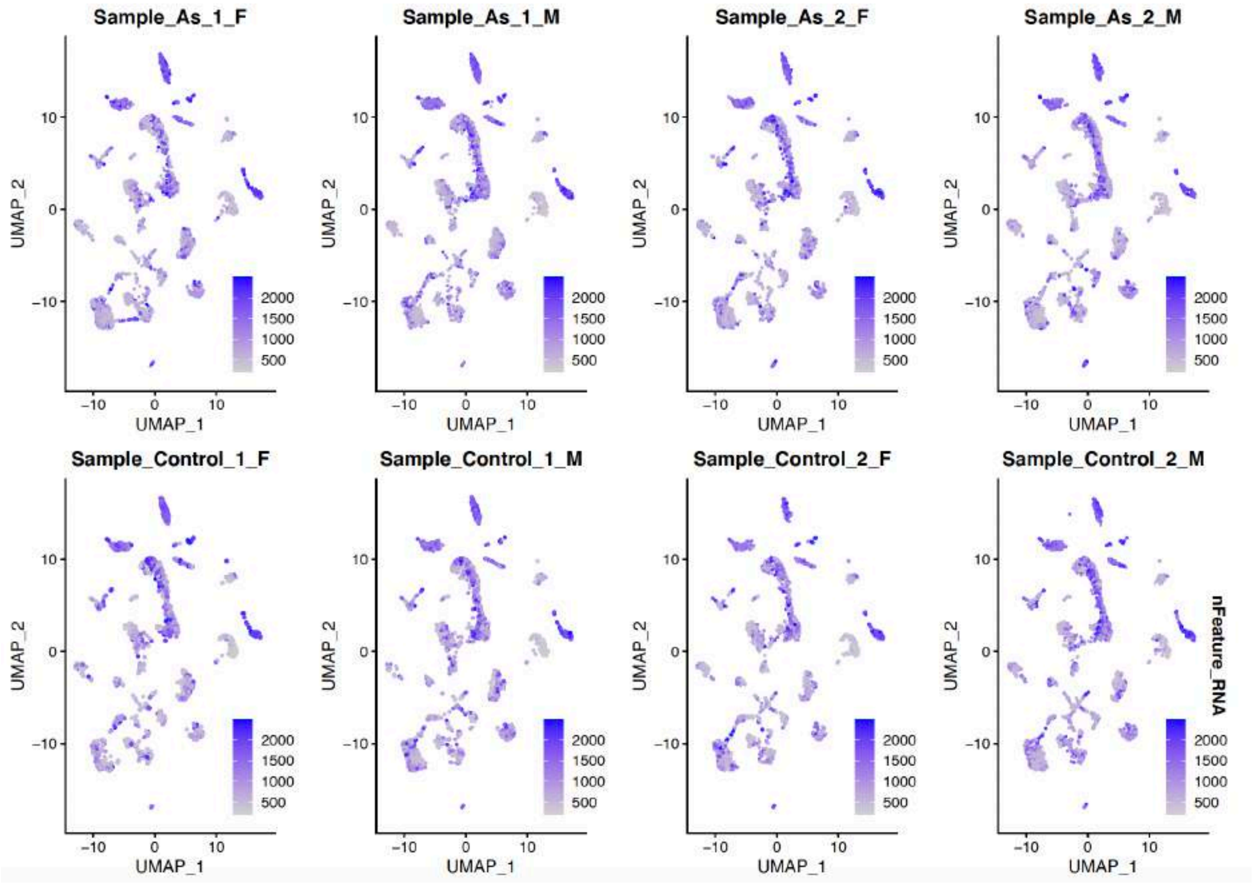

Supplementary Fig. 23. Number of genes expressed per cell by sample

Supplementary Tables

Supplementary Table 1. Comparative Analysis of Marker Gene Expression

Gene	Our candidate cell type	Marker Gene Cell Type From PMID 36928215[1]
Car2	GlyT	SpT
Car2	GlyT	Gly-T
Ceacam3	Spong_1	SpA-TGC
Fdx1	S-TGC	S-TGC_Precursor
Fdx1	S-TGC	S-TGC
Fnd3c2	S-TGC	S-TGC_Precursor
Fnd3c2	S-TGC	S-TGC
Fnd3c2	S-TGC	SpA-TGC
Nup62cl	S-TGC	LaTP_1
Nup62cl	S-TGC	S-TGC_Precursor
Nup62cl	S-TGC	S-TGC
Pappa2	Spong_1	Gly-T
Pappa2	Spong_1	SpA-TGC
Psg17	Spong_1	SpA-TGC
Psg25	Spong_1	SpA-TGC

Supplementary Table 2. The proportion of cells expressing *Xist* in each cell type

cell_type	prop_Xist
Macrophage_2	0.85
B Cell	0.77
T_Cell	0.77
Dendritic	0.73
Basophil	0.69
NK_Cell_1	0.56
Neutrophil_2	0.37
Neutrophil_1	0.19
Stromal_1	0.15
Decidual_2	0.04
Erythroid_Precursor_2	0.04
Erythroblast	0.04
Decidual_1	0.03
Labyr_Tropho_1	0.02
Macrophage_1	0.02
Spong_1	0.02
Tropho_Progenitor_2	0.01
Erythroid_Precursor_1	0.01
Erythroid_Precursor_3	0.01
Yolk_Sack	0.01
Spong_2	0.01
Endothelial_1	0.01
Endothelial_2	0.01
Endodermal_Cell	0.01
Labyr_Tropho_2	0.01
S-TGC_1	0.01
Megakaryocyte	0.01
Tropho_Progenitor_1	0.01
Pericyte	0.01
GlyT	0
Fibroblast	0
Labyr_Tropho_3	0
S-TGC_2	0
Pericyte_2	0
Stromal_2	0
NK_Cell_2	0

Supplementary Table 3. Comparative Analysis of Statistically Significant Genes

Current study			PMID 31752878[2]	
Mouse Gene	Cell Type	Adjusted p-value	Human Gene	p-value
Fzd4	B Cell	0.0019	*FZD4*	8.4e-4
Mgp	Spong_1	0.036	*MGP*	0.0037
Pdpn	Labyr_Tropho_3	0.0012	*PDPN*	0.012
Mef2c	Yolk_Sack	0.0076	*MEF2C*	0.012
Col1a2	Spong_2	0.018	*COL1A2*	0.014
Vim	Labyr_Tropho_3	2.7e-6	*VIM*	0.014
Unc5b	GlyT	0.017	*UNC5B*	0.019
Col3a1	Labyr_Tropho_2	0.041	*COL3A1*	0.023
Tagln	Spong_2	0.014	*TAGLN*	0.023
Cavin3	Labyr_Tropho_3	0.0032	*CAVIN3*	0.026
Tek	Decidual_2	1.5e-12	*TEK*	0.026
B3gnt9	Endothelial_2	0.003	*B3GNT9*	0.028
C1qtnf9	Neutrophil_1	0.02	*C1QTNF9*	0.037
Tmem184a	Yolk_Sack	0.018	*TMEM184A*	0.041
Fos	B Cell	0.0049	*FOS*	0.046

Supplementary Table 4. List of Unique DE genes (Arsenic vs Control)*

Cell type	gene	avg_log2FC	FC	pct.express. As.cells	pct.express. Control.cells	p_val_adj
B Cell	Gm43305	-0.6702256	0.6284084	0.179	0.48	4.34E-18
B Cell	Klf2	-0.5252845	0.69482209	0.969	0.97	2.93E-06
B Cell	Crem	0.40096975	1.32039516	0.575	0.396	5.26E-05
B Cell	Dusp1	-0.4839796	0.71500261	0.582	0.699	1.24E-04
B Cell	Fos	-0.4553273	0.72934471	0.881	0.911	1.56E-03
Basophil	H2-Ab1	-0.6262894	0.64784053	0.134	0.042	2.55E-05
Basophil	Mcpt8	1.95049804	3.86507937	0.857	0.417	1.51E-03
Basophil	Taldo1	0.84671223	1.79839786	1	1	3.39E-03
Basophil	Alox15	0.76250069	1.69642857	0.446	0	4.66E-03
Basophil	Fcer1a	1.0577155	2.08163265	0.741	0.208	1.68E-02
Basophil	Pirb	0.66025063	1.58035714	0.402	0	1.78E-02
Basophil	Srsf5	-1.0506593	0.4827475	1	1	2.97E-02
Basophil	Prr13	0.86043401	1.81558442	0.884	0.833	3.29E-02
Decidual_1	Gsn	0.37634866	1.29805243	0.385	0.243	3.70E-02
Erythroid_Precursor_1	Fth1	0.74713523	1.67845659	0.817	0.635	1.27E-03
Erythroid_Precursor_1	H3f3a	0.46379417	1.37916414	0.451	0.222	5.93E-03
Erythroid_Precursor_1	Hba-x	1.50471837	2.83769271	0.355	0.148	1.02E-02
GlyT	Car2	0.45164017	1.36759416	0.932	0.922	4.59E-02
Labyr_Tropho_1	Dmkn	-0.7860003	0.57994972	0.283	0.495	7.12E-04
Labyr_Tropho_2	Col3a1	0.36567569	1.28848495	0.732	0.472	9.00E-03
Labyr_Tropho_3	Anxa2	-0.8046412	0.57250443	0.397	0.705	3.76E-10
Labyr_Tropho_3	Gm42418	-0.4512259	0.73142107	1	1	1.77E-08
Labyr_Tropho_3	Tnnt2	-0.7549339	0.59257352	0.03	0.288	3.07E-07
Labyr_Tropho_3	Krt19	-0.8547185	0.5529732	0.508	0.799	3.84E-07
Labyr_Tropho_3	Vim	-0.9608446	0.51375604	0.553	0.755	1.88E-06
Labyr_Tropho_3	Bsg	-0.8387552	0.5591258	0.457	0.64	4.13E-06
Labyr_Tropho_3	Igf2	-0.7077221	0.61228611	0.824	0.914	4.21E-06
Labyr_Tropho_3	Krt7	-0.7930488	0.5771232	0.151	0.396	8.75E-06
Labyr_Tropho_3	Ceacam14	0.55615233	1.47034258	0.714	0.432	1.46E-05
Labyr_Tropho_3	Lgals3	-0.5864927	0.66595994	0.508	0.662	3.38E-05
Labyr_Tropho_3	Nupr1	-0.9385976	0.5217398	0.146	0.374	3.83E-05
Labyr_Tropho_3	Mt2	-0.9886899	0.5039352	0.397	0.619	5.84E-05
Labyr_Tropho_3	Sparc	-0.8986233	0.53639836	0.899	0.942	8.87E-05
Labyr_Tropho_3	Mt1	-1.2521977	0.41980822	0.819	0.82	9.95E-05
Labyr_Tropho_3	S100a4	-0.5997547	0.65986615	0.03	0.216	1.70E-04
Labyr_Tropho_3	Cavin1	-0.4585215	0.72773168	0.116	0.367	1.70E-04
Labyr_Tropho_3	Cav1	-0.5284903	0.69327983	0.276	0.504	2.93E-04
Labyr_Tropho_3	Tppp3	-0.439681	0.7372976	0.05	0.252	3.66E-04
Labyr_Tropho_3	Tpbpb	0.53467807	1.44861886	0.935	0.791	6.31E-04
Labyr_Tropho_3	Msln	-0.5683096	0.67440652	0.211	0.468	6.53E-04
Labyr_Tropho_3	Ceacam11	0.54311973	1.45712004	0.824	0.59	8.03E-04
Labyr_Tropho_3	H19	-0.5488221	0.68357803	0.935	0.978	1.76E-03
Labyr_Tropho_3	Igfbp6	-0.4629777	0.72548734	0.06	0.266	2.26E-03
Labyr_Tropho_3	Gpc3	-0.5507238	0.68267754	0.246	0.446	4.01E-03
Labyr_Tropho_3	Krt8	-0.4897467	0.71215014	0.553	0.705	4.66E-03
Labyr_Tropho_3	Prl7a2	0.4031878	1.32242674	0.482	0.23	1.22E-02
Labyr_Tropho_3	Emp3	-0.4225263	0.74611695	0.161	0.345	1.31E-02
Megakaryocyte	Apoa2	0.33071559	1.25763702	0.387	0.186	5.70E-03
NK_Cell_1	Gm10522	0.5711567	1.48571429	0.371	0	2.96E-02
Neutrophil_1	Hba-a2	-2.142038	0.22655952	0.451	0.442	1.67E-05
Neutrophil_1	Hmox1	-0.87641	0.54472123	0.244	0.329	8.55E-05
Neutrophil_1	Hba-a1	-1.8080328	0.28558007	0.503	0.463	1.55E-02
Neutrophil_1	Spag9	0.33568661	1.26197787	0.897	0.845	1.84E-02
Neutrophil_1	Mpp7	0.37787892	1.29943	0.743	0.595	1.94E-02
T_Cell	Hsp90aa1	-0.4801551	0.71690057	1	0.987	1.30E-02
Yolk_Sack	mt-Co1	-0.7331571	0.601586	0.673	0.783	1.03E-05
Yolk_Sack	Actb	-0.5457963	0.6850132	0.906	0.939	1.91E-05
Yolk_Sack	mt-Co2	-0.5709411	0.6731775	0.604	0.732	2.19E-03
Yolk_Sack	AY036118	0.3992023	1.31877853	0.956	0.894	1.36E-02

*Padj<0.05, FC >1.25 or FC <0.75. Differential expression testing was performed using MAST, as implemented in the Seurat package. P-values reported are two-sided and adjusted for multiple comparisons using the Benjamini-Hochberg method to control the False Discovery Rate.

Supplementary Table 5. ***Prap1*** **expression in Arsenic-treated mouse placentae (Male vs Female)***

Cell type	avg_log2FC	pct.express.M.cells	pct.express.F.cells	p_val_adj
B Cell	-0.7605725	0.018	0.408	1.76E-28
Basophil	-0.1599738	0.036	0.088	1.00E+00
Decidual_1	-1.1848664	0.067	0.481	1.58E-19
Decidual_2	-1.8963289	0.023	0.368	6.05E-11
Dendritic	-0.675498	0.062	0.424	1.55E-01
Endodermal_Cell	-0.5811136	0.174	0.506	2.25E-03
Endothelial_1	-1.1017259	0.095	0.429	1.72E-24
Endothelial_2	-1.5721244	0.085	0.492	1.29E-20
Erythroblast	-1.0524674	0.062	0.4	1.00E+00
Erythroid_Precursor_1	-0.9484824	0.12	0.389	1.28E-04
Erythroid_Precursor_2	-0.3454966	0	0.235	3.61E-04
Erythroid_Precursor_3	-0.6543037	0.012	0.407	5.31E-07
Fibroblast	-1.3024236	0.065	0.367	1.17E-06
GlyT	-0.8925364	0.079	0.414	2.46E-10
Labyr_Tropho_1	-1.1361273	0.175	0.43	8.09E-04
Labyr_Tropho_2	-0.9467567	0.085	0.323	1.00E-01
Labyr_Tropho_3	-2.4123151	0.121	0.4	4.77E-03
Macrophage_1	-1.2835527	0.127	0.474	7.03E-06
Macrophage_2	-0.7557022	0.032	0.481	9.04E-02
Megakaryocyte	-1.0121465	0.043	0.346	3.31E-08
NK_Cell_1	-0.5360529	0	0.3	1.00E+00
NK_Cell_2	-1.3792875	0.083	0.545	1.00E+00
Neutrophil_1	-0.6802661	0.038	0.387	4.46E-16
Neutrophil_2	-0.7352051	0.071	0.438	5.36E-07
Pericyte	-1.3993616	0.105	0.526	1.51E-18
Pericyte_2	-0.8266684	0.19	0.571	1.41E-01
S-TGC_1	-1.0750038	0.067	0.456	4.90E-22
S-TGC_2	-1.8182112	0.087	0.603	1.40E-15
Spong_1	-0.8019951	0.059	0.36	3.62E-17
Spong_2	-1.0561885	0.091	0.392	2.32E-21
Stromal_1	-3.5454417	0.075	0.466	1.60E-08
Stromal_2	-0.9724773	0.114	0.395	5.76E-02
T_Cell	-0.4240573	0.015	0.255	1.42E-04
Tropho_Progenitor_1	-0.8158219	0.068	0.36	2.07E-14
Tropho_Progenitor_2	-1.3838558	0.074	0.383	4.47E-06
Yolk_Sack	-1.4222179	0.101	0.514	1.47E-06

*Differential expression testing was performed using MAST, as implemented in the Seurat package. P-values reported are two-sided and adjusted for multiple comparisons using the Benjamini-Hochberg method to control the False Discovery Rate.

Supplementary Table 6. Selected DE genes (PRAP1 vs Control)

Gene	log2FC	Padj
CDKN1A	0.198	0.00074352
CEBPA	0.148	0.0102177
CCNE1	0.16	9.16E-09
CCNB1	0.106	0.00015095
LRP1	-0.242	3.94E-17
DYNC1H1	-0.296	5.65E-12
CENPF	-0.281	3.05E-09
HSP90AB1	-0.144	2.569E-06
HSPG2	-0.229	2.93355E-06
PDCD4	-0.251	1.31324E-05
HMMR	-0.341	2.11115E-05
DHCR24	-0.245	4.54813E-05
CDK7	-0.207	5.21528E-05
GRB2	-0.168	5.66769E-05
PLK1	-0.251	0.000272067
CAV1	-0.194	0.000279409
CALR	-0.105	0.000663999
DLGAP5	-0.273	0.000702655
TUBB	-0.102	0.000905936
TPX2	-0.17	0.001318908
CANX	-0.091	0.005457953
CDK1	-0.246	0.007642032
UBC	-0.151	0.007642032
AURKA	-0.185	0.020492245
PRR11	-0.174	0.020492245
AP2B1	-0.145	0.035439325

Supplementary Table 7. Primers sequences

hGAPDH	FW	ACAACTTTGGTATCGTGGAAGG
	RV	GCCATCACGCCACAGTTTC
hPRAP1	FW	ACAGCCTGTACCACCCTCC
	RV	AGCACCTGGTGATTTGGCATC
hSRY	FW	GCGTATTCAACAGCGATGATTAC
	RV	TCTCCCGTTTCACACTGATACTT
mSry	FW	ACAAGTTGGCCCAGCAGAAT
	RV	GGGATATCAACAGGCTGCCA
hAFP	FW	CCAACAGGAGGCCATGCTT
	RV	GAATGCAGGAGGGACATATGTTT
hAPOA2	FW	GTGTCAGCTCCTTTGACT
	RV	ATGGAGAAGGTCAAGAGCCC
hAPOB	FW	ACACACTGGACGCTAAGAGGA
	RV	ACTTGTGCTACCATCCCATACT
hCA2	FW	ATGGTCATGCTTTCAACGTGG
	RV	TGTCCATCAAGTGAACCCCAG
hGPX3	FW	CCTTCCTACCCTCAAGTATGTCC
	RV	AGGCGGTCAGATGTACCCA
hGUCA2B	FW	TCTGCCTCAGGACCTTCA
	RV	TTCACACACAGCTCACAGTC
hHAND1	FW	TCAAGGCTGAACTCAAGAAGG
	RV	TGCGTCCTTTAATCCTCTTC
hGATA4	FW	AGTCTCCACAGACCAGCTCC
	RV	AGGTCCGTGCAGGAATTTGAG

Supplementary References

1 Jiang, X. *et al*. A differentiation roadmap of murine placentation at single-cell resolution. *Cell Discov* **9**, 30 (2023). https://doi.org/10.1038/s41421-022-00513-z

2 Winterbottom, E. F. *et al*. Transcriptome-wide analysis of changes in the fetal placenta associated with prenatal Arsenic exposure in the New Hampshire Birth Cohort Study. *Environ Health* **18**, 100 (2019). https://doi.org/10.1186/s12940-019-0535-x
