# Supplementary material for: Single-cell RNA sequencing reveals placental response under environmental stress
Source: Nat Commun. 2024 Aug 2;15:6549. doi: 10.1038/s41467-024-50914-9 (PMC11297347; doi:10.1038/s41467-024-50914-9)
Supplement: Supplementary file 3 — Description of additional supplementary files [file 41467_2024_50914_MOESM3_ESM.pdf]

# Description of Additional Supplementary Files

**Supplementary Data 1.** Marker genes

**Supplementary Data 2.** Deferentially expressed genes (As vs Control)

**Supplementary Data 3.** Unique Deferentially expressed genes (As vs Control)

**Supplementary Data 4.** Deferentially expressed genes (Male vs Female) in Control cells

**Supplementary Data 5.** Deferentially expressed genes (Male vs Female) in Asexposed cells

**Supplementary Data 6.** RNA seq and Pathway Enrichment analysis in BeWo cells (PRAP1 vs Control)
