# Supplementary material for: Single-cell RNA sequencing reveals placental response under environmental stress
Source: Nat Commun. 2024 Aug 2;15:6549. doi: 10.1038/s41467-024-50914-9 (PMC11297347; doi:10.1038/s41467-024-50914-9)
Supplement: Supplementary file 10 — Reporting Summary [file 41467_2024_50914_MOESM10_ESM.pdf]

nature portfolio

Corresponding author(s): Hae-Ryung Park

Last updated by author(s): Jun 25, 2024

Reporting Summary

Nature Portfolio wishes to improve the reproducibility of the work that we publish. This form provides structure for consistency and transparency in reporting. For further information on Nature Portfolio policies, see our Editorial Policies and the Editorial Policy Checklist.

Statistics

For all statistical analyses, confirm that the following items are present in the figure legend, table legend, main text, or Methods section.

n/a	Confirmed	
☐	☒	The exact sample size (n) for each experimental group/condition, given as a discrete number and unit of measurement
☐	☒	A statement on whether measurements were taken from distinct samples or whether the same sample was measured repeatedly
☐	☒	The statistical test(s) used AND whether they are one- or two-sided<br>*Only common tests should be described solely by name; describe more complex techniques in the Methods section.*
☐	☒	A description of all covariates tested
☐	☒	A description of any assumptions or corrections, such as tests of normality and adjustment for multiple comparisons
☐	☒	A full description of the statistical parameters including central tendency (e.g. means) or other basic estimates (e.g. regression coefficient) AND variation (e.g. standard deviation) or associated estimates of uncertainty (e.g. confidence intervals)
☐	☒	For null hypothesis testing, the test statistic (e.g. F, t, r) with confidence intervals, effect sizes, degrees of freedom and P value noted<br>*Give P values as exact values whenever suitable.*
☐	☒	For Bayesian analysis, information on the choice of priors and Markov chain Monte Carlo settings
☐	☒	For hierarchical and complex designs, identification of the appropriate level for tests and full reporting of outcomes
☐	☒	Estimates of effect sizes (e.g. Cohen's d, Pearson's r), indicating how they were calculated

Our web collection on statistics for biologists contains articles on many of the points above.

Software and code

Policy information about availability of computer code

Data collection	Sequence data was generated using Illumina's NovaSeq 6000.
Data analysis	All statistical analysis was performed in R (version 4.0.2) using the Seurat package (version 4.1.1) or Graphpad Prism 10.

For manuscripts utilizing custom algorithms or software that are central to the research but not yet described in published literature, software must be made available to editors and reviewers. We strongly encourage code deposition in a community repository (e.g. GitHub). See the Nature Portfolio guidelines for submitting code & software for further information.

Data

Policy information about availability of data

All manuscripts must include a data availability statement. This statement should provide the following information, where applicable:

- Accession codes, unique identifiers, or web links for publicly available datasets
- A description of any restrictions on data availability
- For clinical datasets or third party data, please ensure that the statement adheres to our policy

The single-cell RNA-seqencing data generated in this study have been deposited in the ArrayExpress database under accession code E-MTAB-14214. The processed scRNA-seq data are available at ArrayExpress using the same accession code and on Zenodo at https://zenodo.org/records/10258020. The authors declare that all other data supporting the findings of this study are available within the paper and its supplementary information files. Source data are provided with this paper. Code needed to reproduce all analyses and figures using the single-cell RNA-sequencing data is available at https://github.com/edvanburen/placenta_code.

Research involving human participants, their data, or biological material

Policy information about studies with human participants or human data. See also policy information about sex, gender (identity/presentation), and sexual orientation and race, ethnicity and racism.

Reporting on sex and gender	N/A(De-identified placental tissues were used in this study)
Reporting on race, ethnicity, or other socially relevant groupings	N/A(De-identified placental tissues were used in this study)
Population characteristics	N/A(De-identified placental tissues were used in this study)
Recruitment	Placentas were anonymously collected from elective pregnancy terminations. Informed consent was obtained by the study resident physician. Patients did not receive any compensation as part of this study. All women of childbearing age (15-50 years of age) were included in this study.
Ethics oversight	Collection of tissues was approved by the Institutional Human Subjects Review Board at the University of Rochester (IRB ID#: 6740) .

Note that full information on the approval of the study protocol must also be provided in the manuscript.

Field-specific reporting

Please select the one below that is the best fit for your research. If you are not sure, read the appropriate sections before making your selection.

☒ Life sciences ☐ Behavioural & social sciences ☐ Ecological, evolutionary & environmental sciences

For a reference copy of the document with all sections, see nature.com/documents/nr-reporting-summary-flat.pdf

Life sciences study design

All studies must disclose on these points even when the disclosure is negative.

Sample size	Sample size was 3 or larger for all in vitro studies. The number of mice for scRNA seq was determined based on previously published single cell studies.
Data exclusions	No data was excluded.
Replication	All experiments were repeated at least three times.
Randomization	Animals were randomized for treatment with vehicle or arsenic.
Blinding	Investigators were blinded while doing IF staining and semi-quantification of mouse placental tissues.

Reporting for specific materials, systems and methods

We require information from authors about some types of materials, experimental systems and methods used in many studies. Here, indicate whether each material, system or method listed is relevant to your study. If you are not sure if a list item applies to your research, read the appropriate section before selecting a response.

Materials & experimental systems

n/a	Involved in the study	
☐	☒	Antibodies
☐	☒	Eukaryotic cell lines
☒	☐	Palaeontology and archaeology
☐	☒	Animals and other organisms
☒	☐	Clinical data
☒	☐	Dual use research of concern
☒	☐	Plants

Methods

n/a	Involved in the study	
☒	☐	ChIP-seq
☒	☐	Flow cytometry
☒	☐	MRI-based neuroimaging

Antibodies

Antibodies used	PRAP1 (11932-1-AP Proteintech), CAR2 (50695-RP02, Sino Biological), GUCA2B (ORB312251, Biorbyt), Ly6G (127606, BioLegend), beta-actin (643807, BioLegend)
Validation	PRAP1: https://www.ptglab.com/products/PRAP1-Antibody-11932-1-AP.htm<br>CAR2:https://www.sinobiological.com/antibodies/mouse-carbonic-anhydrase-ii-50685-rp02?_gl=1*1qtz10h*_up*MQ..*_ga*MTA3NTY5NTM5MS4xNzE5MzQxMDY0*_ga_HYV7JHQNBH*MTcxOTM0MTA2My4xLjEuMTcxOTM0MTA2Ny4wLjAuMA..<br>GUCA2B:https://www.biorbyt.com/guca2b-antibody-orb312251.html<br>Ly6G: https://www.biolegend.com/en-us/products/fitc-anti-mouse-ly-6g-antibody-4775<br>beta-actin:https://www.biolegend.com/en-us/products/direct-blot-hrp-anti-beta-actin-antibody-12776

Eukaryotic cell lines

Policy information about cell lines and Sex and Gender in Research

Cell line source(s)	HTR-8/SVneo (CRL-3271, female) and BeWo (CCL-98, male) cell lines were purchased from ATCC. Swan 71 cells (female) were kindly provided by Dr. Gil Mor (Wayne State University).<br>Human induced pluripotent stem cells (iPSCs, SKU: 30HU-002) were procured from iXCells Biotechnologies, San Diego, CA, sourced from healthy male (Lot: 400531) and female (Lot: 400530) donors.
Authentication	Authentication was done from ATCC or iXCells Biotechnologies.
Mycoplasma contamination	All cell lines were negative for Mycoplasma contamination.
Commonly misidentified lines (See ICLAC register)	No commonly misidentified cell lines were used in the study.

Animals and other research organisms

Policy information about studies involving animals; ARRIVE guidelines recommended for reporting animal research, and Sex and Gender in Research

Laboratory animals	C57BL/6 mice were purchased from the Jackson Laboratory (Bar Harbor, ME).
Wild animals	N/A
Reporting on sex	This study was about the effect of placental responses to environmental stress, thereby, female pregnancy mice were exposed to arsenic. For scRNA-seq, the sex of each placenta (corresponding to fetal sex) was determined and both sexes were included for the analysis.
Field-collected samples	N/A
Ethics oversight	All mice experiments were performed in accordance with relevant guidelines and regulations at the University of Rochester (UCAR-2021-004). Throughout the duration of the experiment, all animals were housed under controlled temperature and lighting conditions with ad libitum access to food and water. Animal welfare was monitored daily by qualified veterinary staff to ensure their health and well-being. Any signs of distress or illness were addressed promptly under the supervision of veterinary personnel. Euthanasia was performed using CO2 inhalation followed by cervical dislocation. All mice procedures were overseen by certified staff to ensure it was conducted humanely and with minimal suffering.

Note that full information on the approval of the study protocol must also be provided in the manuscript.

Plants

Seed stocks	N/A
Novel plant genotypes	N/A
Authentication	N/A
